# Ion absorption, distribution and salt tolerance threshold of three willow species under salt stress

**DOI:** 10.3389/fpls.2022.969896

**Published:** 2022-08-02

**Authors:** Xin Ran, Xiaoxi Huang, Xiao Wang, Haiyong Liang, Yanchao Wang, Jiajing Li, Zihan Huo, Bingxiang Liu, Changming Ma

**Affiliations:** ^1^Department of Forest Cultivation, College of Forestry, Hebei Agricultural University, Baoding, China; ^2^College of Bioscience and Engineering, Xingtai University, Xingtai, China; ^3^Hebei Urban Forest Health Technology Innovation Center, Baoding, China

**Keywords:** ion absorption, NaCl stress, salt tolerance mechanism, *Salix matsudana*, *Salix gordejevii*, *Salix linearistipularis*, transport and distribution, threshold

## Abstract

To investigate the response mechanism and salt tolerance threshold of three willow seedlings (*Salix matsudana, Salix gordejevii, Salix linearistipularis*), the absorption, transport and distribution of salt ions (Na^+^, K^+^, Ca^2+^) were studied under hydroponic conditions with different salt concentrations (CK, 171, 342, 513, and 684 mm) and treatment times (1, 3, 5, 8, 11, and 15 days). *Salix linearistipularis* has the weakest ability to maintain its apparent shape, while *Salix matsudana* has the strongest ability. The three plants have a certain Na^+^ interception ability, and the interception abilities of *Salix matsudana* and *Salix gordejevii* are higher than that of *Salix linearistipularis*. The leaf S_*AK*,*Na*_ of *Salix linearistipularis* were higher than those of *Salix matsudana* and *Salix gordejevii*. The leaf selection ability was the highest, and the selection ability of the root system was the lowest in *Salix linearistipularis*. The long-term low salt concentration and the short-term high salt concentration can increase the root and leaf salinity. *Salix matsudana* grows more stably in a long-term high-salt stress environment, and *Salix gordejevii* grows stably in a short-term high-salt stress environment. However, *Salix linearistipularis* is more suitable for planting as an indicative plant because of its sensitivity to salt stress. The root Na^+^ content of *Salix matsudana* and *Salix gordejevii* was 34.21 mg/g, which was the maximum root retention capacity. Once the accumulation of Na^+^ content in roots exceeds this value, the rejection capacity of roots is broken through, and the selective ion absorption capacity will rapidly become weak, which easily leads to the death of plants.

## Introduction

Soil salinization poses a serious threat to sustainable agricultural production and ecological balance (Litalien and Zeeb, 2020; Fahad et al., 2021b,e). It is predicted that by 2050, nearly 50% of the world’s arable land will be salinized, which will be a huge threat to sustainable agricultural development and food security (Butcher et al., 2016). Soil salinity, as a major abiotic stress factor, seriously inhibits the growth and development of plants (Fahad et al., 2015b; Zhu et al., 2020). It is also considered a physiological drought phenomenon (Hasan et al., 2021). To achieve sustainable development of saline soil agriculture, people can only choose two methods: one from the soil itself affected by salt and the other comes from the plants themselves (Shao et al., 2019). Many scholars have also taken different measures for the improvement of saline-alkali land. For example, they installed hidden pipes to drain water (Ayars et al., 2006; Feng et al., 2017) or used gypsum as a soil conditioner to improve the soil (Zhao et al., 2019; Sheikh et al., 2022). However, studies have shown that planting saline-alkali-tolerant plants has the advantages of low investment, wide application and strong sustainability. Therefore, biological treatment has become the optimal measure for the improvement and utilization of saline-alkali lands (Djanaguiraman et al., 2006; Li et al., 2009).

Regarding the research on salt-tolerant plants, scholars mostly use hydroponics to study the growth and physiological characteristics of plants under different concentrations (Li et al., 2021), ion distribution (Rabiei et al., 2020) and the mechanism of salt tolerance (Bian and Lu, 2022). Salt stress affects plant growth and development in two ways: osmotic stress and ion imbalance. The former results in a decrease in chloroplast photosynthetic rate and CO_2_ concentration, and a limitation of stomata (Wang et al., 2018; Ma et al., 2022). The latter can lead to unstable inflow and outflow of Na^+^, K^+^, and Ca^2+^ in cells (Yang and Guo, 2018; Zhang et al., 2022). Currently, many scholars have examined the effects of salt stress on the growth of different plants at different levels from different disciplines (such as ecology, phytophysiology, and molecular biology of the cell). They also discussed the mechanism of salt damage to plants (Aghaleh et al., 2011; Fahad et al., 2020; Hou et al., 2021) and noticed plant responses to salt stress (Fahad et al., 2015a; Karunasinghe et al., 2020; Kim et al., 2020; Shu et al., 2020; Altaf et al., 2021; Wu et al., 2022). Willow is a general term for Salix and Chosenia tree species in the Salicaceae family. It has the characteristics of fast growth, cold resistance, drought resistance, salinity resistance and pollution resistance. It also has ecological, timber and biomass energy value (Warmiński et al., 2021). Regarding the salt tolerance of willow, studies have mostly focused on root vigor, growth, salt tolerance, plant phenotype, physiological substances, and molecular biology. They observed a series of phenomena under salt stress, including root activity, plant growth index, and plant height decrease with increasing stress (Sui, 2006; Markus-Michalczyk et al., 2014; Tian and Li, 2018; Feng et al., 2020; Zhang et al., 2021). However, studies on the salt distribution on plant roots and leaves and salt tolerance thresholds are still not in-depth and systematic (Maggio et al., 2007; Kurunc et al., 2020; Banakar et al., 2022). Moreover, there are few studies on the growth and physiological characteristics of salt-tolerant plants at different times. What are the ion-selective absorption characteristics of different willow roots and leaves and the threshold for breaking the ion balance? What are the responses and correlations of Na^+^, K^+^, and Ca^2+^ (or ratios) of roots and leaves of different varieties of willow to salt stress? Do different willows resist salt stress through the same salt adaptation mechanism?

To explore the above problems, this paper selected three species of willow (*Salix matsudana*, *Salix gordejevii*, *Salix linearistipularis*) as experimental materials under the condition of greenhouse hydroponics. First, the effects of salt stress on plant growth and Na^+^, K^+^, and Ca^2+^ uptake, transport and distribution in root and leaf tissues were studied. Second, the correlation between salt ions and their ratios was analyzed, and the differences in salt tolerance mechanisms were discussed. Finally, the salt tolerance thresholds of the three willow trees were fitted according to the above data. This study provides new insights for the analytical evaluation of salt tolerance of willow and lays a foundation for further development of salt tolerance breeding strategies.

## Materials and methods

### Experimental materials

The experiment was performed in the artificial climate chamber of the West Campus of Hebei Agricultural University (115^°^25′ E, 38^°^48′ N), Nanshi District, Baoding City, Hebei Province. According to the existing research, when the light intensity is 1,000 μmol⋅m^–2^⋅s^–1^, the growth and photosynthetic capacity of plants are the best (Liu et al., 2013, 2022; Gerona et al., 2019; Zhu H. L. et al., 2019; Zhang G .C. et al., 2020; Ma et al., 2022). There are also many scholars who keep the relative humidity at 50–70% when setting up the experiments. Therefore, according to the existing research and the actual situation, we set the corresponding parameters of the climate chamber. The set temperature of the climate chamber was 28^°^C/25^°^C (light/dark). The LED cold light source controlled the light intensity at 1,000 μmol⋅m^–2^⋅s^–1^, the photoperiod was 14 h/10 h (light/dark), and the relative humidity was 60%.

The experimental materials were collected from the germplasm resource nursery of Jinshatan Forest Farm. In early spring of 2020, annual cuttings of 3 willow species *Salix matsudana*, *Salix gordejevii*, and *Salix linearistipularis* were collected before germination. They were basically uniform, robust and free of pests and diseases. After harvesting, they were stored in a freezer at 0^°^C. On June 26, 2020, a water culture experiment was conducted in the artificial climate chamber. The middle two-thirds of the branches were selected and cut into 20 cm cuttings. The uppermost bud was 0.5–1 cm from the top of the cuttings. The upper cut was a flat cut, and the lower cut was an oblique cut. Sufficient cuttings of the three species of willows were placed in the clean water for natural growth and routine management. On July 15, when their growth reached the treatment requirement (the average root length was approximately 5 cm), the seedlings with basically uniform roots were selected for stress treatment.

### Experimental design

**FIGURE 1:**
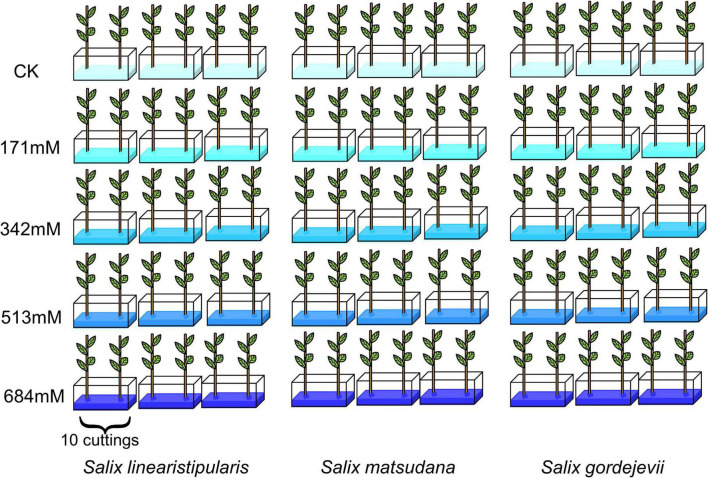
Design of three willows experimental treatment.

The experimental materials were placed in a 55 cm × 38 cm × 15 cm (length × width × height) plastic box for water culture (Figure 1). We refer to the articles of some scholars who set the salt concentration between 100 and 700 mM. In addition, we also converted the salt concentration into international units according to the formula (Gao et al., 2022; Ni et al., 2022). Studies have found that a certain concentration of Hoagland nutrient solution has a good promotion effect on seedling growth of plants (Zobolo and Khumalo, 2017). When setting up experiments on the effects of salt stress on plants, some scholars take 1/2 Hoagland solution as the base solution and then add the corresponding concentration of salt (Regni et al., 2019). Therefore, we decided to use this method to set the salt concentration. Hydroponic solutions with NaCl concentrations of 171, 342, 513, and 684 mM were prepared based on 1/2 Hoagland complete nutrient solution, and 1/2 Hoagland complete nutrient solution was used as the control (pH 7.2). Each treatment was repeated 3 times, and 10 seedlings of each species were directly placed in the solution, with more than half of the height drowning in the solution. The nutrient solution was changed every 5 days during their growth. In order to prevent excessive accumulation of salt, we took out the seedlings and carefully rinsed off the residual salt from the roots with clean water before changing the nutrient solution. 1/2 Hoagland’s complete nutrient solution includes: NH_4_H_2_PO_4_ 57.5 mg, FeSO_4_ 15 mg, Ca(NO_3_)_2_⋅4H_2_O 472.5 mg, MgSO_4_ 246.5 mg, NaFeC_10_H_12_N_2_O_8_⋅3H2O 30 mg, CuSO_4_ 0.05 mg, H_3_BO_3_ 2.86 mg, H_8_MoN_2_O_4_ 0.02 mg, Na_2_B_4_O_7_⋅10H_2_O 4.5 mg, MnSO_4_ 2.13 mg, ZnSO_4_ 0.22 mg, K_2_SO_4_ 303.5 mg.

We randomly selected three seedlings with even growth vigor on the 1st, 3rd, 5th, 8th, 11th, and 15th days of the stress test for the observation of various growth physiological indexes. The measurement of each index was repeated for 3 times, and the growth status was observed and recorded 1 week after the end of the experiment.

### Measured indices and methods

#### Root and leaf phenotypic records

Following salt stress treatment, plants at different salt concentrations were taken out of the hydroponic tank intact at the indicated times. To avoid damaging the root system, rinse the solution with running water. After drying, the plants were placed in the EPSEN v700 (Seiko Epson Corporation, Japan) scanner for scanning. During the period, the camera was used to slightly adjust the position of the root to avoid overlapping and crossing of the root system. Scanned photographs of intact plants are used as final analysis images and are saved in TIFF format. A picture of the shape change of the plant under salt stress can be obtained.

#### Ion contents in roots and leaves, ion selective absorption and transport ratio

The method for ion content determination was slightly modified compared to the methods proposed by Yang (2010) and Meng et al. (2020). The sample was first baked at 105^°^C for 30 min and then dried at 70–80^°^C to a constant weight. After it was ground into powder, the fixed mass was weighed. After 30 ml of deionized water was added, the sample was shaken well and placed in a boiling water bath for 2 h. After cooling, the sample was filtered and diluted to 50 ml. The Na^+^, K^+^, and Ca^2+^ contents were determined by the atomic absorption method. By reference to the methods proposed by Yang et al. (2003); Xia et al. (2018), and Zhang et al. (2018), the following formula was used to calculate the selective absorption and transport coefficients of ions X (K^+^ and Ca^2+^) by roots and leaves, where ion absorption coefficient according to the Eqs. 1 and ion transport coefficient according to the Eqs. 2. In the formula, the K^+^ content was mg/L, and the Ca^2+^ content was 230 mg/L in the medium (culture solution). The larger the value of SA_*X,Na*_, the stronger the root system’s ability to inhibit Na^+^ and promote × absorption. The higher the ST_*X,Na*_ value is, the stronger the root inhibition capacity of Na^+^ and promotion of × transport to leaves is. “Medium” refers to the medium (such as soil, culture medium, etc.) in which plant roots grow.


(1) $${\mathrm{SA}}_{X,\mathrm{Na}}=\text{Root}\,\left(X|{\mathrm{Na}}^{+}\right)/\text{Medium}\,\left(X|{\mathrm{Na}}^{+}\right)$$

(2) $${\mathrm{STX}}_{X,\mathrm{Na}}=\text{Leaf}\,\left(X|{\mathrm{Na}}^{+}\right)/\text{Root}\,\left(X|{\mathrm{Na}}^{+}\right)$$

### Data processing

Excel and Origin were used to fit the response curve data. SPSS 18.0 data processing software was used for statistical analysis, and the least significant difference (LSD) method was used to check the significance of the difference when the *P*-value was less than 0.05.

## Results

### Changes in the growth state of seedlings under salt stress

**FIGURE 2:**
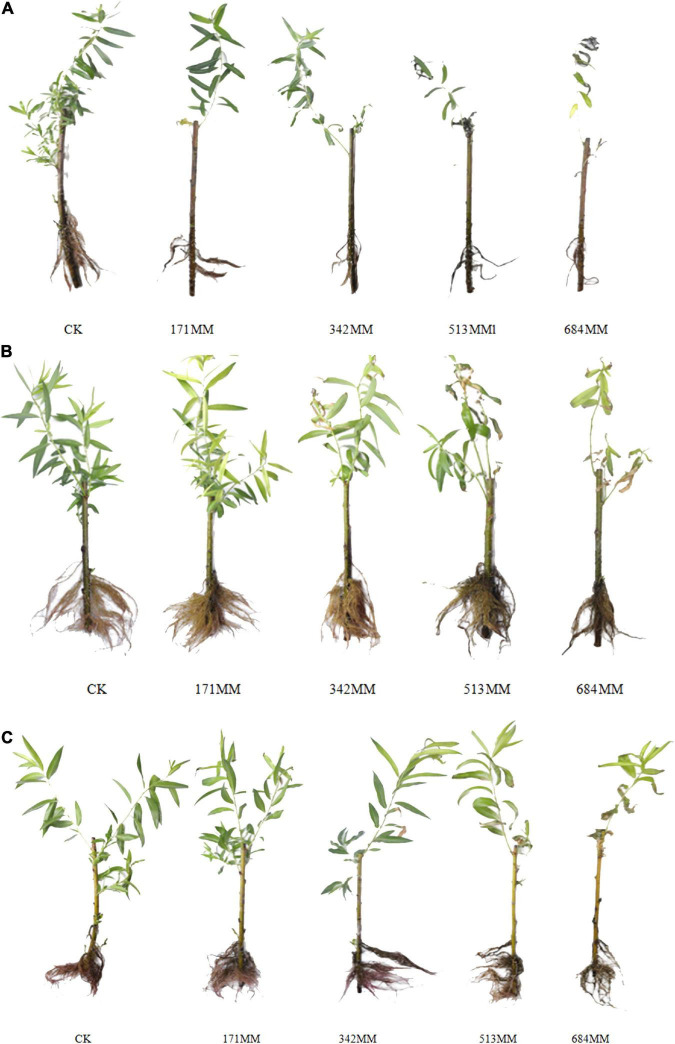
Phenotypic response of three willow seedlings to salt stress. **(A)** *Salix linearistipularis*; **(B)** *Salix matsudana*; **(C)** *Salix gordejevii*.

Figure 2 shows that salt stress had a direct impact on the growth state of plants. The leaves of the seedlings of the three species of willow all showed that with the aggravation of salt stress, the damage degree gradually increased, leaf tips and leaf margins gradually turned yellow, leaf margins became scorched and curled, leaves gradually lost their green color and even wilted and fell off, and the twigs became dry. The number of roots of the three species of willow gradually decreased with the aggravation of salt stress. Figures 2A–C also shows that the roots of *Salix linearistipularis*, *Salix gordejevii* and *Salix matsudana* significantly reduced at NaCl concentrations of 171, 513, and 684 mM, respectively, and they became obviously withered at NaCl concentrations of 171, 342, and 342 mM, respectively, and the leaves significantly reduced at NaCl concentrations of 171, 342, and 513 mM, respectively. The number and length of the roots of the three species of willow all decreased, and the sensitivity in descending order was *Salix linearistipularis*> *Salix gordejevii*>*Salix matsudana*.

Overall, the root and leaf tissues of *Salix linearistipularis* suffered the earliest and the most serious damage, followed by *Salix gordejevii* and *Salix matsudana*. There were differences in the growth changes among different species of willow. In comparison, *Salix matsudana* maintained a better apparent morphology under salt stress, followed by *Salix gordejevii* and *Salix linearistipularis*.

### Changes in Na^+^, K^+^, and Ca^2+^ contents in roots and leaves under salt stress

**FIGURE 3:**
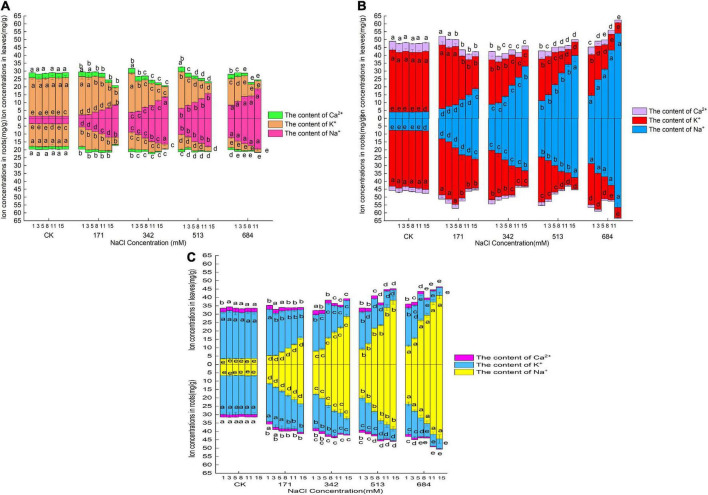
Effects of salt stress on Na^+^, Ca^2+^, and K^+^ contents in roots and leaves of three willows. **(A)** *Salix linearistipularis*; **(B)** *Salix matsudana*; **(C)** *Salix gordejevii*.

Figure 3 shows that under normal circumstances (CK group), the K^+^ content was the highest in the roots and leaves of the three species of willow, followed by Na^+^ and Ca^2+^. The Na^+^ content in the roots and leaves of *Salix linearistipularis* was the lowest, and the K^+^ and Ca^2+^ contents in the roots and leaves of *Salix matsudana* were the highest. With the aggravation of salt stress and prolonged stress time, the Na^+^ contents in the roots and leaves of the three species of willow showed a cumulatively increasing trend. Salt stress significantly increased the Na^+^ contents in the roots and leaves, and the Na^+^ contents in the roots were always higher than those in the leaves, but the differences gradually decreased with the increase in stress degree and the prolongation of stress time (Figures 3D–F). Under different salt concentrations and times, the increase in Na^+^ content in the roots and leaves was ranked in descending order: *Salix matsudana*, *Salix gordejevii*, and *Salix linearistipularis*. Under the same salt stress, the Na^+^ contents and changes in the roots and leaves of *Salix linearistipularis* were lower than those of *Salix gordejevii* and *Salix matsudana*.

It can be seen from the Figure 3 that the K^+^ contents in the roots and leaves of the three willow species decreased with the aggravation of stress and prolonged stress time, and the K^+^ content in the roots was lower than that in the leaves. Among the three tree species, the K^+^ contents in the roots and leaves of *Salix matsudana* were higher than those of *Salix gordejevii* and *Salix linearistipularis*. With the increase in salt concentration and the prolongation of stress time, the K^+^ contents in the roots of *Salix matsudana* and *Salix gordejevii* decreased more slowly than those of *Salix linearistipularis*.

With the aggravation of salt stress and the prolongation of stress time, the Ca^2+^ contents in the roots and leaves of the three species of willow showed a decreasing trend (Figure 3). Among the three tree species, under normal growth conditions, the Ca^2+^ contents in the leaves and roots of *Salix matsudana* were higher than those of *Salix gordejevii* and *Salix linearistipularis*. After salt stress, *Salix gordejevii* maintained the strongest Ca^2+^ content, and the content in the leaves showed a gentler decreasing trend. With the aggravation of salt stress and prolonged time, the Na^+^ content in roots and leaves continued to increase, while the K^+^ and Ca^2+^ contents continued to decrease. The long-term stress at low salt concentrations and the short-term stress at high salt concentrations both could make salinity in roots and leaves reach high levels.

**FIGURE 4:**
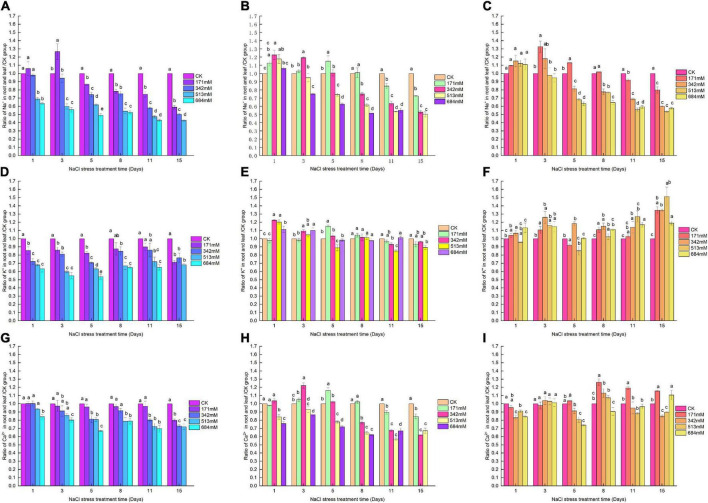
Ratios of Na^+^, Ca^2+^, K^+^ to CK in roots/leaves of three willows under salt stress. Ratio of Na^+^ to CK in roots/leaves of three willows. **(A)** *Salix linearistipularis*; **(B)** *Salix matsudana*; **(C)** *Salix gordejevii*. Ratio of K^+^ to CK in roots/leaves of three willows. **(D)** *Salix linearistipularis*; **(E)** *Salix matsudana*; **(F)** *Salix gordejevii*. Ratio of Ca^2+^ to CK in roots/leaves of three willows. **(G)** *Salix linearistipularis*; **(H)** *Salix matsudana*; **(I)** *Salix gordejevii*. Different small letters indicate significant differences between treatments at 0.05 level among treatment.

In the first 3, 8, and 8 days, with the increase of salt stress, *Salix linearistipularis*, *Salix matsudana* and *Salix gordejevii* showed a trend of increasing first and then decreasing (Figures 4A–C). The higher the concentration was, the more significant they differed from CK. Compared with CK, the ratio of K^+^ in the root/leaf of *Salix linearistipularis* with different salt concentrations in the first 5 days showed a gradually decreasing trend, and the difference was significant (Figure 4D). When the NaCI was 171 mM, compared with CK, the root/leaf K^+^ of *Salix matsudana* was no significant difference with CK except that 171 mM was significantly increased on the fifth day (Figure 4E). At the concentration of 342 mM, the *Salix gordejevii* showed a significant increase compared with CK with the prolongation of time (Figure 4F). From the 3rd day, the Ca^2+^ in the root/leaf of *Salix linearistipularis* under 513 mM NaCl showed a significant decreasing trend with the prolongation of time compared with CK (Figure 4G). Compared with CK, the Ca^2+^ in the root/leaf of *Salix matsudana* showed a trend of increasing at first and then decreasing with time at different salt concentrations (Figure 4H). At 171 mM salt concentration, the Ca^2+^ in the root/leaf of *Salix gordejevii* showed an upward trend with time, and there was no significant difference until the 8th day (Figure 4I).

### Selective absorption and transport of ions in the roots and leaves of three tree species under salt stress

**FIGURE 5:**
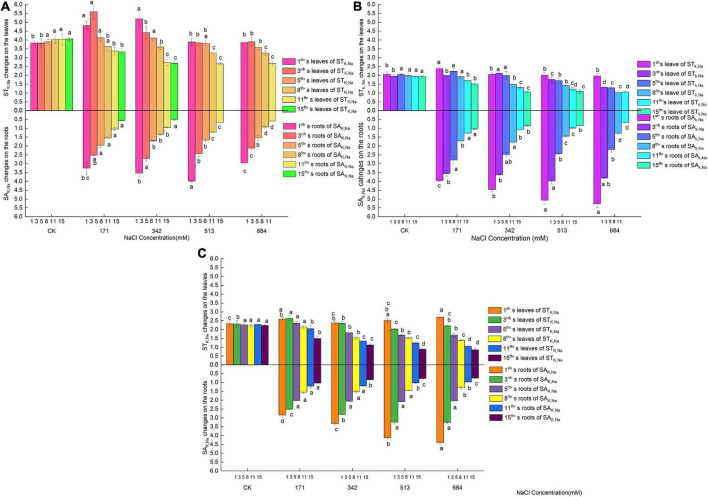
Changes of K^+^ absorption and transport in roots and leaves of three willow species. **(A)** *Salix linearistipularis*; **(B)** *Salix matsudana*; **(C)** *Salix gordejevii*.

In the early stage of salt stress (1–3 days), the SA_*K*,*Na*_ of the roots of *Salix linearistipularis* only had a temporary rise at a salt concentration of 513 mM on the first day, and the roots of *Salix matsudana* and *Salix gordejevii* had increased SA_*K*,*Na*_ with increasing salt concentration (Figures 5A–C). During this period, the ST_*K*,*Na*_ of the leaves of the three species of willow increased slightly at low salt concentrations (171 mM or 342 mM) and then decreased. Then, from the 5th day, the SA_*K*,*Na*_ of the roots and leaves of the three species of willow showed an overall balance or gradual decrease with increasing salt concentration and prolonged stress time. The higher the salt concentration and the longer the time was, the more significant the difference was. For *Salix matsudana* and *Salix gordejevii*, the SA_*K*,*Na*_ of their roots were higher than those of their leaves at the beginning of salt stress, but with the increase in salt concentration and prolonged stress time, the SA_*K*,*Na*_ of their roots were finally lower than those of their leaves. During the change, *Salix matsudana* maintained a longer time than *Salix gordejevii*, and the SA_*K*,*Na*_ of roots were higher than those of leaves. For *Salix linearistipularis*, the SA_*K*,*Na*_ of roots were lower than those of leaves. Under the same NaCl treatment level, *Salix linearistipularis* had higher SA_*K*,*Na*_ of leaves than *Salix matsudana* and *Salix gordejevii*, and with the increase in salinity, the SA_*K*,*Na*_ of leaves of *Salix linearistipularis* decreased slowly and were inferior to those of *Salix matsudana* and *Salix gordejevii*. Although the SA_*K*,*Na*_ of the roots of the three tree species decreased in a similar trend with increasing salinity, *Salix linearistipularis* had an obliviously lower decline than *Salix matsudana* and *Salix gordejevii*. In general, under salt stress, *Salix linearistipularis* had the highest SA_*K*,*Na*_ of leaves but the lowest SA_*K*,*Na*_ of roots.

**FIGURE 6:**
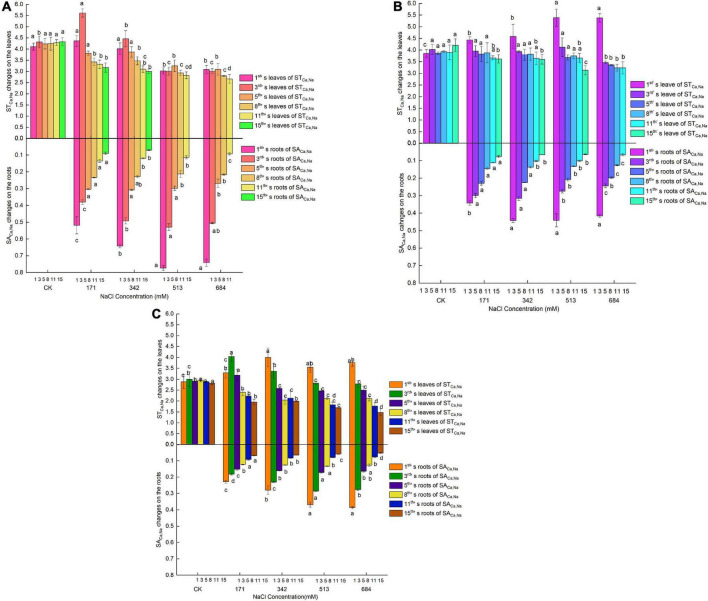
Changes of Ca^2+^ absorption and transport in roots and leaves of three willow species. **(A)** *Salix linearistipularis*; **(B)** *Salix matsudana*; **(C)** *Salix gordejevii*.

Under normal growth conditions, the three tree species had basically the same SA_*Ca*,*Na*_ (Figures 6A–C). On the first day of salt stress, the SA_*Ca*,*Na*_ of the leaves of *Salix matsudana* increased with the increasing amounts of salt treatment. *Salix gordejevii* showed different degrees of increase after 1–5 days of treatment, and its increasing trend was affected by both stress concentration and stress time, with an increase in a short time (1 day) and a tendency of selective increase at low concentrations with the extension of time (3–5 days). The SA_*Ca*,*Na*_ of *Salix linearistipularis* showed a transient increase at a salt concentration of 171 mM on the 3rd day and then continued to decrease. Then, the SA_*Ca*,*Na*_ of the leaves of the three species of willow decreased gradually with increasing salt concentration and over time. Large disparities existed among the three in the SA_*Ca*,*Na*_ of roots. With the increase in the salt concentration, the increased SA_*Ca*,*Na*_ of *Salix gordejevii* lasted for 8 days, followed by *Salix linearistipularis* (3 days) and *Salix matsudana* (1 day). Under the same treatment level, *Salix linearistipularis* presented the highest SA_*Ca*,*Na*_ of roots, followed by *Salix matsudana* and *Salix gordejevii*. All three species had much higher SA_*Ca*,*Na*_ in the leaves than in the roots. In general, under salt stress, *Salix matsudana* and *Salix gordejevii* maintained higher SA_*Ca*,*Na*_ of leaves among the three species, but the SA_*Ca*,*Na*_ of their roots were at a lower level. *Salix linearistipularis* had the highest SA_*Ca*,*Na*_ of roots and the lowest SA_*Ca*,*Na*_ of leaves.

### Changes in Na^+^/K^+^ and Na^+^/Ca^2+^ contents in the roots and leaves of the three tree species under salt stress

**FIGURE 7:**
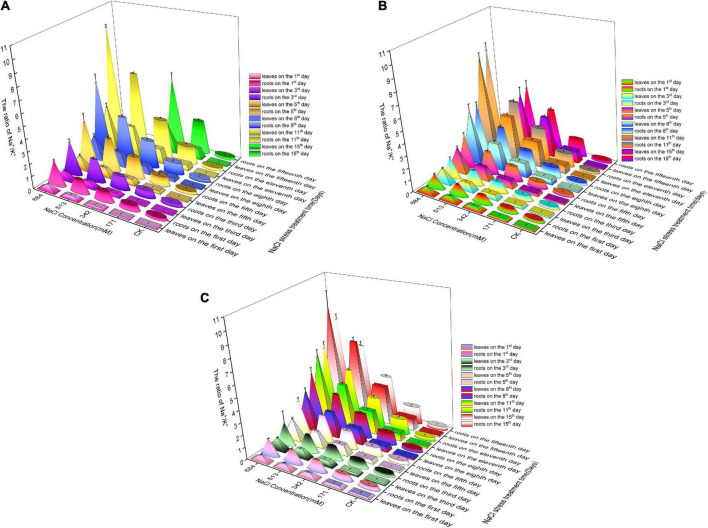
Effects of salt stress on Na^+^/K^+^ in roots and leaves of three willow species. Changes of Na^+^/K^+^ in roots and leaves of three willow species. **(A)** *Salix linearistipularis*; **(B)** *Salix matsudana*; **(C)** *Salix gordejevii*.

With the aggravation of salt stress and the passage of time, the Na^+^/K^+^ and Na^+^/Ca^2+^ contents in roots and leaves increased significantly (Figure 7). Long-term salt stress at low salt concentrations and short-term salt stress at high salt concentrations could both make salinity in roots and leaves reach relatively high levels. At different salinity levels, the Na^+^/K^+^ and Na^+^/Ca^2+^ contents in the roots were generally higher than those in the leaves, indicating that plants can reduce the damage of salt stress to aboveground tissues by regulating ion transport. No significant difference existed among *Salix linearistipularis*, *Salix matsudana*, and *Salix gordejevii* in Na^+^/K^+^ contents in roots, but *Salix linearistipularis* had apparently lower Na^+^/K^+^ contents in roots than *Salix matsudana* and *Salix gordejevii*, which indicates a strong effect on the inhibition of Na^+^ or selective absorption of K^+^ by its leaves (Figures 7A–C).

**FIGURE 8:**
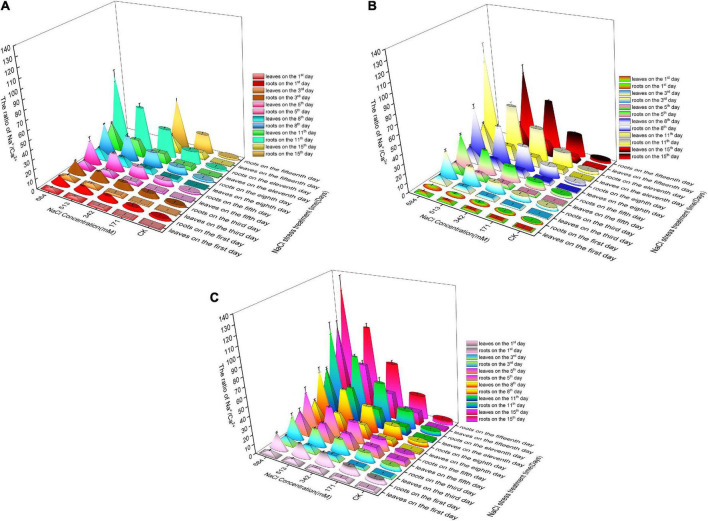
Effects of salt stress on Na^+^/Ca^2+^ in roots and leaves of three willow species. **(A)** *Salix linearistipularis*; **(B)** *Salix matsudana*; **(C)** *Salix gordejevii*.

On the first day of salt stress, the growth rate of Na^+^/Ca^2+^ in different concentrations of *Salix gordejevii* root was higher than that of the other two willows compared with CK (Figures 8A–C). Among them, the growth rate of Na^+^/Ca^2+^ in the root system of *Salix matsudana* is the lowest. In the first 3 days, the Na^+^/Ca^2+^ of willow root leaves showed an increasing trend compared with CK, but the difference was not significant. With the increased salt stress, the growth rate of Na^+^/Ca^2+^ contents in the leaves and roots of *Salix gordejevii* were higher than those of *Salix linearistipularis* and *Salix matsudana*. This indicates that with the increased stress, the relative absorption of Na^+^ by *Salix gordejevii* increases greatly, while its selective absorption of Ca^2+^ becomes relatively weakened.

### Correlation of Na^+^, Na^+^/K^+^, and Na^+^/Ca^2+^ contents in the roots and leaves of three tree species under salt stress

**FIGURE 9:**
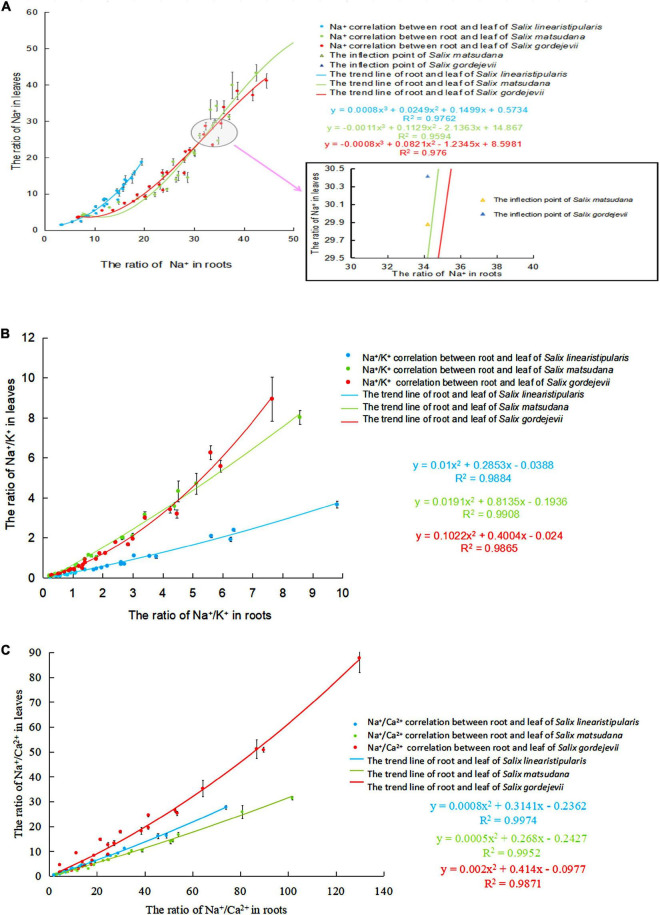
Correlation of Na^+^, Na^+^/K^+^, and Na^+^/Ca^2+^ in roots and leaves of three willows under salt stress. **(A)** Na^+^ correlation; **(B)** Na^+^/K^+^ correlation; **(C)** Na^+^/Ca^2+^ correlation.

Taking the Na^+^, Na^+^/K^+^, and Na^+^/Ca^2+^ contents in leaves as dependent variables and Na^+^, Na^+^/K^+^, and Na^+^/Ca^2+^ contents in roots as independent variables for curve estimation, we built correlation equations. Figure 9A shows that with the aggravation of salt stress, the Na^+^ contents in roots gradually increased, and the Na^+^ transported from roots to leaves also increased. *Salix linearistipularis* had the highest degree of Na^+^ correlation between roots and leaves (Eq. 3; *R*^2^ = 0.9762). *Salix gordejevii* ranked second (Eq. 4) (*R*^2^ = 0.976). *Salix matsudana* presented the lowest degree of correlation (Eq. 5) (*R*^2^ = 0.9594). The three tree species presented higher root-leaf Na^+^/K^+^ correlations than root-leaf Na^+^ correlations (Figure 9B). *Salix matsudana* presented the highest degree of correlation (Eq. 6) (*R*^2^ = 0.9908). *Salix linearistipularis* ranked second (Eq. 7) (*R*^2^ = 0.9884). *Salix gordejevii* presented the lowest degree of correlation (Eq. 8) (*R*^2^ = 0.9865). Among the three tree species (Figure 9C), *Salix linearistipularis* presented the highest root-leaf Na^+^/Ca^2+^ correlation (Eq. 9) (*R*^2^ = 0.9974), followed by *Salix matsudana* (Eq. 10) (*R*^2^ = 0.9952) and *Salix gordejevii* (Eq. 11) (*R*^2^ = 0.9871).


(3) $$\text{y}=0.0008\,x^{3}+0.0249\,x^{2}+0.1499\,x^{1}+0.5734$$

(4) $$\text{y}=-0.0008\,x^{3}+0.0821\,x^{2}-1.2345\,x^{1}+8.5981$$

(5) $$\text{y}=-0.0011\,x^{3}+0.1129\,x^{2}-2.1363\,x^{1}+14.867$$

(6) $$\text{y}=0.0191\,x^{2}+0.8135\,x^{1}-0.1936$$

(7) $$\text{y}=0.01\,x^{2}+0.2853\,x^{1}-0.0388$$

(8) $$\text{y}=0.1022\,x^{2}+0.4004\,x^{1}-0.024$$

(9) $$\text{y}=0.0008\,x^{2}+0.3141\,x^{1}-0.2362$$

(10) $$\text{y}=0.0005\,x^{2}+0.268\,x^{1}-0.2427$$

(11) $$y=0.002\,x^{2}+0.414\,x^{1}-0.0977$$

In this study, the root-leaf Na^+^ accumulative concentration was used to fit the correlation equation, and the threshold of Na^+^ absorption and rejection by the roots of *Salix matsudana* and *Salix gordejevii* was obtained as the fitting inflection point. The research results showed that when the Na^+^ content in the roots of *Salix matsudana* reached 34.21 mg/g and the Na^+^ content in leaves reached 29.88 mg/g, and when the Na^+^ content in the roots of *Salix gordejevii* reached 34.21 mg/g and the Na^+^ content in leaves reached 30.42 mg/g, the two willow species had the maximum root rejection capacity.

## Discussion

### Effects of salt stress on the phenotypes of three willow species

Under salt stress, plants adjust their own physiological and biochemical reactions, change their morphological structure and the selectivity of the protoplasmic membrane to absorb or reject ions, and increase the water content in their bodies to improve their saline-alkaline resistance (Guo et al., 2020; Zhang G .C. et al., 2020; Fahad et al., 2021a,c,f; Shah et al., 2021). Roots are the first organs of plants to contact, sense and respond to the salt environment (Tan et al., 2020). Under salt stress, the number and volume of roots of Zea mays L. both decrease (Wang et al., 2020). The results of this study also verified this conclusion: The time when roots change reflects the sensitivity of plants to salt stress. The yellowing and abscission of plant leaves is the most direct manifestation of the state of salt stress (Jimenez-Casas and Zwiazek, 2014). The higher the NaCl concentration is, the more serious the damage to plant leaves is. The degree of damage in descending order is *Salix linearistipularis* > *Salix gordejevii* > *Salix matsudana*. The change of leaf phenotype in this paper is not only related to the excessive accumulation of Na^+^ in plant leaves, but also the low content of K^+^ and Ca^2+^, which is related to the toxicity to plants. Moreover, salt stress is actually a physiological drought phenomenon. Plants reduce water consumption by weakening the binding ability between pigment and pigment protein, degrading chlorophyll, reducing photosynthesis, and closing stomata to reduce water consumption, thereby maintaining their own stability.

### Changes in Na^+^, K^+^, and Ca^2+^ contents in roots and leaves of three species under salt stress

Under salt stress, Na^+^ is the main harmful ion that limits plant growth and causes salt damage (Li et al., 2019). The mechanism of ion accumulation and transport in plants under salt stress has always been a hot research topic. The localization of ions, the interception at roots, and the inhibition of transport to shoots are the main ways in which plants respond to salt stress (Storey et al., 2003). In this experiment, compared with the control, salt stress increased the Na^+^ contents in the roots and leaves of the seedlings, and the Na^+^ contents in the roots were higher than those in the leaves. This result shows that the three plants all have a certain Na^+^ interception ability. But there are some differences in interception ability. *Salix linearistipularis* has a stronger interception ability than *Salix matsudana* and *Salix gordejevii*. In this study, with the aggravation of salt stress and prolonged time, the Na^+^ contents in root and leaf tissues increased significantly, and the corresponding K^+^ contents continued to decrease, which was mainly affected by the accumulation of Na^+^. In addition, Na^+^ presents an apparent competitive inhibitory effect on the absorption and active sites of K^+^, which caused plants under salt stress to suffer from damage both from Na^+^ and insufficient K^+^ (Shabala and Cuin, 2008). Therefore, K^+^ content in roots and leaves of plants decreased with the aggravation of salt stress (Li et al., 2022).

The imbalance of Ca^2+^ is the primary response to NaCl stress. In this study, with the aggravation of salt stress and prolonged time, the Ca^2+^ of the three willows was continuously replaced by Na^+^, with the contents of Ca^2+^ decreasing continuously. However, there were differences among tree species, and the Ca^2+^ stabilization ability of *Salix gordejevii* was higher than that of *Salix matsudana* and *Salix linearistipularis*. This is related to the difference in the selective absorption capacity of Ca^2+^ among different tree species after salt stress. The uptake of ions by plants in response to salt stress is affected by the plant species, stress intensity and time.

### Responses of selective absorption and transport of irons to salt stress

The ion selective transport coefficient represents the selective ability of plants to ion upward transport. The greater the selective transport coefficient of nutrient ions under salt stress is, the stronger the ability of plants to promote the upward transport of nutrient ions and inhibit the upward transport of salt ions is. The more salt ions that are trapped in the root, the stronger its salt tolerance is Teakle et al. (2007). Liu et al. (2014) found that the SA_*K*,*Na*_ and SA_*Ca*,*Na*_ of the roots of Elaeagnus angustifolia were much higher than those of other tissues. After salt stress, the SA_*K*,*Na*_ and SA_*Ca*,*Na*_ of leaves increased, showing a higher ability of Elaeagnus angustifolia to selectively transport K^+^ and Ca^2+^ from roots to leaves. Wu et al. (2017) found that the SA_*K*,*Na*_ and SA_*Ca*,*Na*_ of the leaves and roots of blueberry experienced a process of decrease-increase-decrease.

In this study, the selective absorption capacity of K^+^ of the three willow species was affected by the variety, location and time. In the early stage of salt stress, the selective absorption capacity of K^+^ is enhanced to reject Na^+^ to maintain ion balance. This effect is stressful and has limited ability. With the increase in stress concentration and prolonged time, the accumulation of Na^+^ in plants will cause ion imbalance until the selective absorption capacity of K^+^ is lost. The SA_*K*,*Na*_ significantly vary according to different varieties and parts. *Salix linearistipularis* presents higher SA_*K*,*Na*_ of leaves than those of *Salix matsudana* and *Salix gordejevii*, but the SA_*K*,*Na*_ of its roots are at the lowest level, which may be related to the different salt tolerance mechanisms of different willows.

The three willows had a selective absorption capacity of Ca^2+^ similar to that of K^+^. Although *Salix gordejevii* has outstanding performance in the SA_*Ca*,*Na*_ enhancement of roots and leaves, it is still lower than that of *Salix linearistipularis* and *Salix matsudana*. *Salix linearistipularis* presented the highest SA_*Ca*,*Na*_ of roots and the lowest SA_*Ca*,*Na*_ of leaves. Under high salt stress, the selective transport capacity of ions of the three willow trees decreased, possibly due to the accumulation of Na^+^ causing smaller stomatal opening of plant leaves, decreased transpiration, increased cell membrane permeability and ion toxicity.

### Effects of salt stress on the Na^+^/K^+^ and Na^+^/Ca^2+^ ratios of roots and leaves

Na^+^ has a strong competitive effect on the absorption of K^+^. The strategy of most salt-tolerant plants is to prevent excessive Na^+^ from entering cells or accelerate Na^+^ excretion while increasing the absorption of K^+^ and transporting it upward to reduce salt damage (Wakeel et al., 2011). Ca^2 +^ participates in plant stress resistance signal transduction and regulates the response of plants to stress changes (Orhan Şenol et al., 2009; Cao et al., 2020). The ratios of Na^+^/K^+^ and Na^+^/Ca^2+^ are often used to represent the degree of damage to ion balance caused by salt stress (Poustini and Siosemardeh, 2004; Manaa et al., 2013; Yang and Guo, 2018).

In this study, the Na^+^/K^+^ and Na^+^/Ca^2+^ contents in the roots and leaves of the three tree species increased significantly with increasing NaCl stress concentration and stress duration, which is consistent with the ion metabolism characteristics of *Hordeum vulgare* and wheat (Mahlooji et al., 2018; Guo et al., 2021). The reason is that the influx of Na^+^ will directly interfere with and inhibit the absorption and transport of K^+^ and Ca^2+^ by the cytoplasmic membrane, resulting in ion imbalance, and the ratio of Na^+^/K^+^ and Na^+^/Ca^2+^ of each organ will rise. This study also found that long-term stress at low salt concentrations and short-term stress at high salt concentrations could both lead to higher levels of salinity in roots and leaves. At different salt levels, the ratio of roots Na^+^/K^+^ and Na^+^/Ca^2+^ was generally higher than that of leaves. This shows that all three willow trees can reduce the damage of salt stress to aboveground tissues by regulating the selection and transport of ions.

### Analysis of root-leaf ion ratio correlation and threshold under salt stress

The root system is the first organ to face salt stress. The nutrient ions of the aerial part come from transport by roots (Fahad and Bano, 2012). In this study, Lowess (William, 1979) was used to fit the correlation equations of Na^+^, Na^+^/K^+^, and Na^+^/Ca^2+^ contents in roots and leaves. Overall, the three species of willow had the highest correlation of Na^+^/Ca^2+^ contents in roots and leaves, followed by Na^+^/K^+^ and then Na^+^. It may be that the influx of Na^+^ affects the many ions that plants already have. Ions also interact with each other. Studies have confirmed that the presence of Ca^2+^ can also inhibit the efflux of K^+^ (Guo et al., 2015), which leads to a high Na^+^/Ca^2+^ correlation and a low Na^+^ correlation.

The salt adaptation mechanism of plants mainly includes salt avoidance and salt tolerance. The former mainly avoids the accumulation of large amounts of salt ions by means of salt rejection, salt dilution or salt excretion. The latter can tolerate higher concentrations of salt ions in their bodies. The determination of the salt tolerance threshold of plants mainly focused on *Suaeda salsa*, *Plantago coronopus* (L.) and seagrass. They used the salt concentration at the beginning of a decline in the survival and growth rate of plants as a threshold (Koyro, 2006; Koch et al., 2007; He et al., 2017). Plant roots are directly exposed to Na^+^, and the Na^+^ content in roots accumulates with increasing salt concentration and the passage of time. Once the accumulation of Na^+^ contents in roots exceeds the threshold value and the rejection capacity of the roots is broken through. Then a large amount of Na^+^ enters the leaves, and the selective ion absorption capacity becomes weak, which easily leads to the death of the plant. *Salix linearistipularis* has a different mechanism in response to salt stress. It is a salt-rejecting tree species, and the Na^+^ content range is very narrow for its roots and leaves. The Na^+^ contents in leaves increased with the content increase in roots (the second derivation of the equation). It manifests itself as the selectivity of leaf leaves for Na^+^ absorption does not diminish with the increase of root Na^+^. Therefore, it cannot be fitted to its threshold with leaf and root Na^+^ content.

## Conclusion

Based on the above research and analysis, there are differences among three willows in ion absorption and distribution to different degrees under salt stress. The number of roots decreased with increased salt stress, followed by wilted, yellowed, curled, and dropped leaves. The selective absorption of Ca^2+^ and K^+^by the three willow species was affected by variety, location and time, and the selective absorption was stressful and had limited regulation ability. During the stress process, the Na^+^/K^+^ and Na^+^/Ca^2+^ in root and leaf organs were significantly increased. The Na^+^/Ca^2+^ had the highest correlation.

When the Na^+^ contents in the roots of *Salix matsudana* and *Salix gordejevii* reached 34.21 mg/g, the interception of Na^+^ in their roots reached the maximum. The range of Na^+^ contents in the roots and leaves of *Salix linearistipularis* is very narrow, so it is unable to fit thresholds with Na^+^ contents in roots and leaves. In general, *Salix matsudana* and *Salix gordejevii* are salt-tolerant, and they grow stably under long-term high salt and short-term low salt environment, respectively. However, *Salix linearistipularis* is more suitable for planting as an indicative plant because of its sensitivity to salt stress.

Currently, some studies have shown that there is a strong correlation between the ion flow rate and various physiological indicators. In the future, we will use non-invasive micro-testing (NMT) technology to further explore the dynamic transport process of ion flux in willow cells and the mechanism of salt tolerance.

## Data availability statement

The original contributions presented in the study are included in the article/Supplementary material, further inquiries can be directed to the corresponding authors.

## Author contributions

XR performed conceptualization. XR and BL performed writing-original draft. XW performed investigation. XH, CM, and BL performed data curation. HL and BL performed resources. CM and BL performed methodology. BL, YW, ZH, and JL performed formal analysis. BL performed funding acquisition, writing-review, and editing. All authors commented on previous versions of the manuscript, read and approved the final manuscript, and contributed to the study conception and design.
