# Supplementary material for: Ion absorption, distribution and salt tolerance threshold of three willow species under salt stress
Source: Front Plant Sci. 2022 Aug 2;13:969896. doi: 10.3389/fpls.2022.969896 (PMC9379094; doi:10.3389/fpls.2022.969896)

Supplementary Material

# Summary

# Table S1 Effects of salt stress on Na^+^, Ca^2+^ and K^+^ contents in roots and leaves of *Salix* *linearistipularis*.(data in Fig.3A)

**Table S2** Effects of salt stress on Na^+^, Ca^2+^ and K^+^ contents in roots and leaves of *Salix* *matsudana*.(data in Fig.3B)

**Table S3** Effects of salt stress on Na^+^, Ca^2+^ and K^+^ contents in roots and leaves of *Salix* *gordejevii*.(data in Fig.3C)

**Table S4** Ratio of Na^+^ to CK in roots/leaves of *Salix* *linearistipularis*.(data in Fig.4A)

**Table S5** Ratio of Na^+^ to CK in roots/leaves of *Salix* *matsudana*.(data in Fig.4B)

**Table S6** Ratio of Na^+^ to CK in roots/leaves of *Salix* *gordejevii*.(data in Fig.4C)

**Table S7** Ratio of K^+^ to CK in roots/leaves of *Salix* *linearistipularis*.(data in Fig.4D)

**Table S8** Ratio of K^+^ to CK in roots/leaves of *Salix* *matsudana*.(data in Fig.4E)

**Table S9** Ratio of K^+^ to CK in roots/leaves of *Salix* *gordejevii*.(data in Fig.4F)

**Table S10** Ratio of Ca^2+^ to CK in roots/leaves of *Salix* *linearistipularis*.(data in Fig.4G)

**Table S11** Ratio of Ca^2+^ to CK in roots/leaves of *Salix* *matsudana*.(data in Fig.4H)

**Table S12** Ratio of Ca^2+^ to CK in roots/leaves of *Salix* *gordejevii*.(data in Fig.4I)

# Table S13 Changes of K^+^ absorption and transport in roots and leaves of *Salix* *linearistipularis*.(data in Fig.5A)

# Table S14 Changes of K^+^ absorption and transport in roots and leaves of *Salix* *matsudana*.(data in Fig.5B)

# Table S15 Changes of K^+^ absorption and transport in roots and leaves of *Salix* *gordejevii*.(data in Fig.5C)

# Table S16 Changes of Ca^2+^ absorption and transport in roots and leaves of *Salix* *linearistipularis*.(data in Fig.6A)

# Table S17 Changes of Ca^2+^ absorption and transport in roots and leaves of *Salix* *matsudana*.(data in Fig.6B)

# Table S18 Changes of Ca^2+^ absorption and transport in roots and leaves of *Salix* *gordejevii*.(data in Fig.6C)

**Table S19** Changes of Na^+^/ K^+^ in roots and leaves of *Salix* *linearistipularis*.(data in Fig.7A)

**Table S20** Changes of Na^+^/ K^+^ in roots and leaves of *Salix* *matsudana*.(data in Fig.7B)

# Table S21 Changes of Na^+^/ K^+^ in roots and leaves of *Salix* *gordejevii*.(data in Fig.7C)

# Table S22 Changes of Na^+^/ Ca^2+^ in roots and leaves of *Salix* *linearistipularis*.(data in Fig.8A)

# Table S23 Changes of Na^+^/ Ca^2+^ in roots and leaves of *Salix* *matsudana*.(data in Fig.8B)

# Table S24 Changes of Na^+^/ Ca^2+^ in roots and leaves of *Salix* *gordejevii*.(data in Fig.8C)

# Table S25 Correlation of Na^+^in roots and leaves of three willows under salt stress.(data in Fig.9A)

# Table S26 Correlation of Na^+^/K^+^ in roots and leaves of three willows under salt stress.(data in Fig.9B)

# Table S27 Correlation of Na^+^/Ca^2+^ in roots and leaves of three willows under salt stress.(data in Fig.9C)

**Figure S1** Design of three willows experimental treatment.(data in Fig.1)

**Figure S2** Phenotypic response of *Salix linearistipularis* to salt stress.(data in Fig.2A)

**Figure S3** Phenotypic response of *Salix matsudana* to salt stress.(data in Fig.2B)

**Figure S4** Phenotypic response of *Salix gordejevii* to salt stress.(data in Fig.2C)

**Figure S5** Effects of salt stress on Na^+^, Ca^2+^ and K^+^ contents in roots and leaves of *Salix* *linearistipularis*.(data in Fig.3A)

**Figure S6** Effects of salt stress on Na^+^, Ca^2+^ and K^+^ contents in roots and leaves of *Salix* *matsudana*.(data in Fig.3B)

**Figure S7** Effects of salt stress on Na^+^, Ca^2+^ and K^+^ contents in roots and leaves of *Salix* *gordejevii*.(data in Fig.3C)

**Figure S8** Ratio of Na^+^ to CK in roots/leaves of *Salix* *linearistipularis*.(data in Fig.4A)

**Figure S9** Ratio of Na^+^ to CK in roots/leaves of *Salix* *matsudana*.(data in Fig.4B)

**Figure S10** Ratio of Na^+^ to CK in roots/leaves of *Salix* *gordejevii*.(data in Fig.4C)

**Figure S11** Ratio of K^+^ to CK in roots/leaves of *Salix* *linearistipularis*.(data in Fig.4D)

**Figure S12** Ratio of K^+^ to CK in roots/leaves of *Salix* *matsudana*.(data in Fig.4E)

**Figure S13** Ratio of K^+^ to CK in roots/leaves of *Salix* *gordejevii*.(data in Fig.4F)

**Figure S14** Ratio of Ca^2+^ to CK in roots/leaves of *Salix* *linearistipularis*.(data in Fig.4G)

**Figure S15** Ratio of Ca^2+^ to CK in roots/leaves of *Salix* *matsudana*.(data in Fig.4H)

**Figure S16** Ratio of Ca^2+^ to CK in roots/leaves of *Salix* *gordejevii*.(data in Fig.4I)

**Figure S17** Changes of K^+^ absorption and transport in roots and leaves of *Salix* *linearistipularis*.(data in Fig.5A)

**Figure S18** Changes of K^+^ absorption and transport in roots and leaves of *Salix* *matsudana*.(data in Fig.5B)

**Figure S19** Changes of K^+^ absorption and transport in roots and leaves of *Salix* *gordejevii*.(data in Fig.5C)

**Figure S20** Changes of Ca^2+^ absorption and transport in roots and leaves of *Salix* *linearistipularis*.(data in Fig.6A)

**Figure S21** Changes of Ca^2+^ absorption and transport in roots and leaves of *Salix* *matsudana*.(data in Fig.6B)

**Figure S22** Changes of Ca^2+^ absorption and transport in roots and leaves of *Salix* *gordejevii*.(data in Fig.6C)

**Figure S23** Changes of Na^+^/ K^+^ in roots and leaves of *Salix* *linearistipularis*.(data in Fig.7A)

**Figure S24** Changes of Na^+^/ K^+^ in roots and leaves of *Salix* *matsudana*.(data in Fig.7B)

**Figure S25** Changes of Na^+^/ K^+^ in roots and leaves of *Salix* *gordejevii*.(data in Fig.7C)

**Figure S26** Changes of Na^+^/ Ca^2+^ in roots and leaves of *Salix* *linearistipularis*.(data in Fig.8A)

**Figure S27** Changes of Na^+^/ Ca^2+^ in roots and leaves of *Salix* *matsudana*.(data in Fig.8B)

**Figure S28** Changes of Na^+^/ Ca^2+^ in roots and leaves of *Salix* *gordejevii*.(data in Fig.8C)

**Figure S29** Correlation of Na^+^in roots and leaves of three willows under salt stress.(data in Fig.9A)

**Figure S30** Correlation of Na^+^/K^+^ in roots and leaves of three willows under salt stress.(data in Fig.9B)

**Figure S31** Correlation of Na^+^/Ca^2+^ in roots and leaves of three willows under salt stress.(data in Fig.9C)

**Table S1** Effects of salt stress on Na^+^, Ca^2+^ and K^+^ contents in roots and leaves of *Salix linearistipularis*.(data in Fig.3A)

| indicators | NaCl Concentration(mM) | NaCl stress treatment time(Days) | | | | | |
| --- | --- | --- | --- | --- | --- | --- | --- |
|  |  | 1 | 3 | 5 | 8 | 11 | 15 |
| Na+ content in leaves(mg/g) | CK | 1.436±0.027e | 1.448±0.069e | 1.454±0.081e | 1.481±0.060e | 1.453±0.044e | 1.442±0.034c |
|  | 171 | 2.304±0.258d | 2.438±0.194d | 4.077±0.176d | 5.374±0.231d | 6.647±0.297d | 8.555±0.378b |
|  | 342 | 3.920±0.116c | 4.621±0.170c | 7.143±0.202c | 8.347±0.303c | 11.268±0.539c | 13.593±0.322a |
|  | 513 | 6.486±0.300b | 8.300±0.228b | 10.169±0.747b | 12.477±0.551b | 15.799±0.538b |  |
|  | 684 | 8.489±0.165a | 10.877±0.608a | 13.905±1.177a | 14.227±1.307a | 18.750±0.890a |  |
| Na+ content in roots(mg/g) | CK | 3.289±0.146e | 3.444±0.165e | 3.441±0.126e | 3.475±0.239d | 3.496±0.194e | 3.443±0.045c |
|  | 171 | 5.533±0.345d | 7.271±0.330d | 8.375±0.329d | 9.838±0.578c | 11.953±0.623d | 11.959±0.235b |
|  | 342 | 8.789±0.537c | 10.308±0.521c | 12.518±0.735c | 14.695±0.648b | 15.716±0.407c | 16.367±0.926a |
|  | 513 | 10.227±0.468b | 11.836±0.747b | 14.882±0.459b | 15.835±0.916ab | 17.972±0.636b |  |
|  | 684 | 12.328±0.518a | 14.487±0.709a | 16.093±0.611a | 17.491±0.775a | 19.443±0.850a |  |
| K+ content in leaves(mg/g) | CK | 24.351±0.983a | 23.506±1.341a | 24.070±0.828a | 24.499±1.288a | 24.169±1.079a | 24.462±1.341a |
|  | 171 | 24.280±1.548a | 23.730±1.309a | 22.713±0.238b | 20.812±0.878b | 16.074±0.905b | 10.919±0.230b |
|  | 342 | 24.853±0.864a | 19.097±0.752b | 17.367±0.960c | 14.012±0.884c | 10.151±0.282c | 6.495±0.255c |
|  | 513 | 23.172±0.461b | 17.879±0.171c | 14.893±0.805d | 11.472±0.399d | 6.599±0.320d |  |
|  | 684 | 16.684±0.717c | 15.629±0.848d | 13.165±0.766e | 7.407±0.278e | 5.112±0.174e |  |
| K+ content in roots(mg/g) | CK | 14.543±0.835a | 14.607±0.257a | 14.569±0.286a | 14.257±1.054a | 14.442±0.836a | 14.405±0.340a |
|  | 171 | 12.385±0.743b | 12.694±0.284b | 11.340±0.859b | 10.530±0.699b | 8.602±0.394b | 4.596±0.240b |
|  | 342 | 10.752±0.724c | 9.656±0.391c | 7.408±0.262c | 6.858±0.245c | 5.187±0.166c | 2.929±0.189c |
|  | 513 | 9.416±0.242d | 6.658±0.547d | 5.723±0.219d | 4.455±0.231d | 2.823±0.333d |  |
|  | 684 | 6.309±0.313e | 5.348±0.330e | 4.274±0.258e | 2.798±0.148e | 1.981±0.048e |  |
| Ca2+ content in leaves(mg/g) | CK | 3.178±0.127a | 3.167±0.055a | 3.101±0.173a | 3.055±0.197a | 3.099±0.093a | 3.146±0.028a |
|  | 171 | 2.992±0.141ab | 3.045±0.160ab | 2.766±0.155b | 2.519±0.087b | 1.743±0.080b | 1.425±0.049b |
|  | 342 | 2.952±0.099ab | 2.965±0.122b | 2.481±0.110c | 1.937±0.150c | 1.216±0.047c | 0.833±0.034c |
|  | 513 | 2.955±0.158ab | 2.573±0.067c | 1.913±0.097d | 1.507±0.053d | 0.979±0.058d |  |
|  | 684 | 2.858±0.135b | 2.439±0.067c | 1.685±0.048e | 1.269±0.061e | 0.675±0.027e |  |
| Ca2+ content in roots(mg/g) | CK | 1.771±0.005a | 1.745±0.086a | 1.734±0.099a | 1.690±0.097a | 1.738±0.111a | 1.735±0.089a |
|  | 171 | 1.675±0.066b | 1.620±0.034b | 1.489±0.063b | 1.347±0.118b | 0.947±0.077b | 0.630±0.038b |
|  | 342 | 1.652±0.044b | 1.483±0.067c | 1.124±0.031c | 0.982±0.026c | 0.547±0.019c | 0.334±0.011c |
|  | 513 | 1.541±0.057c | 1.220±0.059d | 0.863±0.035d | 0.654±0.028d | 0.397±0.029d |  |
|  | 684 | 1.343±0.060d | 1.079±0.043e | 0.632±0.038e | 0.556±0.016e | 0.264±0.022e |  |
| Note: the values displayed are means±SE.; different letters within a variable are significantly different at P < 0.05 | | | | | | | |

**Table S2** Effects of salt stress on Na^+^, Ca^2+^ and K^+^ contents in roots and leaves of *Salix* *matsudana*.(data in Fig.3B)

| indicators | NaCl Concentration(mM) | NaCl stress treatment time(Days) | | | | | |
| --- | --- | --- | --- | --- | --- | --- | --- |
|  |  | 1 | 3 | 5 | 8 | 11 | 15 |
| Na+ content in leaves(mg/g) | CK | 4.052±0.159e | 4.038±0.118e | 4.045±0.020e | 4.076±0.209e | 4.161±0.147e | 4.246±0.118d |
|  | 171 | 6.206±0.132d | 7.634±0.459d | 8.586±0.184d | 12.052±0.780d | 14.885±0.918d | 18.795±0.906c |
|  | 342 | 8.920±0.499c | 9.807±0.630c | 13.942±0.684c | 21.049±0.833c | 26.066±0.689c | 33.184±2.790b |
|  | 513 | 11.229±0.449b | 15.028±1.293b | 21.411±0.881b | 28.842±0.931b | 34.227±1.420b | 39.972±3.709a |
|  | 684 | 14.500±1.450a | 24.655±0.973a | 31.249±0.778a | 43.365±2.276a | 54.004±2.312a |  |
| Na+ content in roots(mg/g) | CK | 7.541±0.045e | 7.615±0.009e | 7.629±0.032e | 7.750±0.057e | 7.839±0.062e | 7.989±0.067d |
|  | 171 | 13.013±0.760d | 14.841±0.580d | 18.654±1.675d | 23.084±2.851d | 23.716±0.437d | 25.766±0.880c |
|  | 342 | 20.312±0.868c | 22.096±2.061c | 26.367±1.512c | 30.073±1.690c | 31.080±0.935c | 33.248±1.120b |
|  | 513 | 24.502±1.204b | 26.759±1.292b | 30.011±1.354b | 33.713±0.859b | 34.491±1.423b | 37.588±2.227a |
|  | 684 | 28.690±0.434a | 34.796±0.851a | 36.985±2.364a | 42.423±1.015a | 56.461±2.671a |  |
| K+ content in leaves(mg/g) | CK | 39.386±1.441a | 37.889±0.950a | 38.617±1.592a | 37.867±1.652a | 37.821±0.891a | 38.308±0.575a |
|  | 171 | 40.440±2.015a | 37.307±0.741a | 37.073±1.794a | 27.560±0.529b | 22.399±1.010b | 20.579±0.943b |
|  | 342 | 28.320±2.303b | 26.127±0.919b | 23.838±0.865b | 19.250±0.898c | 12.917±0.079c | 10.520±0.765c |
|  | 513 | 26.433±0.831b | 24.314±0.499c | 20.548±0.708c | 13.738±0.493d | 9.574±0.356d | 8.491±0.316d |
|  | 684 | 26.030±0.714b | 21.313±1.595d | 15.547±0.874d | 10.049±0.703e | 6.729±0.153e |  |
| K+ content in roots(mg/g) | CK | 35.527±1.491a | 36.675±2.881a | 35.540±0.975a | 36.185±1.431a | 36.610±0.584a | 37.120±2.452a |
|  | 171 | 35.620±1.572a | 36.501±1.001a | 35.949±1.204a | 27.453±1.194b | 20.977±0.725b | 18.558±0.732b |
|  | 342 | 31.360±0.803b | 27.700±0.531b | 22.620±0.720b | 18.635±0.618c | 11.661±0.640c | 9.795±0.470c |
|  | 513 | 28.680±1.793c | 24.621±1.000c | 16.853±0.903c | 11.292±0.535d | 7.862±0.231d | 7.347±0.054d |
|  | 684 | 26.162±0.741d | 22.903±0.633d | 14.087±0.990d | 9.397±0.222e | 6.586±0.231e |  |
| Ca2+ content in leaves(mg/g) | CK | 5.552±0.220a | 5.508±0.170a | 5.502±0.132a | 5.472±0.242a | 5.526±0.163a | 5.511±0.292a |
|  | 171 | 5.510±0.049a | 5.275±0.122a | 4.380±0.270b | 3.947±0.093b | 3.571±0.166b | 3.013±0.197b |
|  | 342 | 5.277±0.208ab | 3.577±0.110b | 3.494±0.050c | 3.238±0.076c | 2.851±0.081c | 2.313±0.048c |
|  | 513 | 5.172±0.013b | 3.296±0.066c | 3.213±0.057d | 2.780±0.145d | 2.469±0.125d | 1.547±0.091d |
|  | 684 | 4.771±0.116c | 3.051±0.149d | 3.058±0.062e | 2.596±0.031d | 1.723±0.017e |  |
| Ca2+ content in roots(mg/g) | CK | 2.681±0.075a | 2.581±0.086a | 2.684±0.085a | 2.645±0.166a | 2.675±0.069a | 2.468±0.107a |
|  | 171 | 2.607±0.069a | 2.598±0.072a | 2.485±0.088b | 1.955±0.082b | 1.552±0.087b | 1.142±0.057b |
|  | 342 | 2.634±0.087a | 2.048±0.165b | 1.741±0.029c | 1.211±0.040c | 0.932±0.035c | 0.643±0.016c |
|  | 513 | 2.105±0.183b | 1.432±0.059c | 1.223±0.024d | 0.867±0.034d | 0.681±0.027d | 0.466±0.022d |
|  | 684 | 1.757±0.098c | 1.245±0.061d | 1.074±0.018e | 0.784±0.023e | 0.557±0.042e |  |
| Note: the values displayed are means±SE.; different letters within a variable are significantly different at P < 0.05 | | | | | | | |

**Table S3** Effects of salt stress on Na^+^, Ca^2+^ and K^+^ contents in roots and leaves of *Salix* *gordejevii*.(data in Fig.3C)

| indicators | NaCl Concentration(mM) | NaCl stress treatment time(Days) | | | | | |
| --- | --- | --- | --- | --- | --- | --- | --- |
|  |  | 1 | 3 | 5 | 8 | 11 | 15 |
| Na^+^ content in leaves(mg/g) | CK | 3.431±0.244e | 3.541±0.278e | 3.486±0.124e | 3.525±0.167e | 3.625±0.051e | 3.618±0.016e |
|  | 171 | 5.406±0.240d | 5.458±0.252d | 7.433±0.341d | 9.726±0.350d | 11.986±0.376d | 15.800±0.846d |
|  | 342 | 7.929±0.250c | 9.188±0.625c | 15.861±0.522c | 19.301±0.716c | 22.058±0.745c | 28.675±0.989c |
|  | 513 | 9.206±0.684b | 12.530±0.535b | 21.687±0.286b | 23.485±0.412b | 33.904±1.767b | 38.348±2.425b |
|  | 684 | 11.015±0.622a | 15.698±0.495a | 26.334±0.855a | 29.369±1.262a | 37.206±1.556a | 41.202±1.996a |
| Na^+^ content in roots(mg/g) | CK | 6.676±0.026e | 6.703±0.406e | 6.658±0.130e | 6.589±0.323e | 6.896±0.232e | 6.765±0.404e |
|  | 171 | 11.525±0.465d | 13.675±0.499d | 16.015±0.711d | 18.560±0.867d | 21.006±1.408d | 23.589±1.544d |
|  | 342 | 17.792±0.870c | 20.257±0.757c | 24.499±1.375c | 27.982±0.743c | 29.055±2.182c | 32.238±0.878c |
|  | 513 | 20.080±1.218b | 23.006±1.313b | 28.255±0.920b | 33.683±1.465b | 35.939±1.633b | 38.638±1.417b |
|  | 684 | 23.701±1.167a | 28.004±0.818a | 31.911±1.589a | 35.399±1.587a | 41.748±1.680a | 44.506±2.544a |
| K^+^ content in leaves(mg/g) | CK | 27.870±0.672a | 28.529±0.983a | 27.793±1.683a | 27.407±0.518a | 27.713±0.962a | 27.733±1.336a |
|  | 171 | 27.553±1.147a | 25.116±1.011b | 24.502±0.612b | 22.483±0.724b | 20.475±0.886b | 16.980±0.650b |
|  | 342 | 21.713±0.907b | 21.111±0.989c | 20.660±0.893c | 15.754±0.862c | 12.337±0.415c | 9.547±0.410c |
|  | 513 | 22.151±0.633b | 19.052±0.854d | 17.557±0.422d | 12.015±0.394d | 9.929±0.392d | 6.132±0.277d |
|  | 684 | 22.707±0.988b | 19.651±0.603cd | 15.748±0.849e | 9.214±0.965e | 6.670±0.410e | 4.659±0.043e |
| K^+^ content in roots(mg/g) | CK | 23.236±0.733a | 23.400±1.327a | 23.380±1.322a | 23.180±0.927a | 23.104±0.506a | 23.208±1.246a |
|  | 171 | 22.627±0.591a | 23.833±1.371a | 22.420±1.703a | 19.976±0.696b | 17.490±1.268b | 16.893±0.600b |
|  | 342 | 20.495±0.780b | 19.777±0.513b | 17.477±0.652b | 14.825±0.830c | 12.008±0.338c | 9.481±0.412c |
|  | 513 | 19.167±0.420c | 17.255±0.635c | 13.577±0.379c | 11.256±0.359d | 8.475±0.520d | 6.930±0.416d |
|  | 684 | 18.079±0.908c | 15.806±0.845c | 11.257±0.440d | 7.932±0.367e | 7.041±0.173e | 5.827±0.239e |
| Ca^2+^ content in leaves(mg/g) | CK | 2.326±0.072b | 2.312±0.134a | 2.301±0.101a | 2.296±0.130a | 2.262±0.138a | 2.245±0.083a |
|  | 171 | 2.375±0.080b | 2.347±0.066a | 2.097±0.106a | 1.685±0.064b | 1.434±0.088b | 1.251±0.071b |
|  | 342 | 2.586±0.124a | 2.086±0.072b | 1.909±0.058b | 1.450±0.074c | 1.130±0.006c | 1.084±0.008c |
|  | 513 | 2.343±0.077b | 1.972±0.088b | 1.801±0.047c | 1.290±0.035d | 0.964±0.045d | 0.750±0.034d |
|  | 684 | 2.357±0.060b | 1.778±0.032c | 1.584±0.029d | 1.157±0.010e | 0.732±0.016e | 0.470±0.021e |
| Ca^2+^ content in roots(mg/g) | CK | 1.578±0.088a | 1.467±0.097a | 1.511±0.084a | 1.450±0.037a | 1.489±0.082a | 1.490±0.117a |
|  | 171 | 1.539±0.003ab | 1.455±0.011ab | 1.422±0.036b | 1.343±0.057b | 1.126±0.030b | 0.960±0.056b |
|  | 342 | 1.456±0.088bc | 1.371±0.044bc | 1.146±0.036c | 1.031±0.125c | 0.708±0.095c | 0.612±0.007c |
|  | 513 | 1.443±0.034bc | 1.285±0.006c | 0.949±0.049d | 0.874±0.026d | 0.560±0.021d | 0.446±0.016d |
|  | 684 | 1.348±0.022c | 1.139±0.011d | 0.769±0.035e | 0.662±0.035e | 0.467±0.026e | 0.344±0.017e |
| Note: the values displayed are means±SE.; different letters within a variable are significantly different at P < 0.05 | | | | | | | |

**Table S4** Ratio of Na^+^ to CK in roots/leaves of *Salix* *linearistipularis*.(data in Fig.4A)

| indicators | NaCl Concentration(mM) | NaCl stress treatment time(Days) | | | | | |
| --- | --- | --- | --- | --- | --- | --- | --- |
|  |  | 1 | 3 | 5 | 8 | 11 | 15 |
| Ratio of Na^+^ in root and leaf /CK group | CK | 1.000±0.000a | 1.000±0.000b | 1.000±0.000a | 1.000±0.000a | 1.000±0.000a | 1.000±0.000a |
|  | 171 | 1.060±0.083a | 1.267±0.096a | 0.870±0.015b | 0.783±0.026b | 0.747±0.033b | 0.590±0.021b |
|  | 342 | 0.980±0.015a | 0.943±0.049b | 0.743±0.028c | 0.753±0.034b | 0.580±0.010c | 0.503±0.012c |
|  | 513 | 0.690±0.020b | 0.600±0.031c | 0.620±0.010d | 0.543±0.035c | 0.473±0.015d | 0.430±0.012d |
|  | 684 | 0.633±0.012b | 0.560±0.029c | 0.490±0.020e | 0.527±0.019c | 0.430±0.015d |  |

**Table S5** Ratio of Na^+^ to CK in roots/leaves of *Salix* *matsudana*.(data in Fig.4B)

| indicators | NaCl Concentration(mM) | NaCl stress treatment time(Days) | | | | | |
| --- | --- | --- | --- | --- | --- | --- | --- |
|  |  | 1 | 3 | 5 | 8 | 11 | 15 |
| Ratio of Na^+^ in root and leaf /CK group | CK | 1.000±0.000c | 1.000±0.000b | 1.000±0.000b | 1.000±0.000a | 1.000±0.000a | 1.000±0.000a |
|  | 171 | 1.127±0.035abc | 1.030±0.020b | 1.150±0.065a | 1.010±0.059a | 0.847±0.032b | 0.727±0.003b |
|  | 342 | 1.227±0.057a | 1.193±0.009a | 1.007±0.037b | 0.750±0.020b | 0.633±0.032c | 0.530±0.020c |
|  | 513 | 1.177±0.055ab | 0.950±0.050b | 0.743±0.018c | 0.617±0.019c | 0.537±0.003d | 0.503±0.032c |
|  | 684 | 1.063±0.015bc | 0.750±0.010c | 0.627±0.013d | 0.517±0.020d | 0.553±0.026d |  |

**Table S6** Ratio of Na^+^ to CK in roots/leaves of *Salix* *gordejevii*.(data in Fig.4C)

| indicators | NaCl Concentration(mM) | NaCl stress treatment time(Days) | | | | | |
| --- | --- | --- | --- | --- | --- | --- | --- |
|  |  | 1 | 3 | 5 | 8 | 11 | 15 |
| Ratio of Na^+^ in root and leaf /CK group | CK | 1.000±0.000a | 1.000±0.000b | 1.000±0.000b | 1.000±0.000a | 1.000±0.000a | 1.000±0.000a |
|  | 171 | 1.097±0.003a | 1.327±0.067a | 1.130±0.000a | 1.020±0.000a | 0.920±0.021b | 0.800±0.038b |
|  | 342 | 1.153±0.071a | 1.180±0.121ab | 0.813±0.033c | 0.777±0.035b | 0.690±0.010c | 0.600±0.006c |
|  | 513 | 1.123±0.039a | 0.977±0.093b | 0.683±0.012d | 0.767±0.012b | 0.560±0.021d | 0.540±0.012c |
|  | 684 | 1.110±0.067a | 0.947±0.050b | 0.633±0.023d | 0.647±0.026c | 0.593±0.018d | 0.577±0.012c |

**Table S7** Ratio of K^+^ to CK in roots/leaves of *Salix* *linearistipularis*.(data in Fig.4D)

| indicators | NaCl Concentration(mM) | NaCl stress treatment time(Days) | | | | | |
| --- | --- | --- | --- | --- | --- | --- | --- |
|  |  | 1 | 3 | 5 | 8 | 11 | 15 |
| Ratio of K^+^ in root and leaf /CK group | CK | 1.000±0.000a | 1.000±0.000a | 1.000±0.000a | 1.000±0.000a | 1.000±0.000a | 1.000±0.000a |
|  | 171 | 0.857±0.045b | 0.863±0.035b | 0.823±0.022b | 0.877±0.074ab | 0.900±0.046a | 0.713±0.023b |
|  | 342 | 0.723±0.037c | 0.813±0.024b | 0.707±0.009c | 0.847±0.054b | 0.863±0.081ab | 0.767±0.057b |
|  | 513 | 0.680±0.012c | 0.597±0.013c | 0.633±0.013d | 0.670±0.044c | 0.720±0.057bc | 0.683±0.015b |
|  | 684 | 0.633±0.037c | 0.550±0.038c | 0.537±0.030e | 0.650±0.017c | 0.650±0.032c |  |

**Table S8** Ratio of K^+^ to CK in roots/leaves of *Salix* *matsudana*.(data in Fig.4E)

| indicators | NaCl Concentration(mM) | NaCl stress treatment time(Days) | | | | | |
| --- | --- | --- | --- | --- | --- | --- | --- |
|  |  | 1 | 3 | 5 | 8 | 11 | 15 |
| Ratio of K^+^ in root and leaf /CK group | CK | 1.000±0.000c | 1.000±0.000b | 1.000±0.000b | 1.000±0.000a | 1.000±0.000a | 1.000±0.000a |
|  | 171 | 0.977±0.027c | 0.983±0.023b | 1.153±0.012a | 1.043±0.023a | 0.967±0.017ab | 0.933±0.032ab |
|  | 342 | 1.227±0.007a | 1.093±0.018a | 1.033±0.037b | 1.017±0.041a | 0.930±0.020b | 0.963±0.009ab |
|  | 513 | 1.203±0.027a | 1.047±0.023ab | 0.890±0.038c | 1.013±0.023a | 0.850±0.015c | 0.893±0.026b |
|  | 684 | 1.113±0.039b | 1.100±0.031a | 0.983±0.015b | 0.977±0.023a | 1.013±0.020a |  |

**Table S9** Ratio of K^+^ to CK in roots/leaves of *Salix* *gordejevii*.(data in Fig.4F)

| indicators | NaCl Concentration(mM) | NaCl stress treatment time(Days) | | | | | |
| --- | --- | --- | --- | --- | --- | --- | --- |
|  |  | 1 | 3 | 5 | 8 | 11 | 15 |
| Ratio of K^+^ in root and leaf /CK group | CK | 1.000±0.000bc | 1.000±0.000b | 1.000±0.000b | 1.000±0.000c | 1.000±0.000c | 1.000±0.000c |
|  | 171 | 1.067±0.030ab | 1.260±0.070a | 1.183±0.023a | 1.153±0.039a | 1.140±0.071abc | 1.347±0.013ab |
|  | 342 | 1.133±0.058a | 1.147±0.063ab | 1.007±0.009b | 1.113±0.013ab | 1.170±0.031ab | 1.190±0.025b |
|  | 513 | 1.040±0.021abc | 1.107±0.043ab | 0.920±0.015bc | 1.110±0.031ab | 1.027±0.019bc | 1.347±0.041ab |
|  | 684 | 0.957±0.003c | 1.160±0.026ab | 0.853±0.052c | 1.023±0.035bc | 1.270±0.068a | 1.513±0.116a |

**Table S10** Ratio of Ca^2+^ to CK in roots/leaves of *Salix* *linearistipularis*.(data in Fig.4G)

| indicators | NaCl Concentration(mM) | NaCl stress treatment time(Days) | | | | | |
| --- | --- | --- | --- | --- | --- | --- | --- |
|  |  | 1 | 3 | 5 | 8 | 11 | 15 |
| Ratio of Ca2+ in root and leaf /CK group | CK | 1.000±0.000a | 1.000±0.000a | 1.000±0.000a | 1.000±0.000a | 1.000±0.000a | 1.000±0.000a |
|  | 171 | 1.003±0.024a | 0.973±0.069a | 0.963±0.033a | 0.967±0.032a | 0.970±0.061a | 0.800±0.015b |
|  | 342 | 1.003±0.032a | 0.913±0.063ab | 0.813±0.041b | 0.917±0.029a | 0.803±0.015b | 0.730±0.015c |
|  | 513 | 0.937±0.007a | 0.860±0.021ab | 0.810±0.023b | 0.787±0.044b | 0.723±0.041b | 0.720±0.036c |
|  | 684 | 0.843±0.027b | 0.803±0.027c | 0.67±0.006c | 0.790±0.035b | 0.697±0.043b |  |

**Table S11** Ratio of Ca^2+^ to CK in roots/leaves of *Salix* *matsudana*.(data in Fig.4H)

| indicators | NaCl Concentration(mM) | NaCl stress treatment time(Days) | | | | | |
| --- | --- | --- | --- | --- | --- | --- | --- |
|  |  | 1 | 3 | 5 | 8 | 11 | 15 |
| Ratio of Ca^2+^ in root and leaf /CK group | CK | 1.000±0.000a | 1.000±0.000bc | 1.000±0.000b | 1.000±0.000a | 1.000±0.000a | 1.000±0.000a |
|  | 171 | 0.980±0.006a | 1.050±0.021b | 1.163±0.032a | 1.023±0.019a | 0.897±0.023b | 0.847±0.030b |
|  | 342 | 1.037±0.026a | 1.223±0.034a | 1.023±0.017b | 0.773±0.018b | 0.677±0.012c | 0.620±0.006c |
|  | 513 | 0.840±0.030b | 0.930±0.035cd | 0.780±0.015c | 0.647±0.018c | 0.570±0.015c | 0.673±0.032c |
|  | 684 | 0.760±0.025c | 0.867±0.015d | 0.720±0.012d | 0.623±0.009c | 0.670±0.038d |  |

**Table S12** Ratio of Ca^2+^ to CK in roots/leaves of *Salix* *gordejevii*.(data in Fig.4I)

| indicators | NaCl Concentration(mM) | NaCl stress treatment time(Days) | | | | | |
| --- | --- | --- | --- | --- | --- | --- | --- |
|  |  | 1 | 3 | 5 | 8 | 11 | 15 |
| Ratio of Ca^2+^ in root and leaf /CK group | CK | 1.000±0.000a | 1.000±0.000a | 1.000±0.000ab | 1.000±0.000bc | 1.000±0.000b | 1.000±0.000b |
|  | 171 | 0.953±0.023ab | 0.980±0.042a | 1.037±0.049a | 1.263±0.042a | 1.193±0.019a | 1.157±0.009a |
|  | 342 | 0.830±0.032c | 1.040±0.050a | 0.913±0.034b | 1.130±0.055b | 0.950±0.072b | 0.850±0.017c |
|  | 513 | 0.910±0.015b | 1.030±0.042a | 0.807±0.033c | 1.073±0.026b | 0.883±0.009b | 0.897±0.007c |
|  | 684 | 0.843±0.009c | 1.013±0.047a | 0.740±0.010c | 0.907±0.048c | 0.967±0.030b | 1.107±0.058a |

**Table S13** Changes of K^+^ absorption and transport in roots and leaves of *Salix* *linearistipularis*.(data in Fig.5A)

| indicators | NaCl Concentration(mM) | NaCl stress treatment time(Days) | | | | | |
| --- | --- | --- | --- | --- | --- | --- | --- |
|  |  | 1 | 3 | 5 | 8 | 11 | 15 |
| SA_K, Na_ of roots | CK | ---- | ---- | ---- | ---- | ---- | ---- |
|  | 171 | 3.251±0.387bc | 2.523±0.019ab | 1.956±0.135a | 1.548±0.080a | 1.043±0.089a | 0.555±0.021a |
|  | 342 | 3.534±0.207b | 2.711±0.142a | 1.710±0.029b | 1.349±0.050b | 0.954±0.042b | 0.518±0.047a |
|  | 513 | 3.986±0.045a | 2.434±0.170b | 1.665±0.076b | 1.219±0.046c | 0.680±0.010c |  |
|  | 684 | 2.957±0.119c | 2.133±0.102c | 1.538±0.136b | 0.925±0.049d | 0.589±0.007d |  |
| ST_K, Na_ of leaves | CK | 3.838±0.108b | 3.825±0.240b | 3.909±0.115a | 4.039±0.212a | 4.035±0.307a | 4.057±0.070a |
|  | 171 | 4.817±0.254a | 5.597±0.404a | 4.125±0.198a | 3.636±0.211ab | 3.375±0.228b | 3.334±0.189b |
|  | 342 | 5.200±0.358a | 4.420±0.348b | 4.110±0.116a | 3.604±0.156b | 2.734±0.178c | 2.684±0.108c |
|  | 513 | 3.887±0.217b | 3.846±0.191b | 3.812±0.155ab | 3.271±0.131c | 2.658±0.059c |  |
|  | 684 | 3.849±0.251b | 3.905±0.284b | 3.591±0.148b | 3.266±0.116c | 2.677±0.132c |  |
| Note: the values displayed are means±SE.; different letters within a variable are significantly different at P < 0.05 | | | | | | | |

**Table S14** Changes of K^+^ absorption and transport in roots and leaves of *Salix* *matsudana*.(data in Fig.5B)

| indicators | NaCl Concentration(mM) | NaCl stress treatment time(Days) | | | | | |
| --- | --- | --- | --- | --- | --- | --- | --- |
|  |  | 1 | 3 | 5 | 8 | 11 | 15 |
| SA_K, Na_ of roots | CK | ---- | ---- | ---- | ---- | ---- | ---- |
|  | 171 | 3.956±0.036c | 3.556±0.160b | 2.799±0.231a | 1.718±0.012b | 1.278±0.009a | 1.041±0.023a |
|  | 342 | 4.463±0.140b | 3.623±0.045b | 2.481±0.112ab | 1.792±0.064a | 1.085±0.035b | 0.852±0.037b |
|  | 513 | 5.068±0.227a | 3.984±0.168a | 2.431±0.048b | 1.450±0.049c | 0.987±0.037c | 0.846±0.006b |
|  | 684 | 5.273±0.214a | 3.805±0.032ab | 2.204±0.183b | 1.281±0.041d | 0.675±0.016d |  |
| ST_K, Na_ of leaves | CK | 2.066±0.133b | 1.949±0.079b | 2.050±0.065a | 1.990±0.056a | 1.948±0.089a | 1.942±0.053a |
|  | 171 | 2.382±0.091a | 1.990±0.046b | 2.238±0.150a | 1.928±0.107a | 1.705±0.139b | 1.522±0.096b |
|  | 342 | 2.060±0.083b | 2.125±0.047a | 2.003±0.230a | 1.480±0.135b | 1.322±0.071c | 1.077±0.042c |
|  | 513 | 2.015±0.135b | 1.768±0.163c | 1.714±0.159b | 1.423±0.020b | 1.230±0.098cd | 1.094±0.125c |
|  | 684 | 1.972±0.129b | 1.327±0.057d | 1.308±0.066c | 1.048±0.092c | 1.069±0.069d |  |
| Note: the values displayed are means±SE.; different letters within a variable are significantly different at P < 0.05 | | | | | | | |

**Table S15** Changes of K^+^ absorption and transport in roots and leaves of *Salix* *gordejevii*.(data in Fig.5C)

| indicators | NaCl Concentration(mM) | NaCl stress treatment time(Days) | | | | | |
| --- | --- | --- | --- | --- | --- | --- | --- |
|  |  | 1 | 3 | 5 | 8 | 11 | 15 |
| SA_K, Na_ of roots | CK | ---- | ---- | ---- | ---- | ---- | ---- |
|  | 171 | 2.840±0.139d | 2.517±0.053c | 2.023±0.041a | 1.556±0.073a | 1.205±0.076a | 1.035±0.009a |
|  | 342 | 3.330±0.084c | 2.822±0.029b | 2.062±0.026a | 1.531±0.088a | 1.195±0.037a | 0.850±0.028b |
|  | 513 | 4.132±0.051b | 3.246±0.095a | 2.080±0.010a | 1.446±0.028a | 1.021±0.032b | 0.776±0.045c |
|  | 684 | 4.409±0.079a | 3.263±0.076a | 2.040±0.115a | 1.296±0.081b | 0.975±0.012b | 0.757±0.022c |
| ST_K, Na_ of leaves | CK | 2.341±0.151c | 2.316±0.198b | 2.271±0.066a | 2.211±0.075a | 2.282±0.040a | 2.234±0.018a |
|  | 171 | 2.597±0.110ab | 2.645±0.143a | 2.358±0.106a | 2.150±0.078a | 2.058±0.192b | 1.505±0.121b |
|  | 342 | 2.378±0.084bc | 2.357±0.088b | 1.827±0.116b | 1.544±0.091b | 1.354±0.020c | 1.133±0.047c |
|  | 513 | 2.527±0.118abc | 2.028±0.045c | 1.685±0.042b | 1.531±0.046b | 1.243±0.051c | 0.894±0.063d |
|  | 684 | 2.706±0.174a | 2.219±0.056bc | 1.696±0.082b | 1.397±0.031c | 1.064±0.066d | 0.862±0.090d |
| Note: the values displayed are means±SE.; different letters within a variable are significantly different at P < 0.05 | | | | | | | |

**Table S16** Changes of Ca^2+^ absorption and transport in roots and leaves of *Salix* *linearistipularis*.(data in Fig.6A)

| indicators | NaCl Concentration(mM) | NaCl stress treatment time(Days) | | | | | |
| --- | --- | --- | --- | --- | --- | --- | --- |
|  |  | 1 | 3 | 5 | 8 | 11 | 15 |
| SACa, Na of roots | CK | ---- | ---- | ---- | ---- | ---- | ---- |
|  | 171 | 0.519±0.051c | 0.380±0.014c | 0.303±0.003a | 0.234±0.002a | 0.135±0.009a | 0.090±0.007a |
|  | 342 | 0.642±0.011b | 0.492±0.019b | 0.307±0.004a | 0.228±0.006ab | 0.119±0.004b | 0.070±0.003b |
|  | 513 | 0.773±0.015a | 0.529±0.019a | 0.298±0.013a | 0.213±0.020b | 0.113±0.010b |  |
|  | 684 | 0.741±0.021a | 0.506±0.004ab | 0.268±0.025b | 0.216±0.003b | 0.092±0.007c |  |
| STCa, Na of leaves | CK | 4.110±0.155a | 4.320±0.261b | 4.237±0.235a | 4.241±0.289a | 4.289±0.132a | 4.332±0.175a |
|  | 171 | 4.369±0.235a | 5.617±0.183a | 3.816±0.093a | 3.428±0.168b | 3.323±0.185b | 3.173±0.207b |
|  | 342 | 4.017±0.261a | 4.464±0.359b | 3.869±0.232a | 3.477±0.181b | 3.104±0.145bc | 3.005±0.104b |
|  | 513 | 3.025±0.136b | 3.013±0.178c | 3.251±0.258b | 2.934±0.117c | 2.825±0.155cd |  |
|  | 684 | 3.092±0.175b | 3.013±0.107c | 3.097±0.259b | 2.806±0.019c | 2.668±0.195d |  |
| Note: the values displayed are means±SE.; different letters within a variable are significantly different at P < 0.05 | | | | | | | |

**Table S17** Changes of Ca^2+^ absorption and transport in roots and leaves of *Salix* *matsudana*.(data in Fig.6B)

| indicators | NaCl Concentration(mM) | NaCl stress treatment time(Days) | | | | | |
| --- | --- | --- | --- | --- | --- | --- | --- |
|  |  | 1 | 3 | 5 | 8 | 11 | 15 |
| SA_Ca, Na_ of roots | CK | ---- | ---- | ---- | ---- | ---- | ---- |
|  | 171 | 0.342±0.013b | 0.299±0.012a | 0.229±0.011a | 0.145±0.005a | 0.112±0.001a | 0.076±0.005a |
|  | 342 | 0.442±0.011a | 0.316±0.010a | 0.225±0.001a | 0.137±0.005a | 0.102±0.004b | 0.066±0.001b |
|  | 513 | 0.441±0.037a | 0.274±0.011b | 0.209±0.007b | 0.132±0.002b | 0.101±0.004b | 0.064±0.003b |
|  | 684 | 0.416±0.009a | 0.244±0.010c | 0.198±0.005b | 0.126±0.003c | 0.067±0.006c |  |
| ST_Ca, Na_ of leaves | CK | 3.856±0.159c | 4.028±0.211a | 3.866±0.049a | 3.932±0.044a | 3.902±0.314a | 4.206±0.275a |
|  | 171 | 4.434±0.133b | 3.956±0.233a | 3.830±0.326a | 3.885±0.426a | 3.667±0.082ab | 3.621±0.183b |
|  | 342 | 4.586±0.522b | 3.936±0.031a | 3.803±0.235a | 3.826±0.261a | 3.652±0.266ab | 3.606±0.205b |
|  | 513 | 5.385±0.370a | 4.124±0.404a | 3.685±0.109a | 3.753±0.120a | 3.663±0.195ab | 3.134±0.194c |
|  | 684 | 5.378±0.181a | 3.469±0.058b | 3.363±0.025b | 3.242±0.148b | 3.242±0.262b |  |
| Note: the values displayed are means±SE.; different letters within a variable are significantly different at P < 0.05 | | | | | | | |

**Table S18** Changes of Ca^2+^ absorption and transport in roots and leaves of *Salix* *gordejevii*.(data in Fig.6C)

| indicators | NaCl Concentration(mM) | NaCl stress treatment time(Days) | | | | | |
| --- | --- | --- | --- | --- | --- | --- | --- |
|  |  | 1 | 3 | 5 | 8 | 11 | 15 |
| SA_Ca, Na_ of roots | CK | ---- | ---- | ---- | ---- | ---- | ---- |
|  | 171 | 0.228±0.009c | 0.182±0.006d | 0.152±0.006c | 0.123±0.002b | 0.092±0.005a | 0.069±0.001a |
|  | 342 | 0.280±0.026b | 0.231±0.003c | 0.160±0.003b | 0.126±0.002b | 0.083±0.003b | 0.065±0.001b |
|  | 513 | 0.369±0.016a | 0.286±0.003a | 0.172±0.004a | 0.133±0.002a | 0.080±0.003b | 0.059±0.003c |
|  | 684 | 0.387±0.007a | 0.277±0.004b | 0.164±0.005b | 0.127±0.007ab | 0.076±0.005b | 0.053±0.003d |
| ST_Ca, Na_ of leaves | CK | 2.886±0.321c | 3.001±0.337bc | 2.911±0.075b | 2.961±0.083a | 2.890±0.018a | 2.818±0.077a |
|  | 171 | 3.297±0.246bc | 4.048±0.224a | 3.187±0.263a | 2.398±0.133b | 2.230±0.063b | 1.951±0.132b |
|  | 342 | 4.002±0.427a | 3.371±0.342b | 2.574±0.029c | 2.045±0.194c | 2.133±0.338bc | 1.994±0.034b |
|  | 513 | 3.552±0.242ab | 2.819±0.063c | 2.472±0.082c | 2.117±0.054c | 1.829±0.120cd | 1.695±0.019c |
|  | 684 | 3.766±0.163ab | 2.787±0.148c | 2.502±0.178c | 2.111±0.107c | 1.768±0.149d | 1.484±0.154d |
| Note: the values displayed are means±SE.; different letters within a variable are significantly different at P < 0.05 | | | | | | | |

**Table S19** Changes of Na^+^/ K^+^ in roots and leaves of *Salix* *linearistipularis*.(data in Fig.7A)

| indicators | NaCl Concentration(mM) | NaCl stress treatment time(Days) | | | | | |
| --- | --- | --- | --- | --- | --- | --- | --- |
|  |  | 1 | 3 | 5 | 8 | 11 | 15 |
| The ratio of Na^+^/ K^+^ in leaves | CK | 0.059±0.002e | 0.062±0.003e | 0.060±0.001e | 0.061±0.004e | 0.060±0.004e | 0.059±0.002c |
|  | 171 | 0.095±0.011d | 0.103±0.007d | 0.180±0.004d | 0.258±0.016d | 0.414±0.023d | 0.784±0.039b |
|  | 342 | 0.158±0.002c | 0.242±0.008c | 0.411±0.009c | 0.596±0.038c | 1.110±0.031c | 2.094±0.070a |
|  | 513 | 0.280±0.016b | 0.464±0.016b | 0.683±0.022b | 1.089±0.062b | 2.396±0.053b |  |
|  | 684 | 0.509±0.012a | 0.696±0.021a | 1.059±0.076a | 1.924±0.113a | 3.672±0.182a |  |
| The ratio of Na^+^/ K^+^ in roots | CK | 0.226±0.004e | 0.236±0.015e | 0.236±0.004e | 0.244±0.018e | 0.242±0.002e | 0.239±0.003c |
|  | 171 | 0.449±0.055d | 0.573±0.004d | 0.741±0.053d | 0.935±0.050d | 1.393±0.114d | 2.606±0.098b |
|  | 342 | 0.820±0.048c | 1.068±0.055c | 1.690±0.029c | 2.144±0.079c | 3.033±0.131c | 5.609±0.488a |
|  | 513 | 1.086±0.012b | 1.784±0.125b | 2.603±0.122b | 3.554±0.133b | 6.366±0.090b |  |
|  | 684 | 1.957±0.081a | 2.714±0.132a | 3.779±0.348a | 6.262±0.332a | 9.815±0.109a |  |
| Note: the values displayed are means±SE.; different letters within a variable are significantly different at P < 0.05 | | | | | | | |

**Table S20** Changes of Na^+^/ K^+^ in roots and leaves of *Salix* *matsudana*.(data in Fig.7B)

| indicators | NaCl Concentration(mM) | NaCl stress treatment time(Days) | | | | | |
| --- | --- | --- | --- | --- | --- | --- | --- |
|  |  | 1 | 3 | 5 | 8 | 11 | 15 |
| The ratio of Na^+^/ K^+^ in leaves | CK | 0.103±0.008e | 0.107±0.002e | 0.105±0.002e | 0.108±0.002e | 0.110±0.006e | 0.111±0.005d |
|  | 171 | 0.154±0.007d | 0.204±0.009d | 0.232±0.017d | 0.437±0.028d | 0.666±0.054d | 0.915±0.062c |
|  | 342 | 0.315±0.019c | 0.375±0.006c | 0.585±0.040c | 1.095±0.059c | 2.018±0.057c | 3.156±0.134b |
|  | 513 | 0.425±0.021b | 0.618±0.050b | 1.044±0.078b | 2.099±0.143b | 3.578±0.190b | 4.716±0.523a |
|  | 684 | 0.557±0.015a | 1.146±0.039a | 2.012±0.095a | 4.339±0.523a | 8.030±0.342a |  |
| The ratio of Na^+^/ K^+^ in roots | CK | 0.212±0.002e | 0.208±0.005e | 0.215±0.007e | 0.214±0.003e | 0.214±0.003e | 0.215±0.004d |
|  | 171 | 0.365±0.003d | 0.406±0.020d | 0.516±0.048d | 0.841±0.006d | 1.131±0.008d | 1.389±0.031c |
|  | 342 | 0.648±0.020c | 0.798±0.060c | 1.167±0.054c | 1.614±0.059c | 2.665±0.086c | 3.398±0.150b |
|  | 513 | 0.855±0.058b | 1.088±0.047b | 1.781±0.095b | 2.985±0.170b | 4.390±0.159b | 5.116±0.334a |
|  | 684 | 1.097±0.045a | 1.519±0.113a | 2.634±0.209a | 4.516±0.144a | 8.572±0.306a |  |
| Note: the values displayed are means±SE.; different letters within a variable are significantly different at P < 0.05 | | | | | | | |

**Table S21** Changes of Na^+^/ K^+^ in roots and leaves of *Salix* *gordejevii*.(data in Fig.7C)

| Fig.5c Effects of salt stress on Na+/ K+ in roots and leaves of Salix gordejevii | | | | | | | |
| --- | --- | --- | --- | --- | --- | --- | --- |
| indicators | NaCl Concentration(mM) | NaCl stress treatment time(Days) | | | | | |
|  |  | 1 | 3 | 5 | 8 | 11 | 15 |
| The ratio of Na+/ K+ in leaves | CK | 0.123±0.006e | 0.124±0.006e | 0.125±0.001e | 0.129±0.005e | 0.131±0.003e | 0.130±0.001d |
|  | 171 | 0.196±0.001d | 0.217±0.008d | 0.303±0.016d | 0.432±0.018d | 0.585±0.041d | 0.929±0.081d |
|  | 342 | 0.365±0.004c | 0.435±0.012c | 0.769±0.042c | 1.226±0.060c | 1.788±0.037c | 3.005±0.091c |
|  | 513 | 0.415±0.024b | 0.658±0.007b | 1.236±0.036b | 1.955±0.030b | 3.416±0.171b | 6.264±0.346b |
|  | 684 | 0.486±0.039a | 0.943±0.025a | 1.674±0.063a | 3.202±0.205a | 5.587±0.292a | 8.945±1.108a |
| The ratio of Na+/ K+ in roots | CK | 0.287±0.005e | 0.287±0.012e | 0.285±0.006e | 0.284±0.009e | 0.298±0.006e | 0.291±0.002e |
|  | 171 | 0.510±0.025d | 0.574±0.012d | 0.715±0.015d | 0.930±0.043d | 1.203±0.078d | 1.396±0.012d |
|  | 342 | 0.868±0.022c | 1.024±0.061c | 1.402±0.017c | 1.891±0.105c | 2.421±0.077c | 3.404±0.109c |
|  | 513 | 1.048±0.073b | 1.334±0.040b | 2.081±0.070b | 2.994±0.057b | 4.242±0.132b | 5.589±0.338b |
|  | 684 | 1.311±0.023a | 1.772±0.942a | 2.839±0.158a | 4.472±0.288a | 5.930±0.275a | 7.644±0.217a |
| Note: the values displayed are means±SE.; different letters within a variable are significantly different at P < 0.05 | | | | | | | |

**Table S22** Changes of Na^+^/ Ca^2+^ in roots and leaves of *Salix* *linearistipularis*.(data in Fig.8A)

| indicators | NaCl Concentration(mM) | NaCl stress treatment time(Days) | | | | | |
| --- | --- | --- | --- | --- | --- | --- | --- |
|  |  | 1 | 3 | 5 | 8 | 11 | 15 |
| The ratio of Na^+^/ Ca^2+^ in leaves | CK | 0.452±0.005e | 0.457±0.002e | 0.469±0.014e | 0.485±0.006e | 0.469±0.022e | 0.458±0.015c |
|  | 171 | 0.770±0.084d | 0.800±0.022d | 1.474±0.027d | 2.134±0.087d | 3.820±0.231d | 6.008±0.374b |
|  | 342 | 1.327±0.061c | 1.561±0.088c | 2.883±0.131c | 4.313±0.265c | 9.274±0.233c | 16.320±0.894a |
|  | 513 | 2.199±0.145b | 3.226±0.108b | 5.318±0.237b | 8.280±0.129b | 16.171±0.955b |  |
|  | 684 | 2.973±0.081a | 4.461±0.233a | 8.254±0.093a | 11.216±0.212a | 27.784±0.796a |  |
| The ratio of Na^+^/ Ca^2+^ in roots | CK | 1.857±0.082e | 1.975±0.112e | 1.989±0.139e | 2.057±0.149e | 2.012±0.118e | 1.984±0.019c |
|  | 171 | 3.312±0.332d | 4.492±0.165d | 5.625±0.054d | 7.305±0.066d | 12.637±0.815d | 19.048±1.395b |
|  | 342 | 5.321±0.094c | 6.948±0.274c | 11.133±0.162c | 14.968±0.415c | 28.767±0.892c | 48.996±2.308a |
|  | 513 | 6.640±0.128b | 9.710±0.354b | 17.258±0.795b | 24.284±2.451b | 45.501±4.028b |  |
|  | 684 | 9.183±0.362a | 13.432±0.707a | 25.566±2.290a | 31.465±0.426a | 73.971±6.128a |  |
| Note: the values displayed are means±SE.; different letters within a variable are significantly different at P < 0.05 | | | | | | | |

**Table S23** Changes of Na^+^/ Ca^2+^ in roots and leaves of *Salix* *matsudana*.(data in Fig.8B)

| indicators | NaCl Concentration(mM) | NaCl stress treatment time(Days) | | | | | |
| --- | --- | --- | --- | --- | --- | --- | --- |
|  |  | 1 | 3 | 5 | 8 | 11 | 15 |
| The ratio of Na^+^/ Ca^2+^ in leaves | CK | 0.730±0.015e | 0.734±0.024e | 0.736±0.016e | 0.746±0.031e | 0.754±0.041e | 0.773±0.058d |
|  | 171 | 1.127±0.033d | 1.449±0.110d | 1.961±0.039d | 3.057±0.249d | 4.168±0.071d | 6.237±0.114c |
|  | 342 | 1.694±0.158c | 2.743±0.081c | 3.993±0.241c | 6.505±0.318c | 9.147±0.326c | 14.354±0.571b |
|  | 513 | 2.171±0.085b | 4.557±0.323b | 6.663±0.251b | 10.380±0.490b | 13.860±0.817b | 25.855±2.602a |
|  | 684 | 3.040±0.067a | 8.060±0.315a | 10.243±0.465a | 16.708±0.892a | 31.349±0.731a |  |
| The ratio of Na^+^/ Ca^2+^ in roots | CK | 2.814±0.086e | 2.952±0.093e | 2.844±0.100e | 2.931±0.093e | 2.932±0.083e | 3.240±0.132d |
|  | 171 | 4.994±0.185d | 5.716±0.226d | 7.515±0.760d | 11.807±0.389d | 15.278±0.119d | 22.594±1.479c |
|  | 342 | 7.716±0.197c | 10.798±0.346c | 15.147±0.043c | 24.835±0.821c | 33.351±1.334c | 51.678±1.086b |
|  | 513 | 11.693±0.947b | 18.706±0.764b | 24.542±0.764b | 38.915±1.624b | 50.720±3.853b | 80.740±4.239a |
|  | 684 | 16.339±0.332a | 27.969±1.170a | 34.441±0.803a | 54.089±2.078a | 101.710±9.624a |  |
| Note: the values displayed are means±SE.; different letters within a variable are significantly different at P < 0.05 | | | | | | | |

**Table S24** Changes of Na^+^/ Ca^2+^ in roots and leaves of *Salix* *gordejevii*.(data in Fig.8C)

| indicators | NaCl Concentration(mM) | NaCl stress treatment time(Days) | | | | | |
| --- | --- | --- | --- | --- | --- | --- | --- |
|  |  | 1 | 3 | 5 | 8 | 11 | 15 |
| The ratio of Na+/ Ca2+ in leaves | CK | 1.475±0.085e | 1.533±0.143e | 1.516±0.035e | 1.535±0.014e | 1.602±0.005e | 1.611±0.017e |
|  | 171 | 2.277±0.110d | 2.326±0.121d | 3.545±0.163d | 5.782±0.425d | 8.357±0.386d | 12.642±0.973d |
|  | 342 | 3.068±0.059c | 4.413±0.427c | 8.311±0.524c | 13.344±1.098c | 19.520±0.666c | 26.442±0.782c |
|  | 513 | 3.926±0.164b | 6.352±0.453b | 12.052±0.459b | 18.206±1.415b | 35.255±3.283b | 51.185±3.827b |
|  | 684 | 4.673±0.246a | 8.833±0.548a | 16.626±0.660a | 25.379±0.899a | 50.790±1.015a | 87.776±5.935a |
| The ratio of Na+/ Ca2+ in roots | CK | 4.239±0.231e | 4.570±0.115e | 4.413±0.216e | 4.546±0.170e | 4.631±0.044e | 4.540±0.293e |
|  | 171 | 7.490±0.302d | 9.400±0.328d | 11.269±0.611d | 13.824±0.255d | 18.642±0.955d | 24.574±0.986d |
|  | 342 | 12.260±1.088c | 14.783±0.883c | 21.391±1.339c | 27.147±0.523c | 41.491±5.325c | 52.711±2.972c |
|  | 513 | 13.922±0.612b | 17.903±0.961b | 29.774±0.769b | 38.538±0.729b | 64.222±2.113b | 86.796±4.063b |
|  | 684 | 17.586±0.741a | 24.588±2.378a | 41.516±3.246a | 53.566±2.815a | 89.693±6.301a | 129.767±7.986a |
| Note: the values displayed are means±SE.; different letters within a variable are significantly different at P < 0.05 | | | | | | | |

**Table S25** Correlation of Na^+^in roots and leaves of three willows under salt stress.(data in Fig.9A)

| indicators | NaCl Concentration(mM) | NaCl stress treatment time(Days) | | | | | |
| --- | --- | --- | --- | --- | --- | --- | --- |
|  |  | 1 | 3 | 5 | 8 | 11 | 15 |
| Na^+^ content in *Salix linearistipularis* leaves(mg/g) | CK | 1.436±0.027e | 1.448±0.069e | 1.454±0.081e | 1.481±0.060e | 1.453±0.044e | 1.442±0.034c |
|  | 171 | 2.304±0.258d | 2.438±0.194d | 4.077±0.176d | 5.374±0.231d | 6.647±0.297d | 8.555±0.378b |
|  | 342 | 3.920±0.116c | 4.621±0.170c | 7.143±0.202c | 8.347±0.303c | 11.268±0.539c | 13.593±0.322a |
|  | 513 | 6.486±0.300b | 8.300±0.228b | 10.169±0.747b | 12.477±0.551b | 15.799±0.538b |  |
|  | 684 | 8.489±0.165a | 10.877±0.608a | 13.905±1.177a | 14.227±1.307a | 18.750±0.890a |  |
| Na^+^ content in *Salix linearistipularis* roots(mg/g) | CK | 3.289±0.146e | 3.444±0.165e | 3.441±0.126e | 3.475±0.239d | 3.496±0.194e | 3.443±0.045c |
|  | 171 | 5.533±0.345d | 7.271±0.330d | 8.375±0.329d | 9.838±0.578c | 11.953±0.623d | 11.959±0.235b |
|  | 342 | 8.789±0.537c | 10.308±0.521c | 12.518±0.735c | 14.695±0.648b | 15.716±0.407c | 16.367±0.926a |
|  | 513 | 10.227±0.468b | 11.836±0.747b | 14.882±0.459b | 15.835±0.916ab | 17.972±0.636b |  |
|  | 684 | 12.328±0.518a | 14.487±0.709a | 16.093±0.611a | 17.491±0.775a | 19.443±0.850a |  |
| Na^+^ content in *Salix matsudana* leaves(mg/g) | CK | 4.052±0.159e | 4.038±0.118e | 4.045±0.020e | 4.076±0.209e | 4.161±0.147e | 4.246±0.118d |
|  | 171 | 6.206±0.132d | 7.634±0.459d | 8.586±0.184d | 12.052±0.780d | 14.885±0.918d | 18.795±0.906c |
|  | 342 | 8.920±0.499c | 9.807±0.630c | 13.942±0.684c | 21.049±0.833c | 26.066±0.689c | 33.184±2.790b |
|  | 513 | 11.229±0.449b | 15.028±1.293b | 21.411±0.881b | 28.842±0.931b | 34.227±1.420b | 39.972±3.709a |
|  | 684 | 14.500±1.450a | 24.655±0.973a | 31.249±0.778a | 43.365±2.276a | 54.004±2.312a |  |
| Na^+^ content in *Salix matsudana* roots(mg/g) | CK | 7.541±0.045e | 7.615±0.009e | 7.629±0.032e | 7.750±0.057e | 7.839±0.062e | 7.989±0.067d |
|  | 171 | 13.013±0.760d | 14.841±0.580d | 18.654±1.675d | 23.084±2.851d | 23.716±0.437d | 25.766±0.880c |
|  | 342 | 20.312±0.868c | 22.096±2.061c | 26.367±1.512c | 30.073±1.690c | 31.080±0.935c | 33.248±1.120b |
|  | 513 | 24.502±1.204b | 26.759±1.292b | 30.011±1.354b | 33.713±0.859b | 34.491±1.423b | 37.588±2.227a |
|  | 684 | 28.690±0.434a | 34.796±0.851a | 36.985±2.364a | 42.423±1.015a | 56.461±2.671a |  |
| Na^+^ content in *Salix gordejevii* leaves(mg/g) | CK | 3.431±0.244e | 3.541±0.278e | 3.486±0.124e | 3.525±0.167e | 3.625±0.051e | 3.618±0.016e |
|  | 171 | 5.406±0.240d | 5.458±0.252d | 7.433±0.341d | 9.726±0.350d | 11.986±0.376d | 15.800±0.846d |
|  | 342 | 7.929±0.250c | 9.188±0.625c | 15.861±0.522c | 19.301±0.716c | 22.058±0.745c | 28.675±0.989c |
|  | 513 | 9.206±0.684b | 12.530±0.535b | 21.687±0.286b | 23.485±0.412b | 33.904±1.767b | 38.348±2.425b |
|  | 684 | 11.015±0.622a | 15.698±0.495a | 26.334±0.855a | 29.369±1.262a | 37.206±1.556a | 41.202±1.996a |
| Na^+^ content in *Salix gordejevii* roots(mg/g) | CK | 6.676±0.026e | 6.703±0.406e | 6.658±0.130e | 6.589±0.323e | 6.896±0.232e | 6.765±0.404e |
|  | 171 | 11.525±0.465d | 13.675±0.499d | 16.015±0.711d | 18.560±0.867d | 21.006±1.408d | 23.589±1.544d |
|  | 342 | 17.792±0.870c | 20.257±0.757c | 24.499±1.375c | 27.982±0.743c | 29.055±2.182c | 32.238±0.878c |
|  | 513 | 20.080±1.218b | 23.006±1.313b | 28.255±0.920b | 33.683±1.465b | 35.939±1.633b | 38.638±1.417b |
|  | 684 | 23.701±1.167a | 28.004±0.818a | 31.911±1.589a | 35.399±1.587a | 41.748±1.680a | 44.506±2.544a |
| Note: the values displayed are means±SE.; different letters within a variable are significantly different at P < 0.05 | | | | | | | |

**Table S26** Correlation of Na^+^/K^+^ in roots and leaves of three willows under salt stress.(data in Fig.9B)

| indicators | NaCl Concentration(mM) | NaCl stress treatment time(Days) | | | | | |
| --- | --- | --- | --- | --- | --- | --- | --- |
|  |  | 1 | 3 | 5 | 8 | 11 | 15 |
| Na^+^/K^+^ content in *Salix linearistipularis* leaves(mg/g) | CK | 0.059±0.002e | 0.062±0.003e | 0.060±0.001e | 0.061±0.004e | 0.060±0.004e | 0.059±0.002c |
|  | 171 | 0.095±0.011d | 0.103±0.007d | 0.180±0.004d | 0.258±0.016d | 0.414±0.023d | 0.784±0.039b |
|  | 342 | 0.158±0.002c | 0.242±0.008c | 0.411±0.009c | 0.596±0.038c | 1.110±0.031c | 2.094±0.070a |
|  | 513 | 0.280±0.016b | 0.464±0.016b | 0.683±0.022b | 1.089±0.062b | 2.396±0.053b |  |
|  | 684 | 0.509±0.012a | 0.696±0.021a | 1.059±0.076a | 1.924±0.113a | 3.672±0.182a |  |
| Na^+^/K^+^content in *Salix linearistipularis* roots(mg/g) | CK | 0.226±0.004e | 0.236±0.015e | 0.236±0.004e | 0.244±0.018e | 0.242±0.002e | 0.239±0.003c |
|  | 171 | 0.449±0.055d | 0.573±0.004d | 0.741±0.053d | 0.935±0.050d | 1.393±0.114d | 2.606±0.098b |
|  | 342 | 0.820±0.048c | 1.068±0.055c | 1.690±0.029c | 2.144±0.079c | 3.033±0.131c | 5.609±0.488a |
|  | 513 | 1.086±0.012b | 1.784±0.125b | 2.603±0.122b | 3.554±0.133b | 6.366±0.090b |  |
|  | 684 | 1.957±0.081a | 2.714±0.132a | 3.779±0.348a | 6.262±0.332a | 9.815±0.109a |  |
| Na^+^/K^+^ content in *Salix matsudana* leaves(mg/g) | CK | 0.103±0.008e | 0.107±0.002e | 0.105±0.002e | 0.108±0.002e | 0.110±0.006e | 0.111±0.005d |
|  | 171 | 0.154±0.007d | 0.204±0.009d | 0.232±0.017d | 0.437±0.028d | 0.666±0.054d | 0.915±0.062c |
|  | 342 | 0.315±0.019c | 0.375±0.006c | 0.585±0.040c | 1.095±0.059c | 2.018±0.057c | 3.156±0.134b |
|  | 513 | 0.425±0.021b | 0.618±0.050b | 1.044±0.078b | 2.099±0.143b | 3.578±0.190b | 4.716±0.523a |
|  | 684 | 0.557±0.015a | 1.146±0.039a | 2.012±0.095a | 4.339±0.523a | 8.030±0.342a |  |
| Na^+^/K^+^ content in *Salix matsudana* roots(mg/g) | CK | 0.212±0.002e | 0.208±0.005e | 0.215±0.007e | 0.214±0.003e | 0.214±0.003e | 0.215±0.004d |
|  | 171 | 0.365±0.003d | 0.406±0.020d | 0.516±0.048d | 0.841±0.006d | 1.131±0.008d | 1.389±0.031c |
|  | 342 | 0.648±0.020c | 0.798±0.060c | 1.167±0.054c | 1.614±0.059c | 2.665±0.086c | 3.398±0.150b |
|  | 513 | 0.855±0.058b | 1.088±0.047b | 1.781±0.095b | 2.985±0.170b | 4.390±0.159b | 5.116±0.334a |
|  | 684 | 1.097±0.045a | 1.519±0.113a | 2.634±0.209a | 4.516±0.144a | 8.572±0.306a |  |
| Na^+^/K^+^ content in *Salix gordejevii* leaves(mg/g) | CK | 0.123±0.006e | 0.124±0.006e | 0.125±0.001e | 0.129±0.005e | 0.131±0.003e | 0.130±0.001d |
|  | 171 | 0.196±0.001d | 0.217±0.008d | 0.303±0.016d | 0.432±0.018d | 0.585±0.041d | 0.929±0.081d |
|  | 342 | 0.365±0.004c | 0.435±0.012c | 0.769±0.042c | 1.226±0.060c | 1.788±0.037c | 3.005±0.091c |
|  | 513 | 0.415±0.024b | 0.658±0.007b | 1.236±0.036b | 1.955±0.030b | 3.416±0.171b | 6.264±0.346b |
|  | 684 | 0.486±0.039a | 0.943±0.025a | 1.674±0.063a | 3.202±0.205a | 5.587±0.292a | 8.945±1.108a |
| Na^+^/K^+^ content in *Salix gordejevii* roots(mg/g) | CK | 0.287±0.005e | 0.287±0.012e | 0.285±0.006e | 0.284±0.009e | 0.298±0.006e | 0.291±0.002e |
|  | 171 | 0.510±0.025d | 0.574±0.012d | 0.715±0.015d | 0.930±0.043d | 1.203±0.078d | 1.396±0.012d |
|  | 342 | 0.868±0.022c | 1.024±0.061c | 1.402±0.017c | 1.891±0.105c | 2.421±0.077c | 3.404±0.109c |
|  | 513 | 1.048±0.073b | 1.334±0.040b | 2.081±0.070b | 2.994±0.057b | 4.242±0.132b | 5.589±0.338b |
|  | 684 | 1.311±0.023a | 1.772±0.942a | 2.839±0.158a | 4.472±0.288a | 5.930±0.275a | 7.644±0.217a |
| Note: the values displayed are means±SE.; different letters within a variable are significantly different at P < 0.05 | | | | | | | |

**Table S27** Correlation of Na^+^/Ca^2+^ in roots and leaves of three willows under salt stress.(data in Fig.9C)

| indicators | NaCl Concentration(mM) | NaCl stress treatment time(Days) | | | | | |
| --- | --- | --- | --- | --- | --- | --- | --- |
|  |  | 1 | 3 | 5 | 8 | 11 | 15 |
| Na^+^/Ca^2+^ content in *Salix linearistipularis* leaves(mg/g) | CK | 0.452±0.005e | 0.457±0.002e | 0.469±0.014e | 0.485±0.006e | 0.469±0.022e | 0.458±0.015c |
|  | 171 | 0.770±0.084d | 0.800±0.022d | 1.474±0.027d | 2.134±0.087d | 3.820±0.231d | 6.008±0.374b |
|  | 342 | 1.327±0.061c | 1.561±0.088c | 2.883±0.131c | 4.313±0.265c | 9.274±0.233c | 16.320±0.894a |
|  | 513 | 2.199±0.145b | 3.226±0.108b | 5.318±0.237b | 8.280±0.129b | 16.171±0.955b |  |
|  | 684 | 2.973±0.081a | 4.461±0.233a | 8.254±0.093a | 11.216±0.212a | 27.784±0.796a |  |
| Na^+^/Ca^2+^content in *Salix linearistipularis* roots(mg/g) | CK | 1.857±0.082e | 1.975±0.112e | 1.989±0.139e | 2.057±0.149e | 2.012±0.118e | 1.984±0.019c |
|  | 171 | 3.312±0.332d | 4.492±0.165d | 5.625±0.054d | 7.305±0.066d | 12.637±0.815d | 19.048±1.395b |
|  | 342 | 5.321±0.094c | 6.948±0.274c | 11.133±0.162c | 14.968±0.415c | 28.767±0.892c | 48.996±2.308a |
|  | 513 | 6.640±0.128b | 9.710±0.354b | 17.258±0.795b | 24.284±2.451b | 45.501±4.028b |  |
|  | 684 | 9.183±0.362a | 13.432±0.707a | 25.566±2.290a | 31.465±0.426a | 73.971±6.128a |  |
| Na^+^/Ca^2+^ content in *Salix matsudana* leaves(mg/g) | CK | 0.730±0.015e | 0.734±0.024e | 0.736±0.016e | 0.746±0.031e | 0.754±0.041e | 0.773±0.058d |
|  | 171 | 1.127±0.033d | 1.449±0.110d | 1.961±0.039d | 3.057±0.249d | 4.168±0.071d | 6.237±0.114c |
|  | 342 | 1.694±0.158c | 2.743±0.081c | 3.993±0.241c | 6.505±0.318c | 9.147±0.326c | 14.354±0.571b |
|  | 513 | 2.171±0.085b | 4.557±0.323b | 6.663±0.251b | 10.380±0.490b | 13.860±0.817b | 25.855±2.602a |
|  | 684 | 3.040±0.067a | 8.060±0.315a | 10.243±0.465a | 16.708±0.892a | 31.349±0.731a |  |
| Na^+^/Ca^2+^ content in *Salix matsudana* roots(mg/g) | CK | 2.814±0.086e | 2.952±0.093e | 2.844±0.100e | 2.931±0.093e | 2.932±0.083e | 3.240±0.132d |
|  | 171 | 4.994±0.185d | 5.716±0.226d | 7.515±0.760d | 11.807±0.389d | 15.278±0.119d | 22.594±1.479c |
|  | 342 | 7.716±0.197c | 10.798±0.346c | 15.147±0.043c | 24.835±0.821c | 33.351±1.334c | 51.678±1.086b |
|  | 513 | 11.693±0.947b | 18.706±0.764b | 24.542±0.764b | 38.915±1.624b | 50.720±3.853b | 80.740±4.239a |
|  | 684 | 16.339±0.332a | 27.969±1.170a | 34.441±0.803a | 54.089±2.078a | 101.710±9.624a |  |
| Na^+^/Ca^2+^ content in *Salix gordejevii* leaves(mg/g) | CK | 1.475±0.085e | 1.533±0.143e | 1.516±0.035e | 1.535±0.014e | 1.602±0.005e | 1.611±0.017e |
|  | 171 | 2.277±0.110d | 2.326±0.121d | 3.545±0.163d | 5.782±0.425d | 8.357±0.386d | 12.642±0.973d |
|  | 342 | 3.068±0.059c | 4.413±0.427c | 8.311±0.524c | 13.344±1.098c | 19.520±0.666c | 26.442±0.782c |
|  | 513 | 3.926±0.164b | 6.352±0.453b | 12.052±0.459b | 18.206±1.415b | 35.255±3.283b | 51.185±3.827b |
|  | 684 | 4.673±0.246a | 8.833±0.548a | 16.626±0.660a | 25.379±0.899a | 50.790±1.015a | 87.776±5.935a |
| Na^+^/Ca^2+^ content in *Salix gordejevii* roots(mg/g) | CK | 4.239±0.231e | 4.570±0.115e | 4.413±0.216e | 4.546±0.170e | 4.631±0.044e | 4.540±0.293e |
|  | 171 | 7.490±0.302d | 9.400±0.328d | 11.269±0.611d | 13.824±0.255d | 18.642±0.955d | 24.574±0.986d |
|  | 342 | 12.260±1.088c | 14.783±0.883c | 21.391±1.339c | 27.147±0.523c | 41.491±5.325c | 52.711±2.972c |
|  | 513 | 13.922±0.612b | 17.903±0.961b | 29.774±0.769b | 38.538±0.729b | 64.222±2.113b | 86.796±4.063b |
|  | 684 | 17.586±0.741a | 24.588±2.378a | 41.516±3.246a | 53.566±2.815a | 89.693±6.301a | 129.767±7.986a |
| Note: the values displayed are means±SE.; different letters within a variable are significantly different at P < 0.05 | | | | | | | |

**Figure S1** Design of three willows experimental treatment.(data in Fig.1)


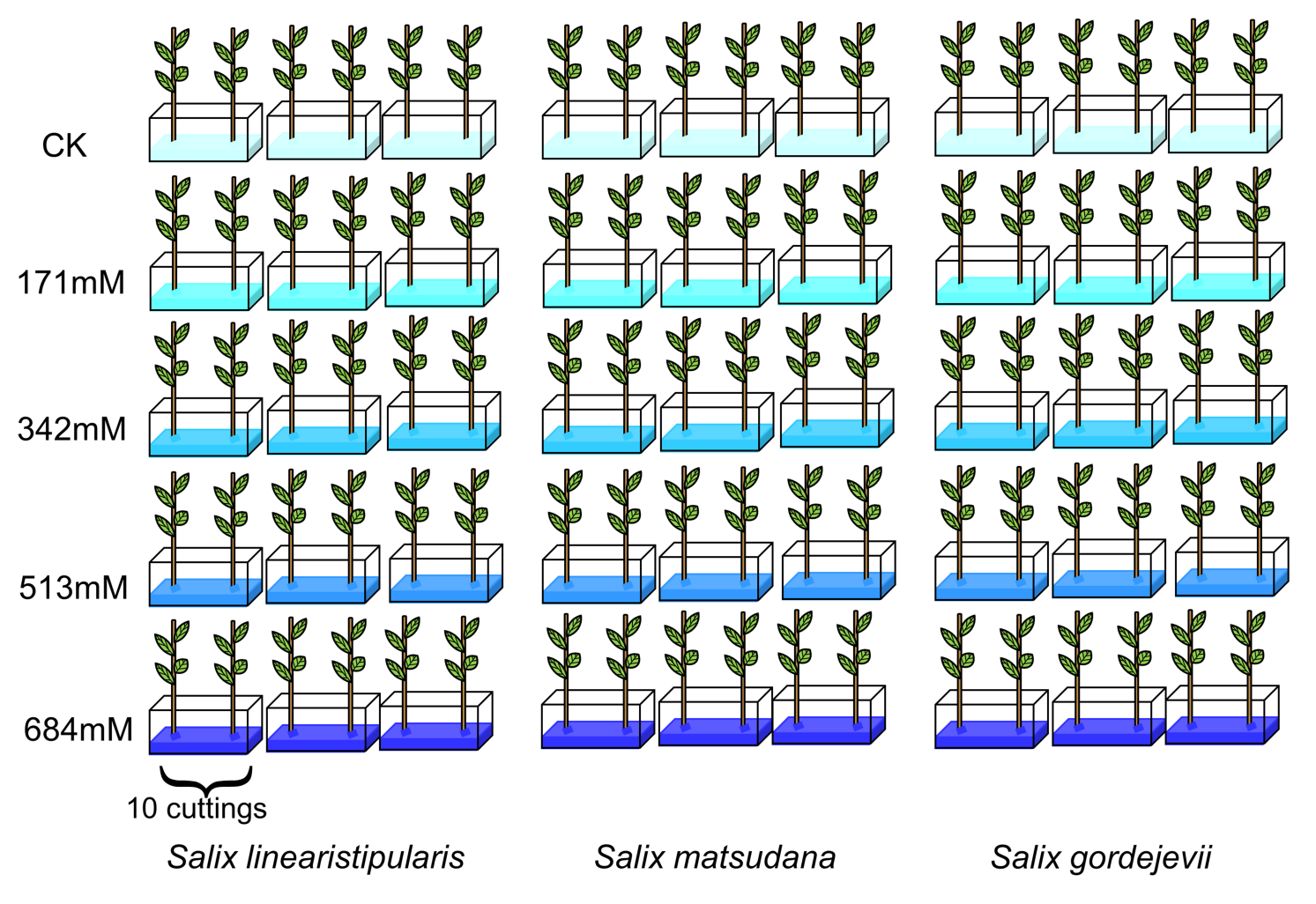


**Figure S2** Phenotypic response of *Salix linearistipularis* to salt stress.(data in Fig.2A)


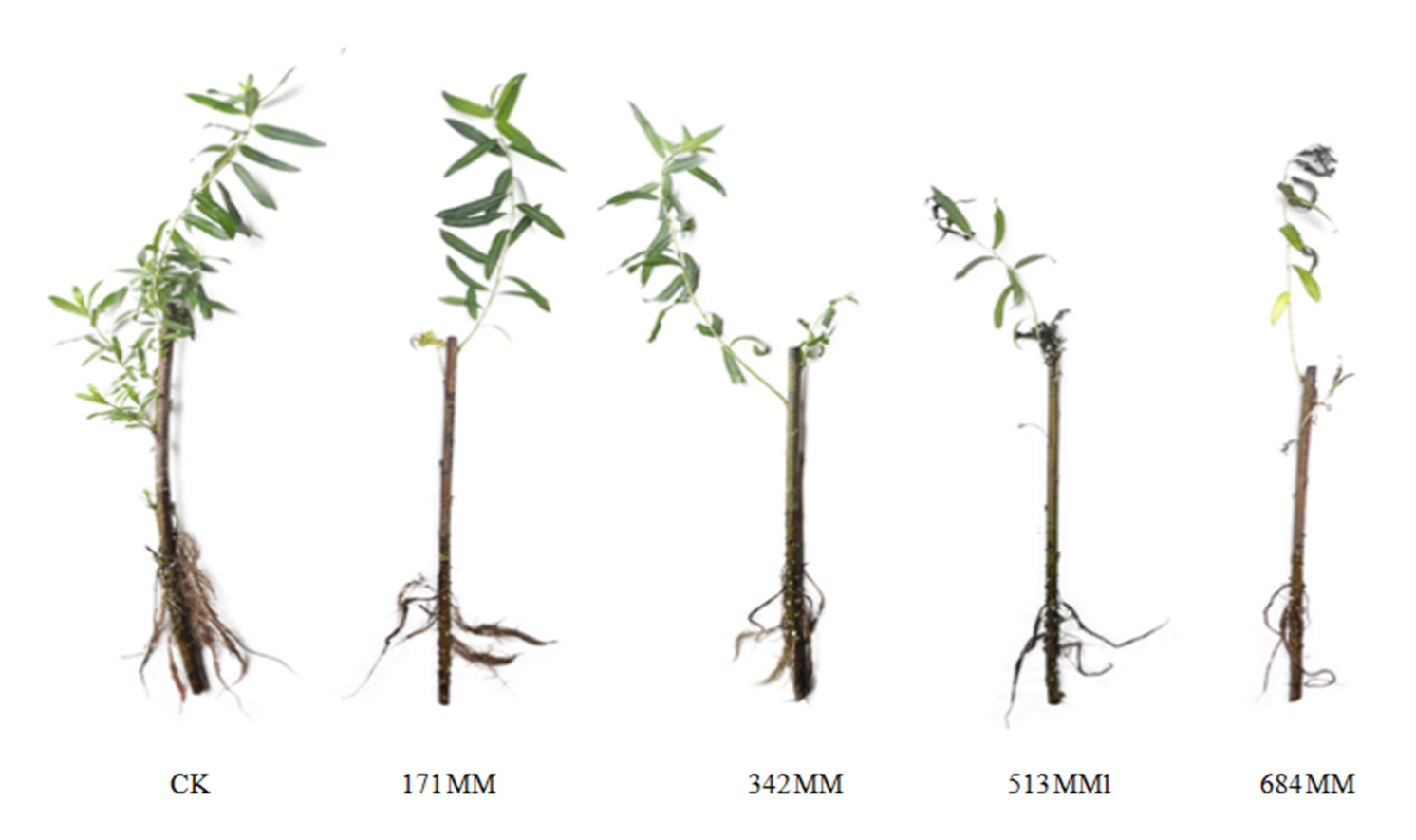


**Figure S3** Phenotypic response of *Salix matsudana* to salt stress.(data in Fig.2B)


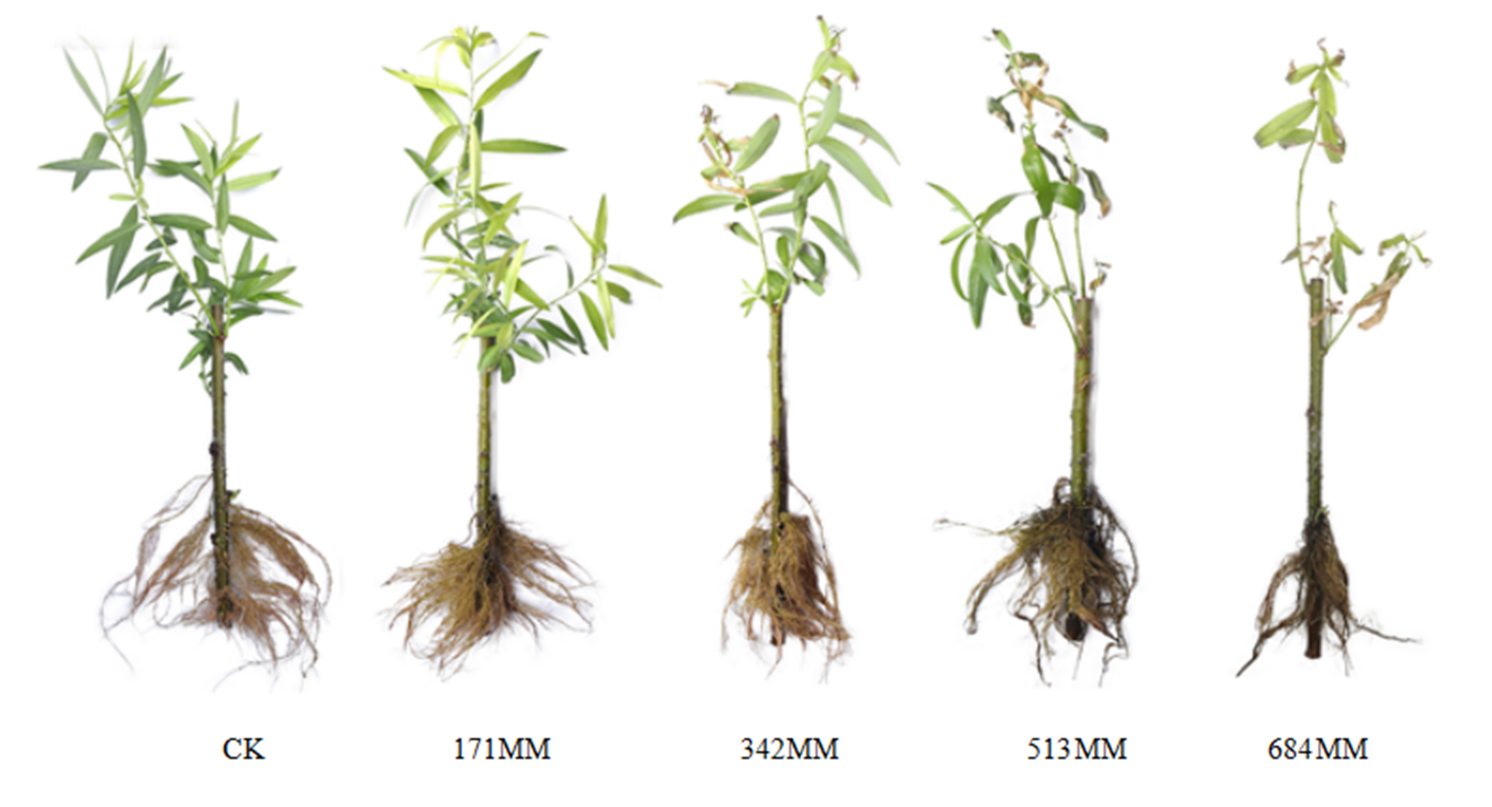


**Figure S4** Phenotypic response of *Salix gordejevii* to salt stress.(data in Fig.2C)


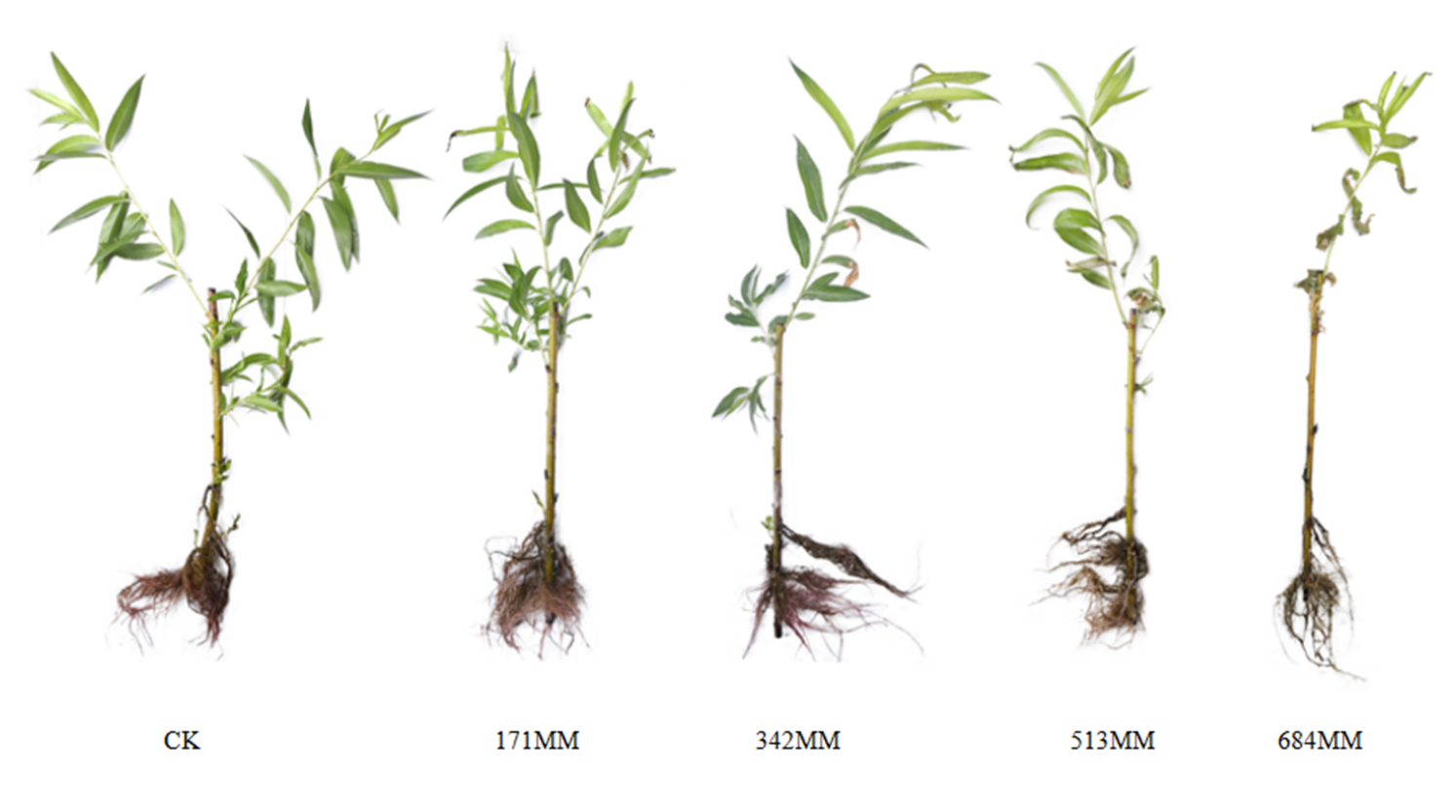


**Figure S5** Effects of salt stress on Na^+^, Ca^2+^ and K^+^ contents in roots and leaves of *Salix* *linearistipularis*.(data in Fig.3A)


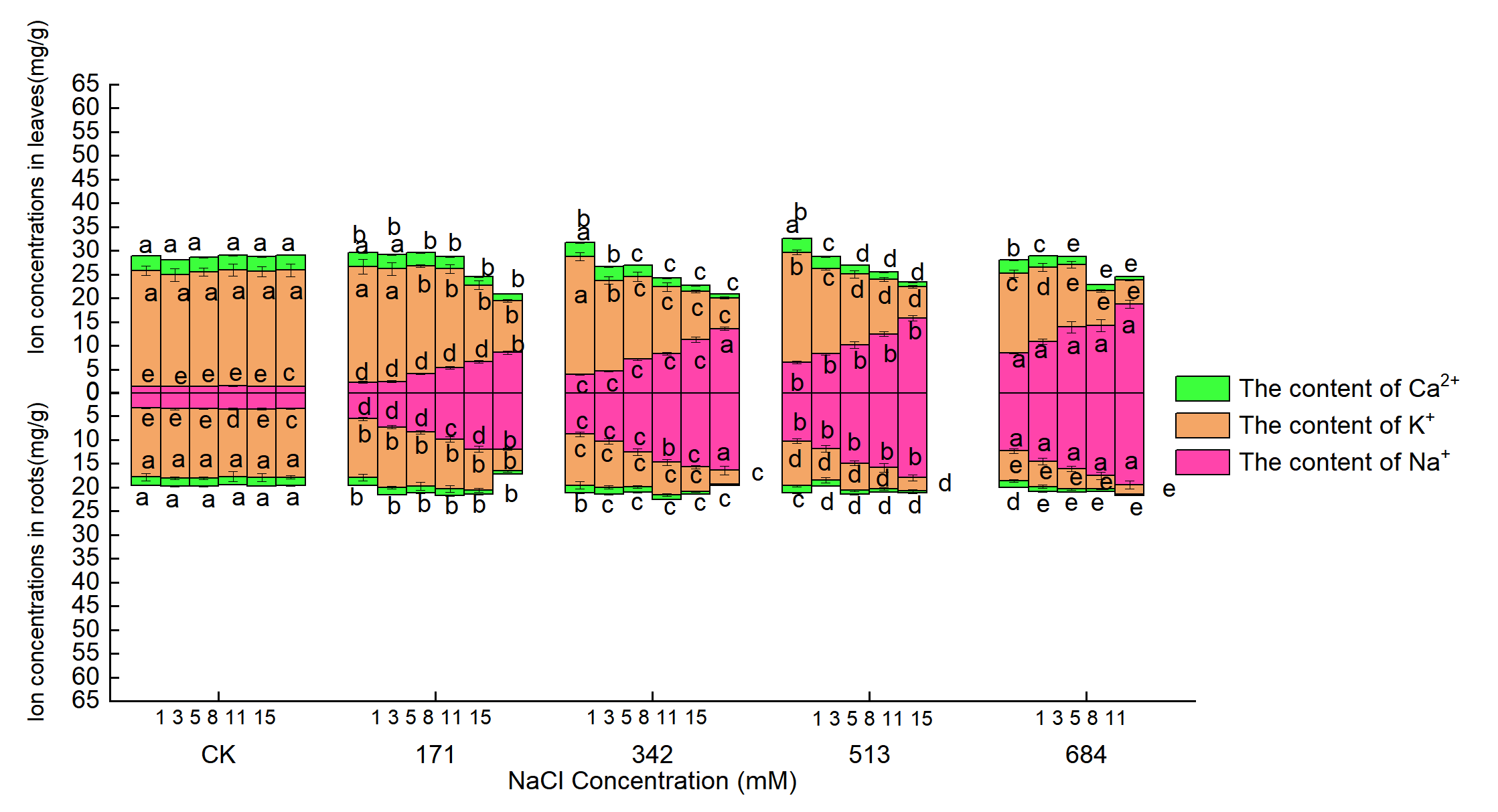


**Figure S6** Effects of salt stress on Na^+^, Ca^2+^ and K^+^ contents in roots and leaves of *Salix* *matsudana*.(data in Fig.3B)


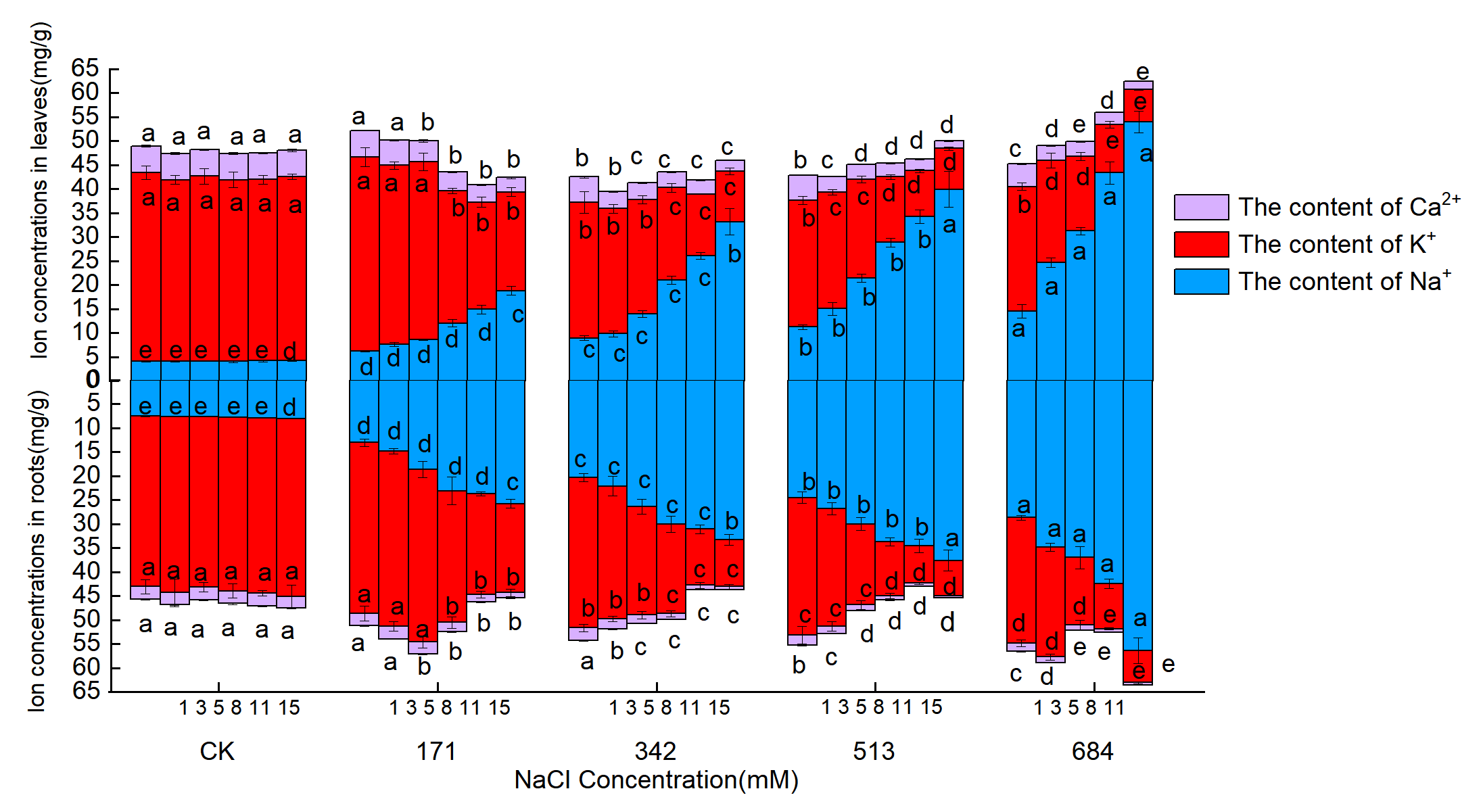
**Figure S7** Effects of salt stress on Na^+^, Ca^2+^ and K^+^ contents in roots and leaves of *Salix* *gordejevii*.(data in Fig.3C)


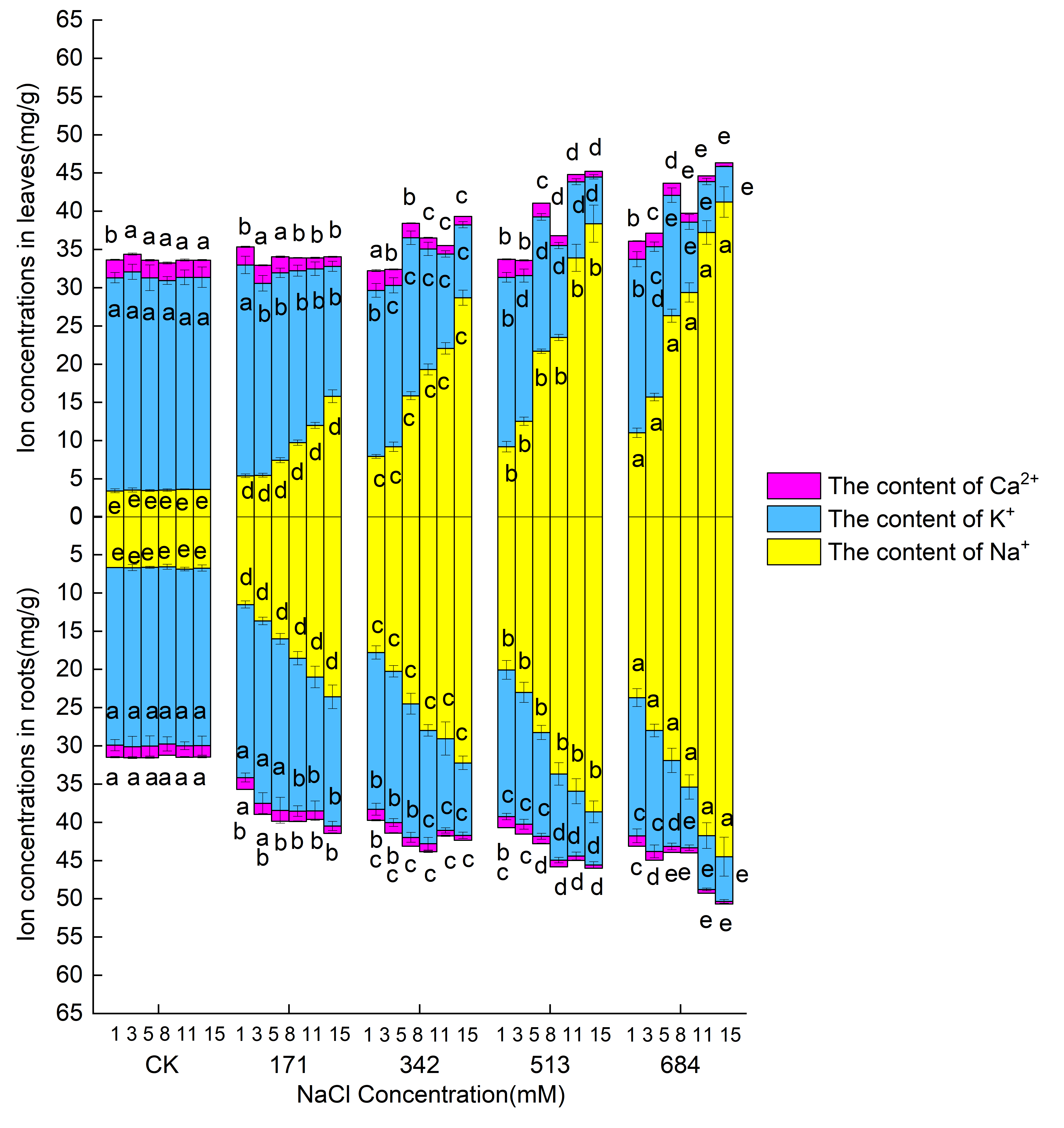


**Figure S8** Ratio of Na^+^ to CK in roots/leaves of *Salix* *linearistipularis*.(data in Fig.4A)


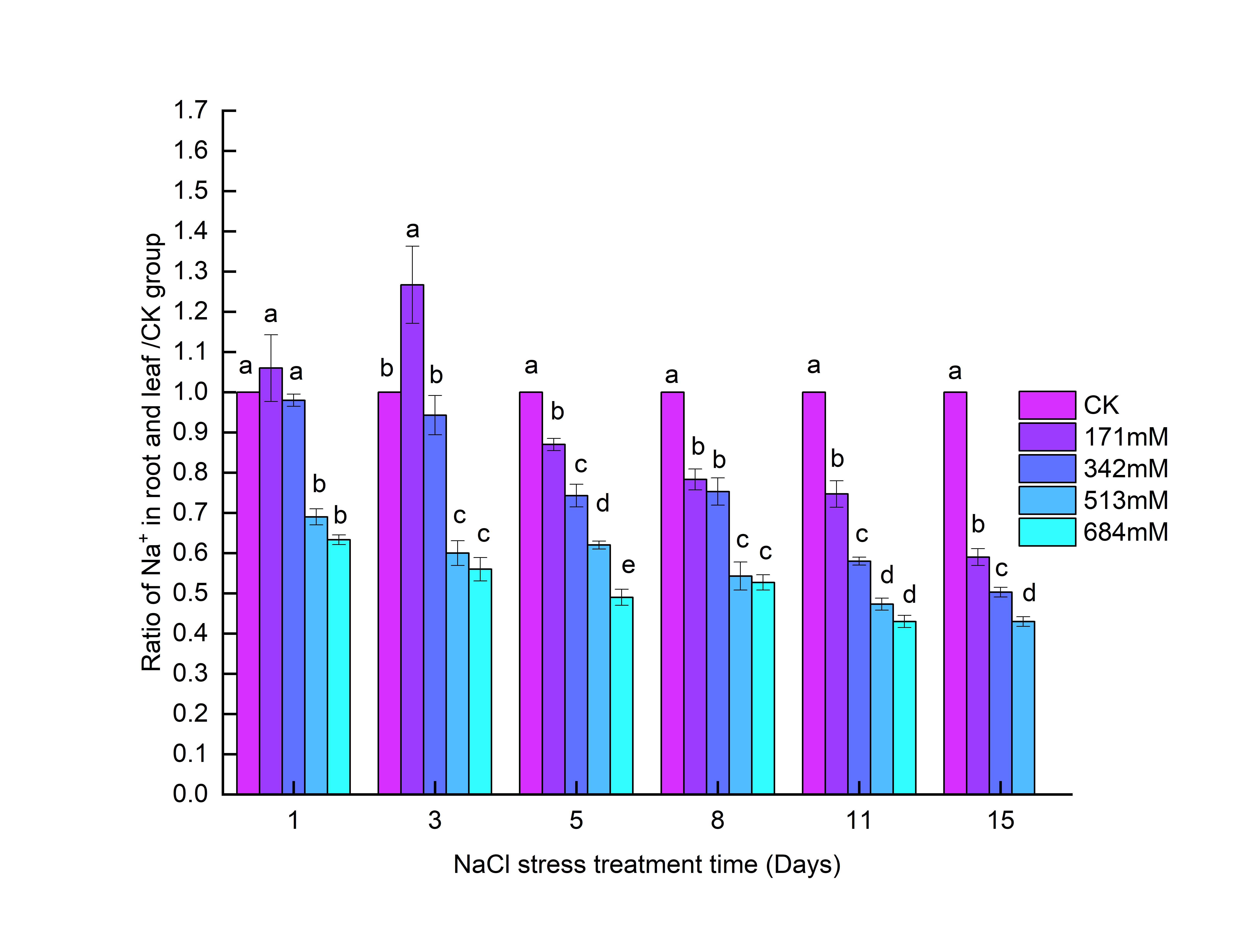


**Figure S9** Ratio of Na^+^ to CK in roots/leaves of *Salix* *matsudana*.(data in Fig.4B)


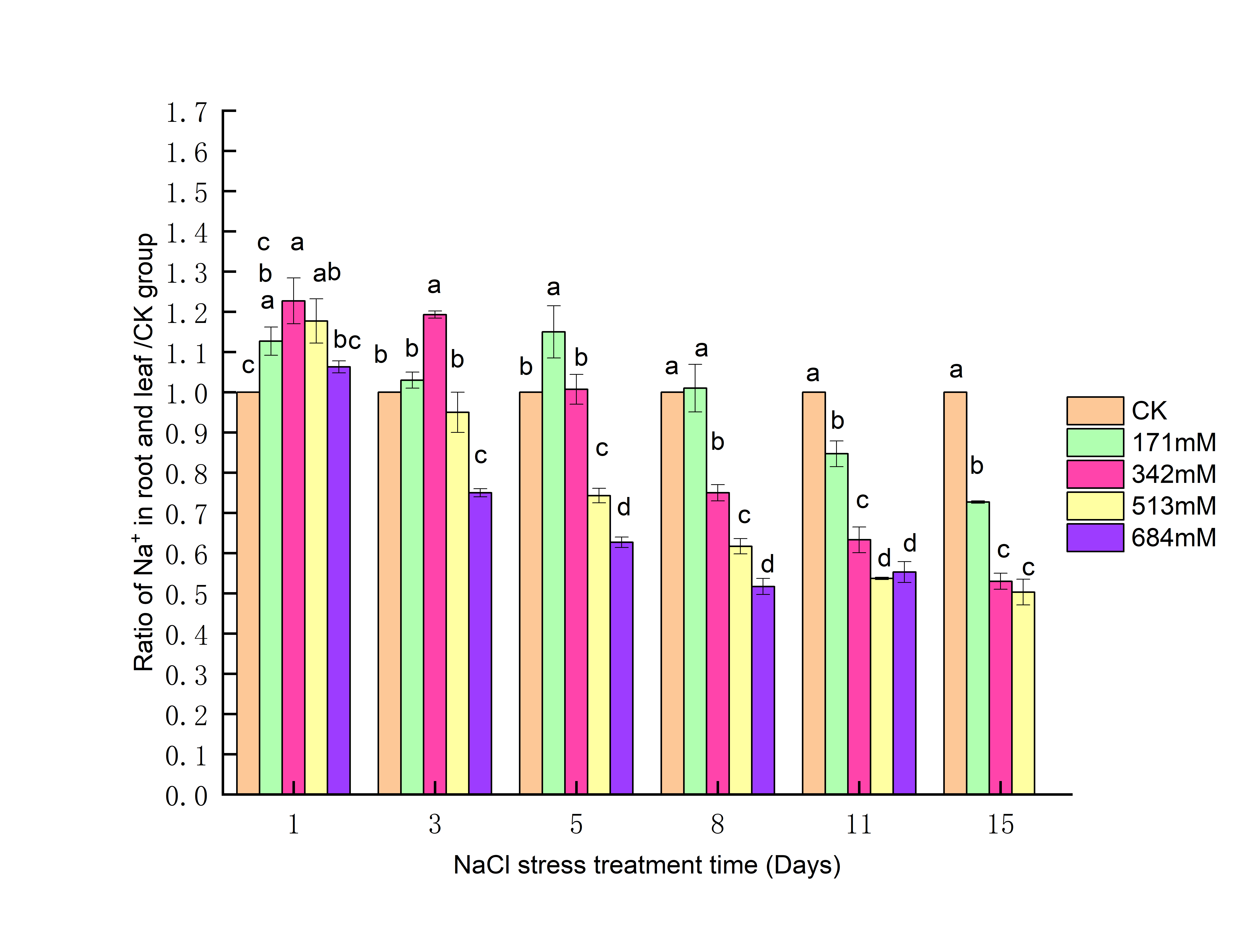


**Figure S10** Ratio of Na^+^ to CK in roots/leaves of *Salix* *gordejevii*.(data in Fig.4C)


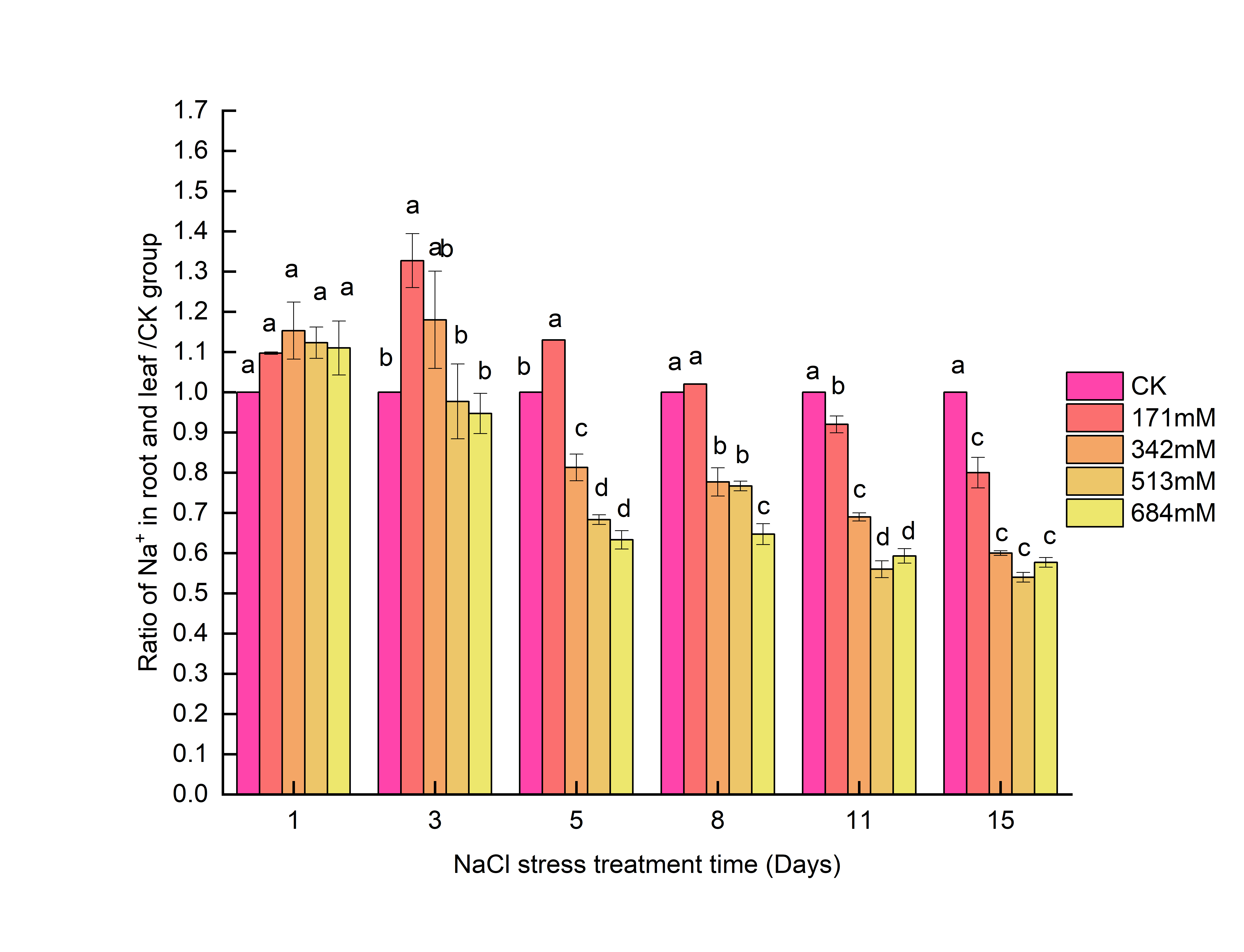


**Figure S11** Ratio of K^+^ to CK in roots/leaves of *Salix* *linearistipularis*.(data in Fig.4D)


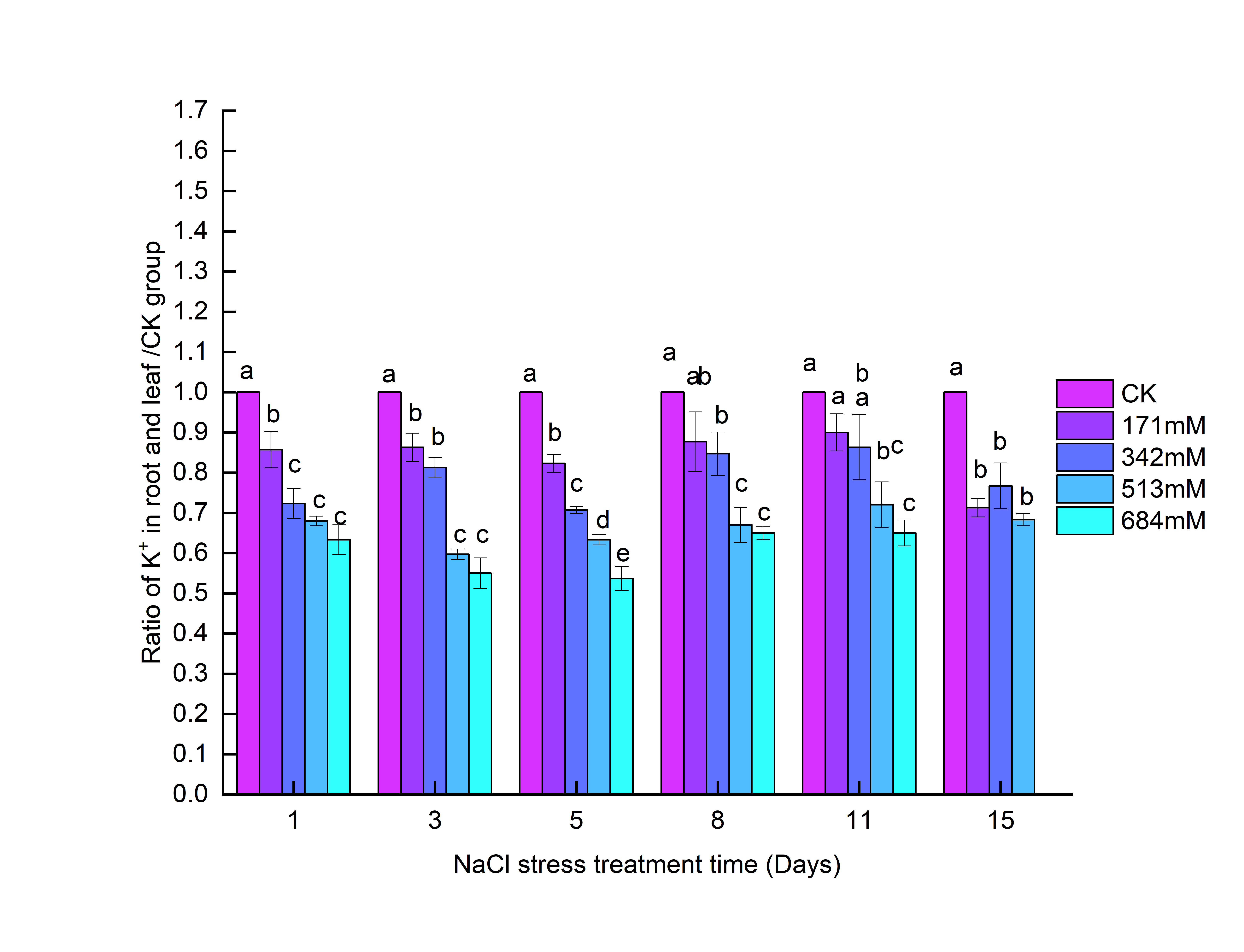


**Figure S12** Ratio of K^+^ to CK in roots/leaves of *Salix* *matsudana*.(data in Fig.4E)


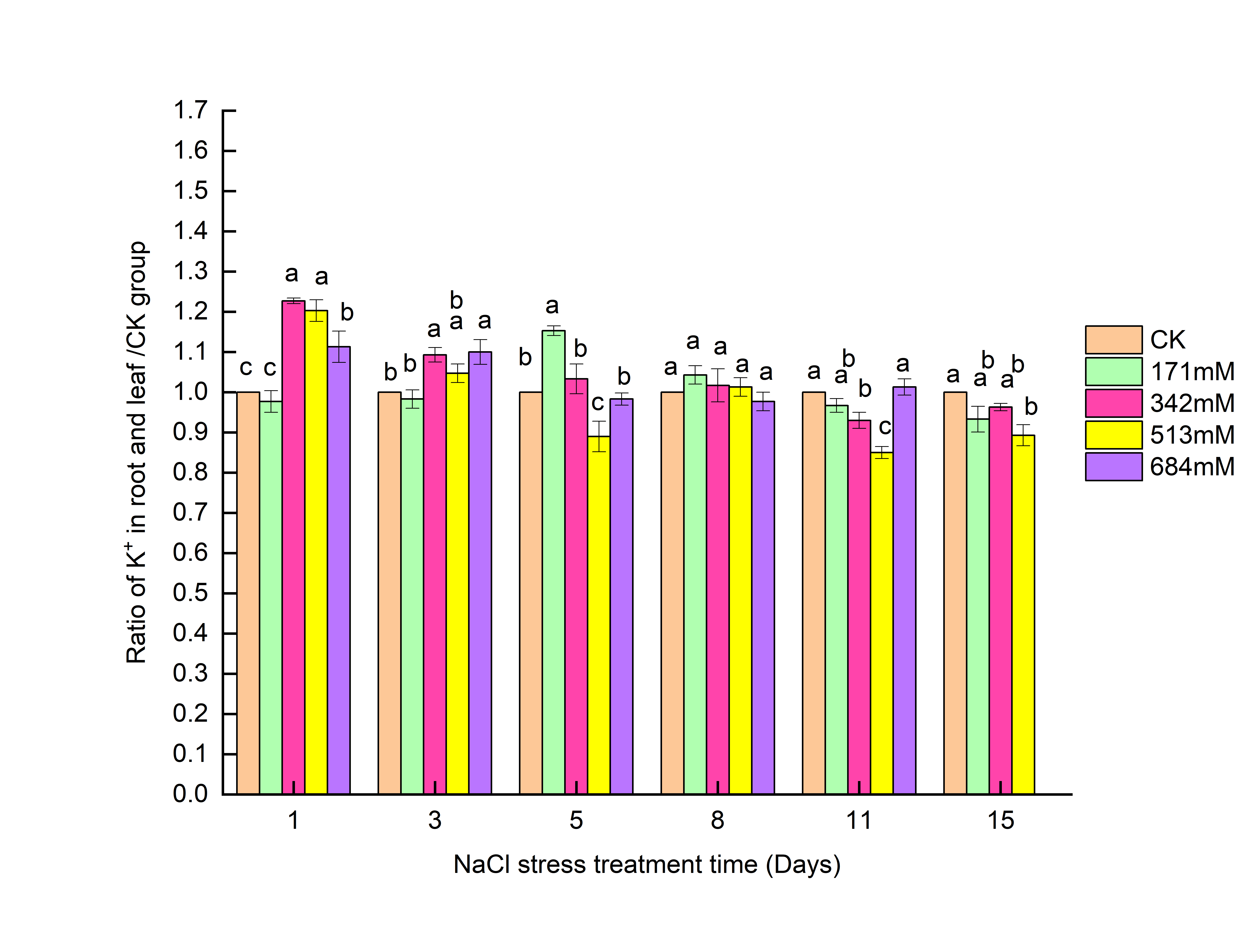


**Figure S13** Ratio of K^+^ to CK in roots/leaves of *Salix* *gordejevii*.(data in Fig.4F)


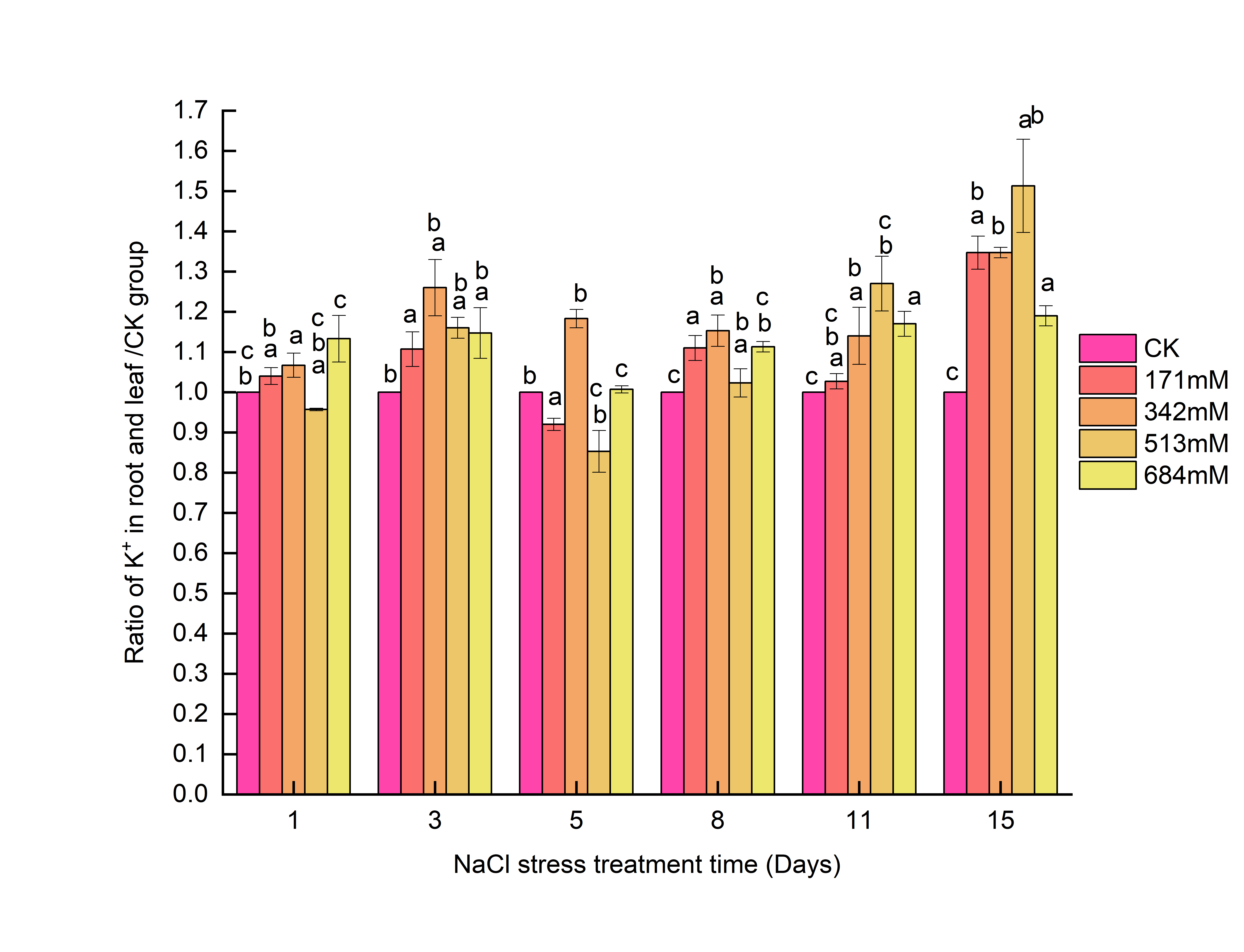


**Figure S14** Ratio of Ca^2+^ to CK in roots/leaves of *Salix* *linearistipularis*.(data in Fig.4G)


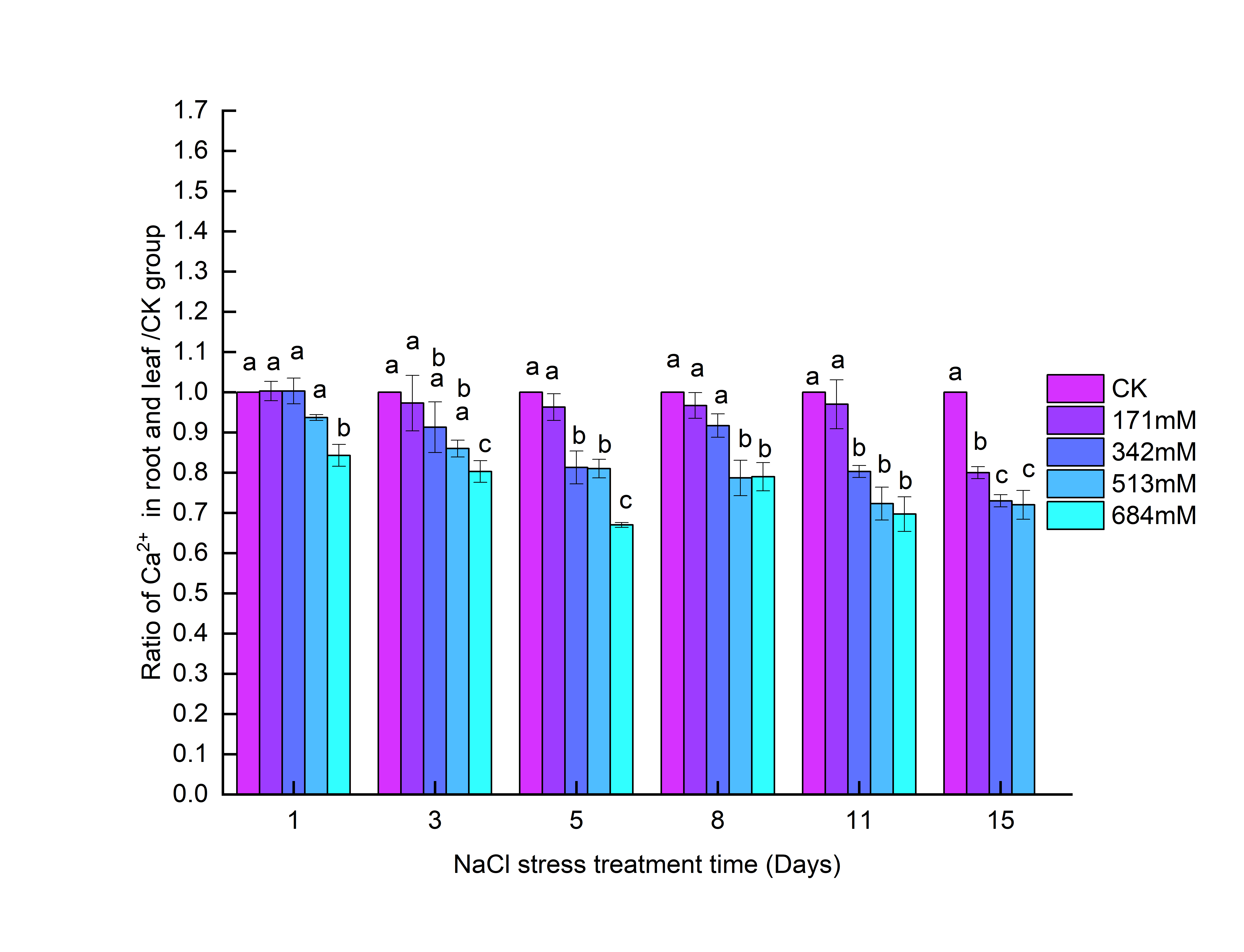


**Figure S15** Ratio of Ca^2+^ to CK in roots/leaves of *Salix* *matsudana*.(data in Fig.4H)


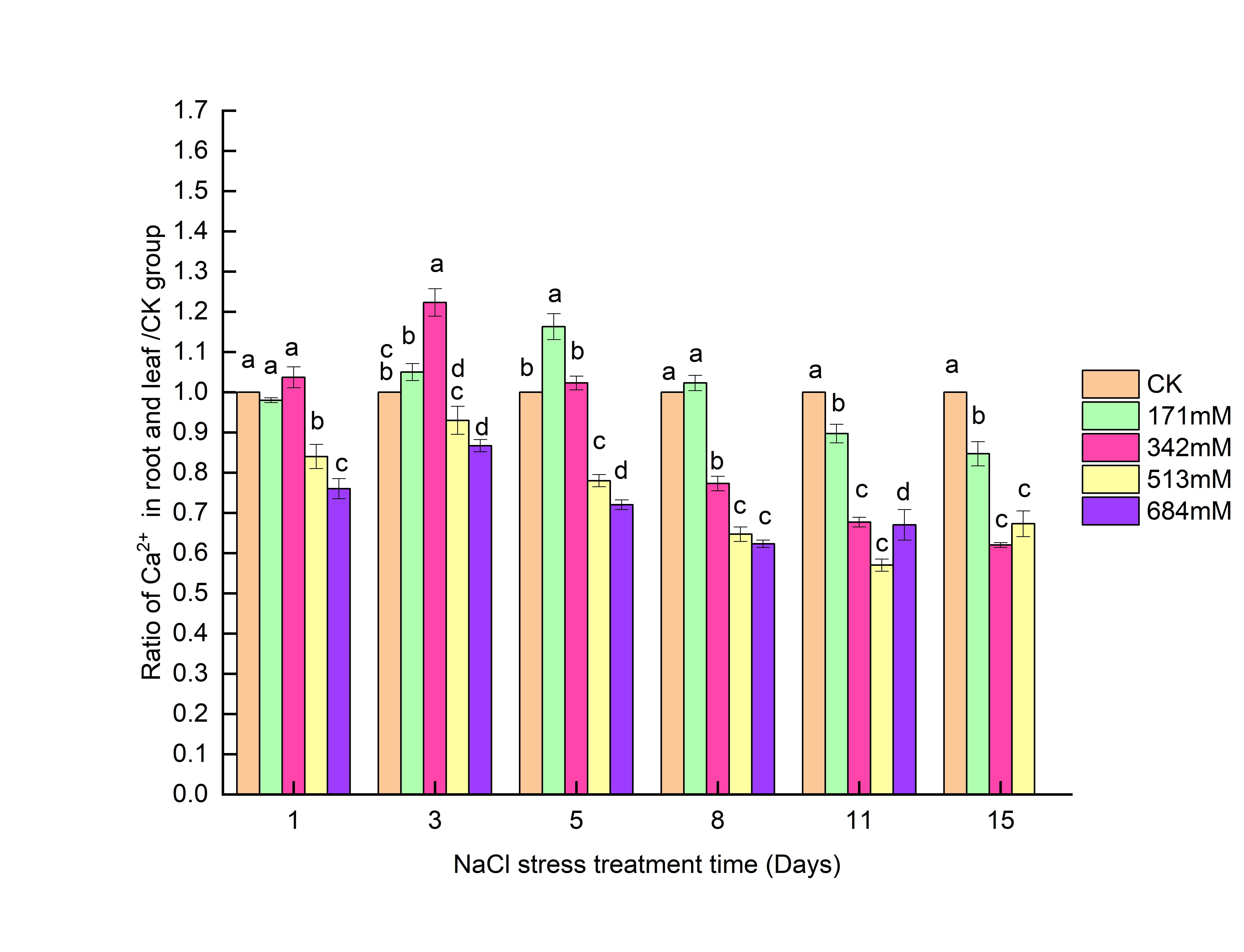


**Figure S16** Ratio of Ca^2+^ to CK in roots/leaves of *Salix* *gordejevii*.(data in Fig.4I)


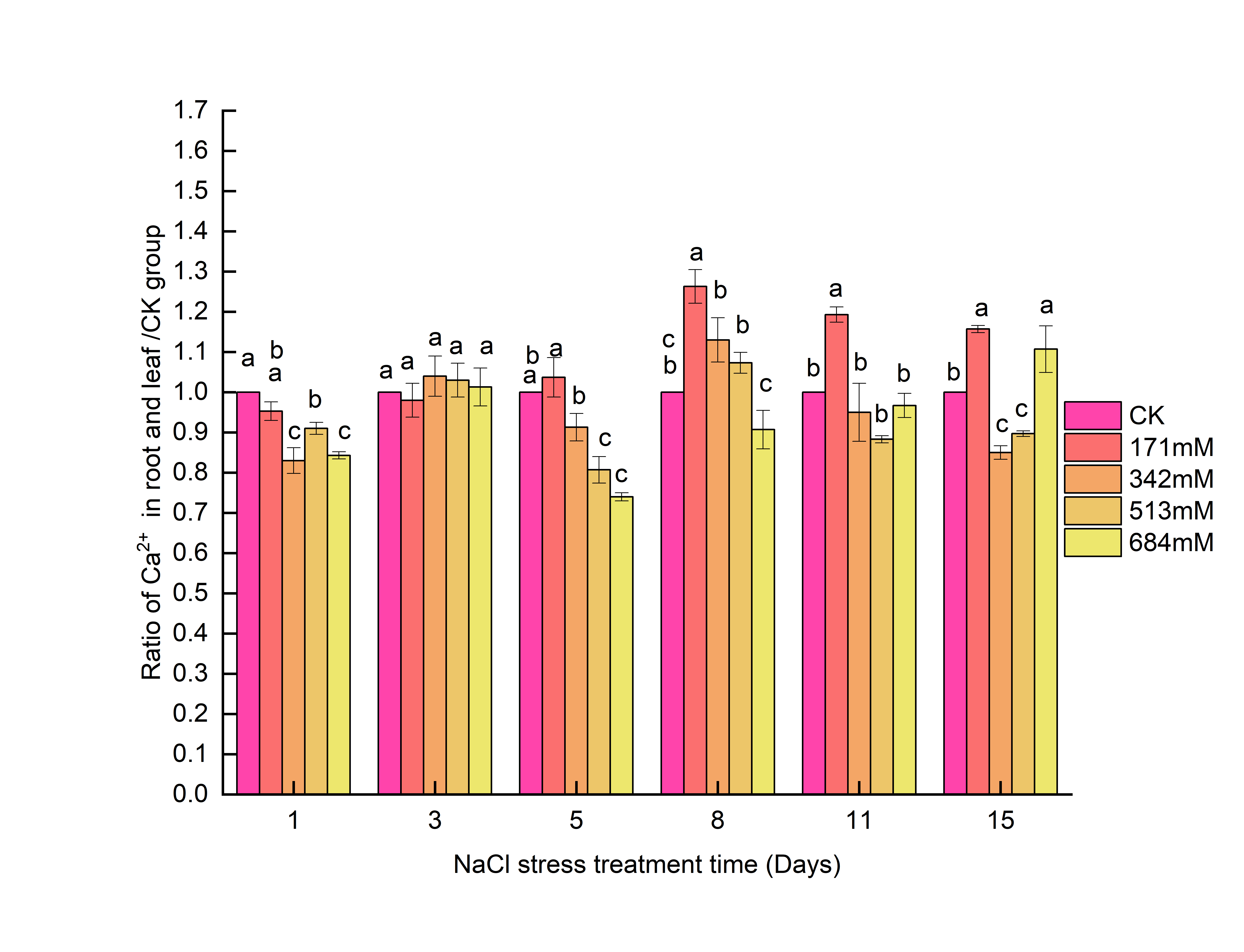


**Figure S17** Changes of K^+^ absorption and transport in roots and leaves of *Salix* *linearistipularis*.(data in Fig.5A)


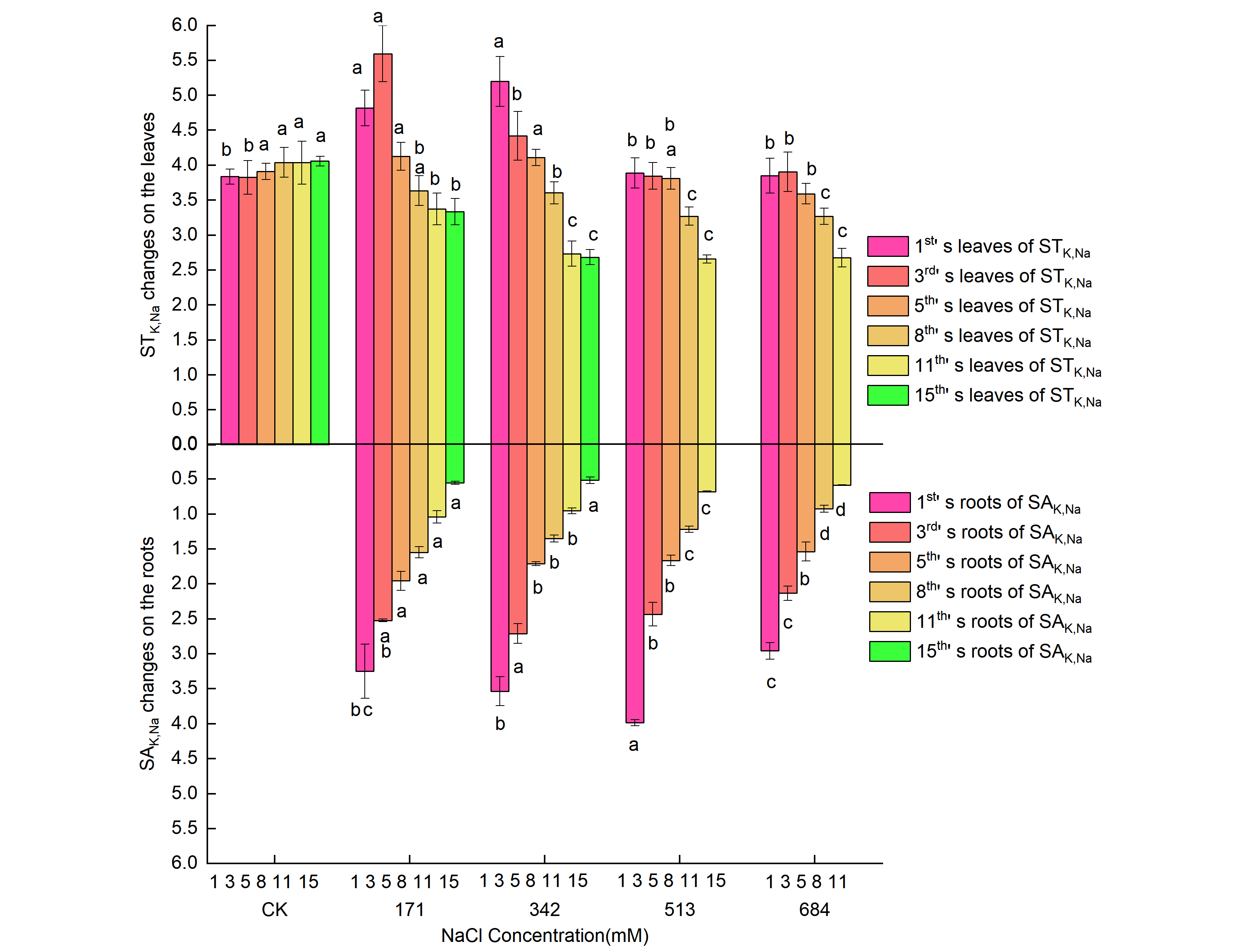


**Figure S18** Changes of K^+^ absorption and transport in roots and leaves of *Salix* *matsudana*.(data in Fig.5B)


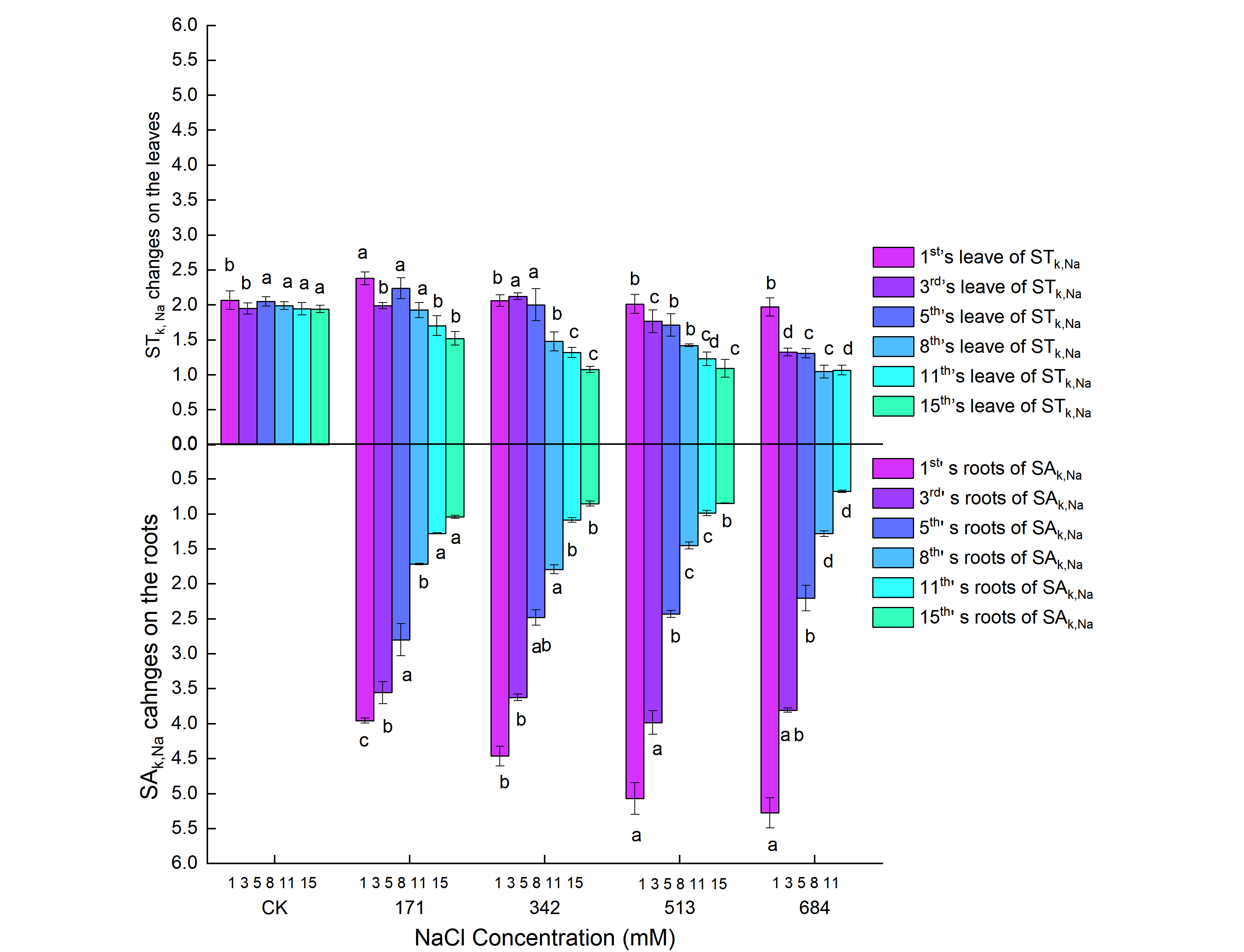


**Figure S19** Changes of K^+^ absorption and transport in roots and leaves of *Salix* *gordejevii*.(data in Fig.5C)


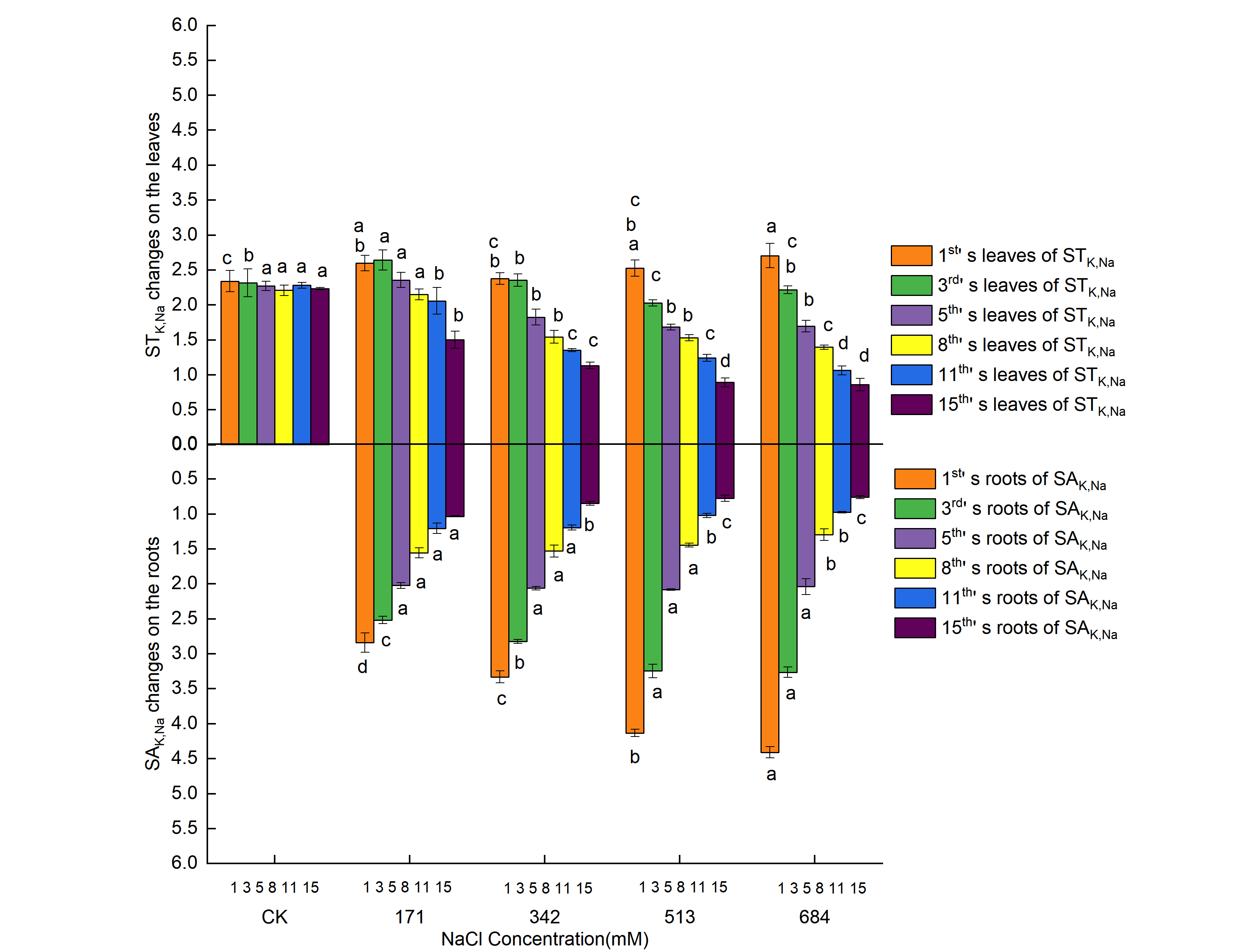


**Figure S20** Changes of Ca^2+^ absorption and transport in roots and leaves of *Salix* *linearistipularis*.(data in Fig.6A)


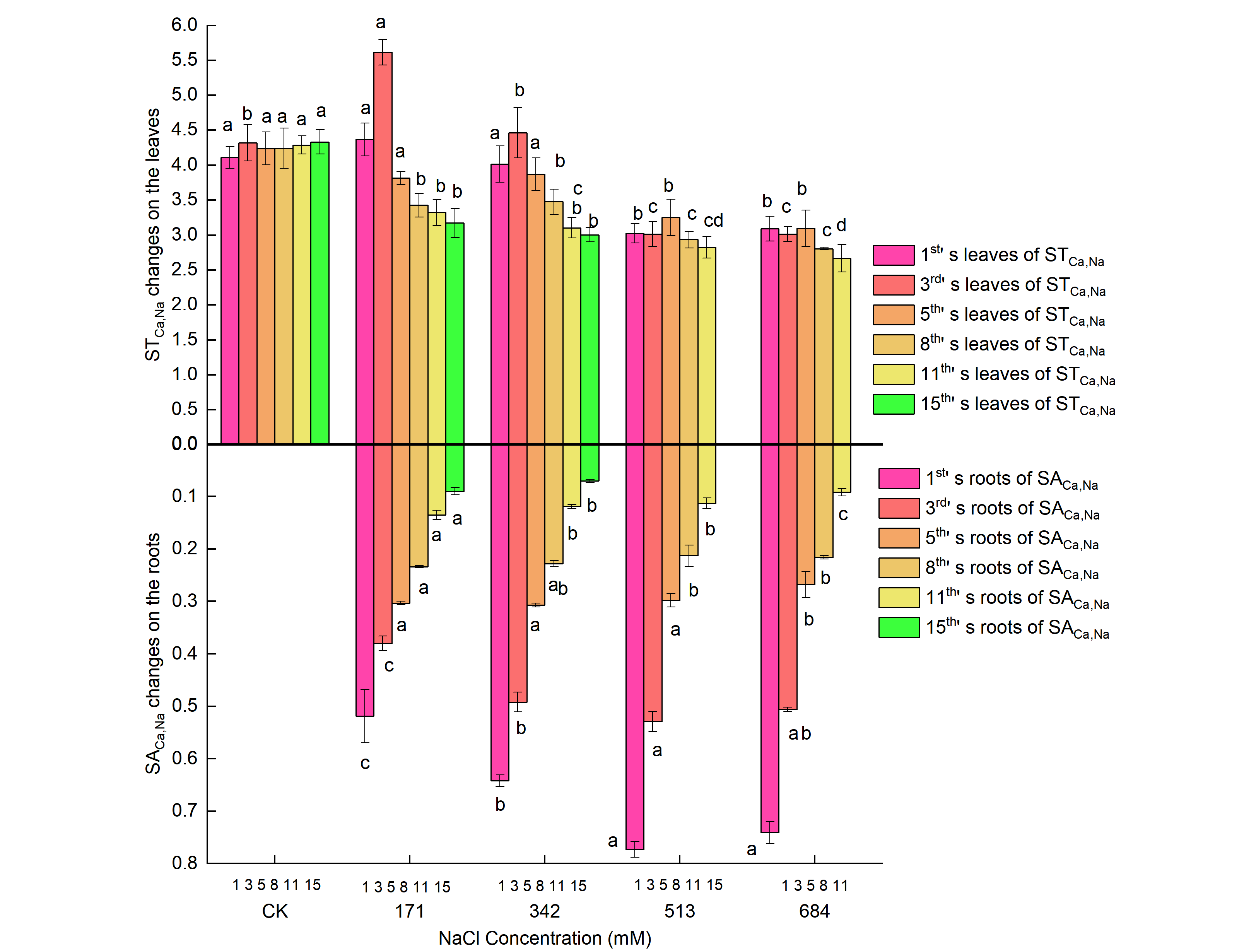


**Figure S21** Changes of Ca^2+^ absorption and transport in roots and leaves of *Salix* *matsudana*.(data in Fig.6B)


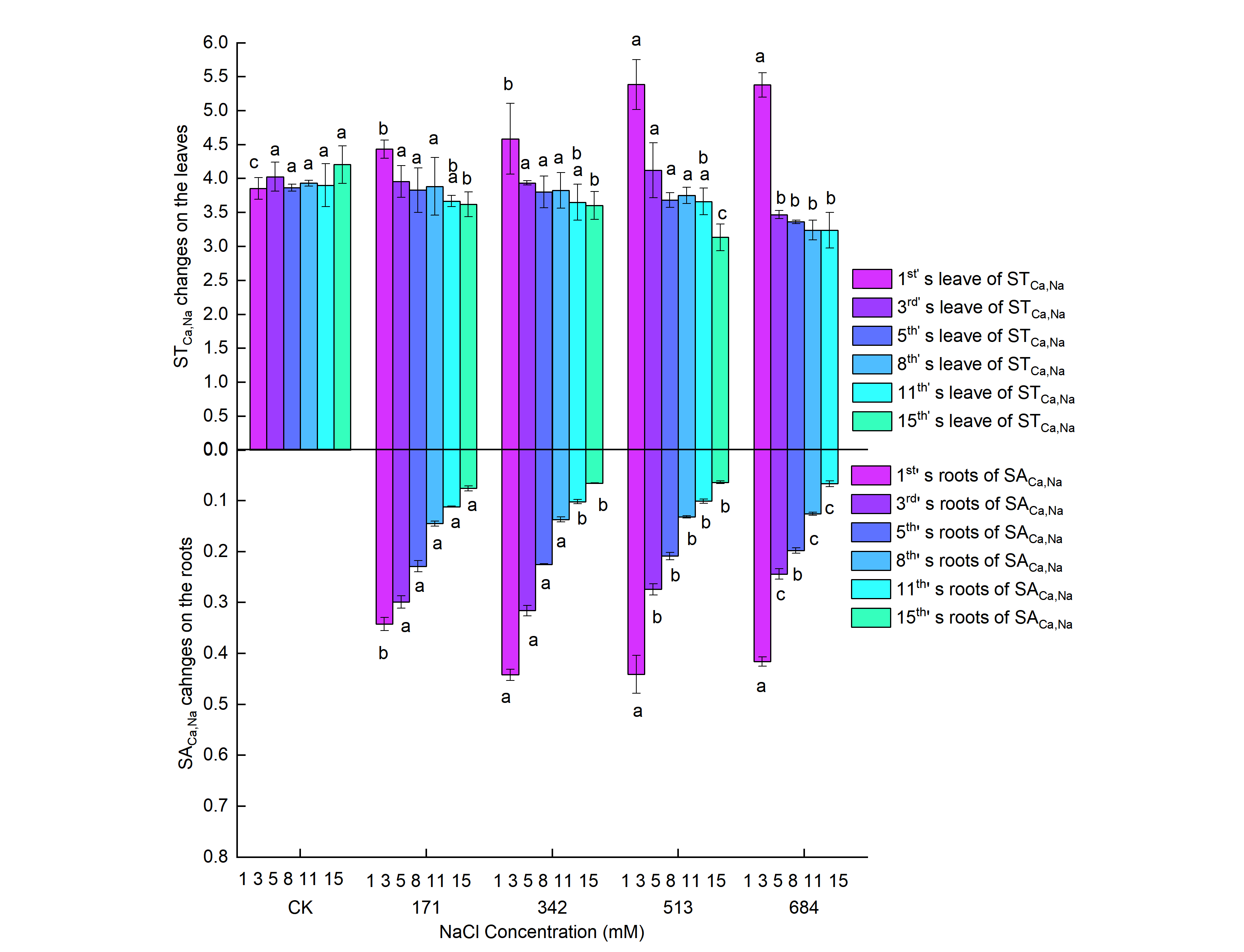


**Figure S22** Changes of Ca^2+^ absorption and transport in roots and leaves of *Salix* *gordejevii*.(data in Fig.6C)


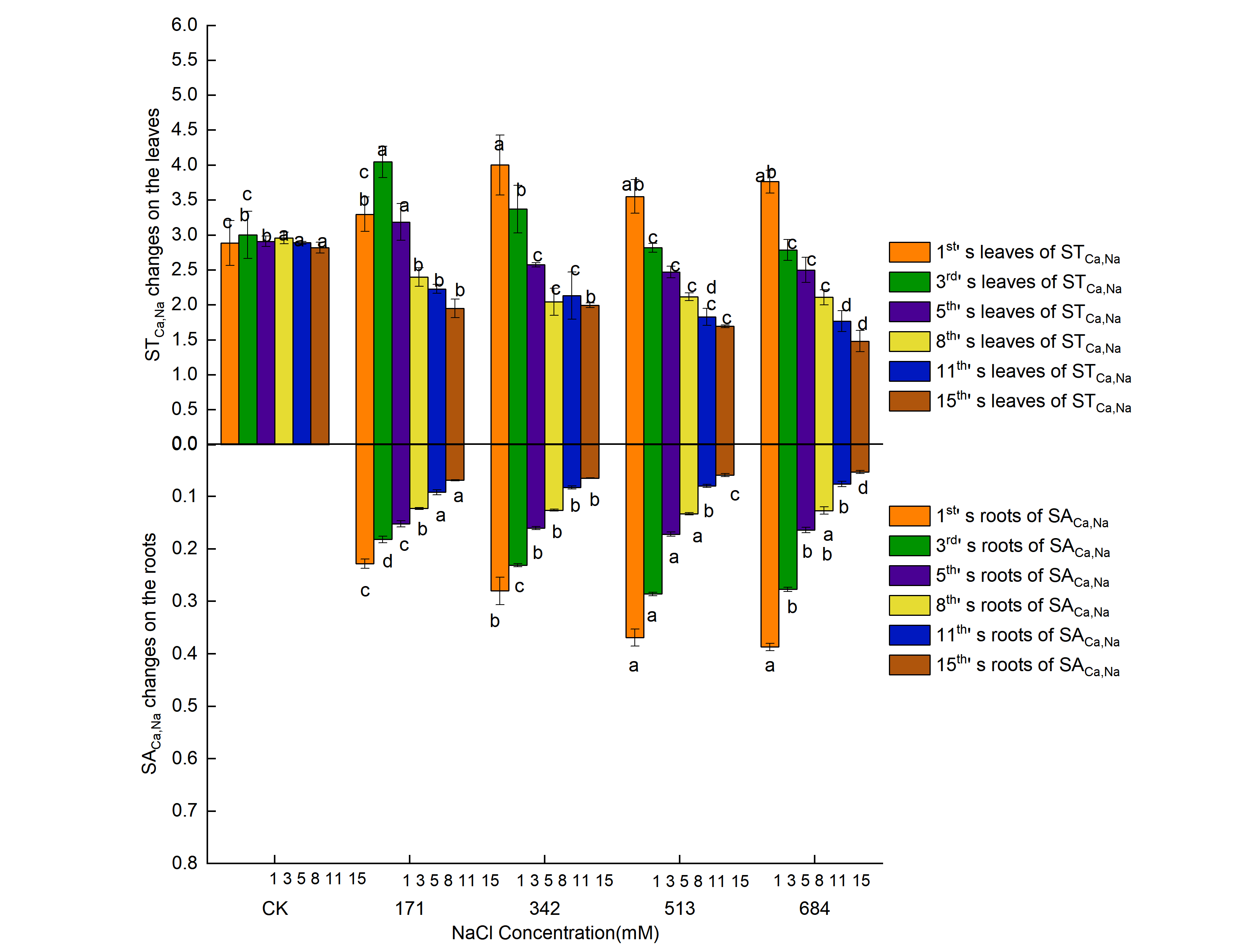


**Figure S23** Changes of Na^+^/ K^+^ in roots and leaves of *Salix* *linearistipularis*.(data in Fig.7A)


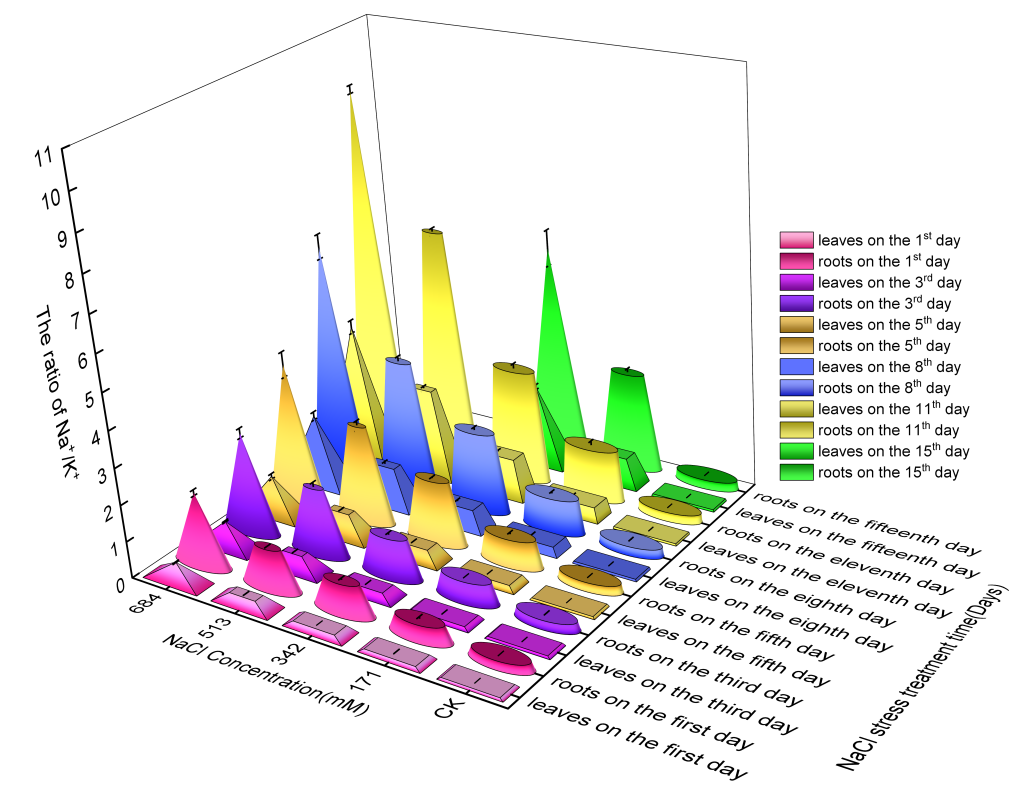


**Figure S24** Changes of Na^+^/ K^+^ in roots and leaves of *Salix* *matsudana*.(data in Fig.7B)


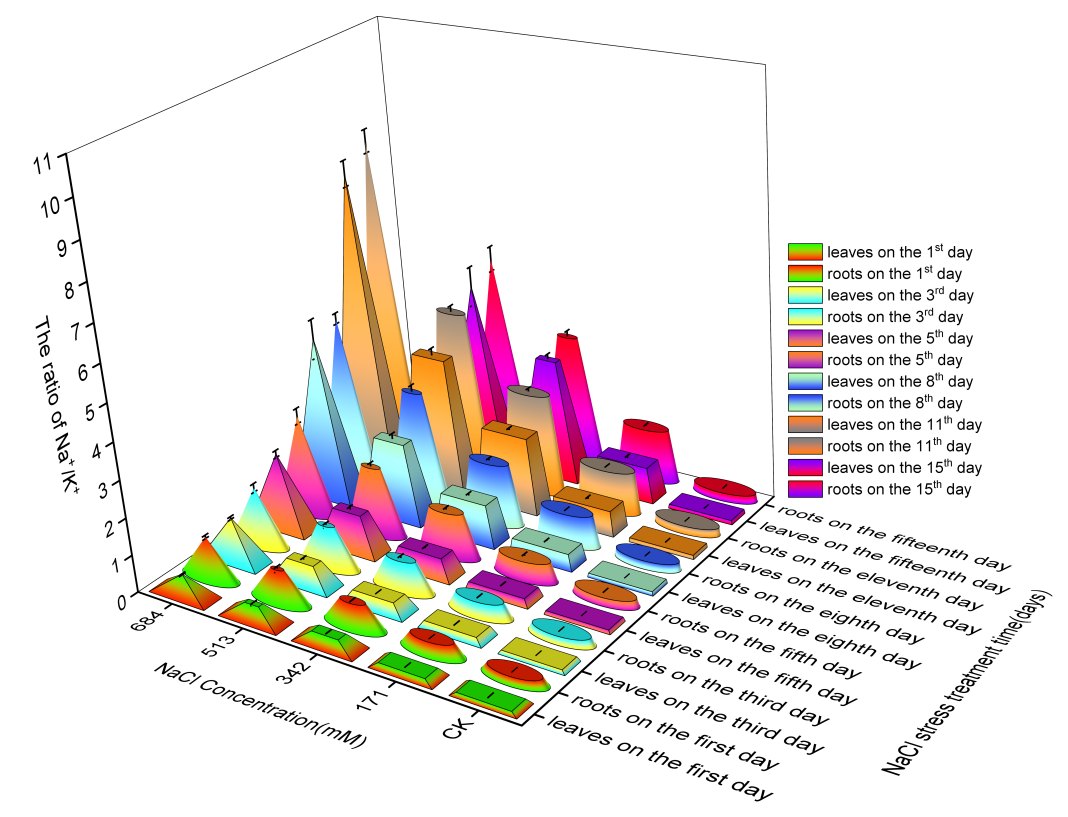


**Figure S25** Changes of Na^+^/ K^+^ in roots and leaves of *Salix* *gordejevii*.(data in Fig.7C)


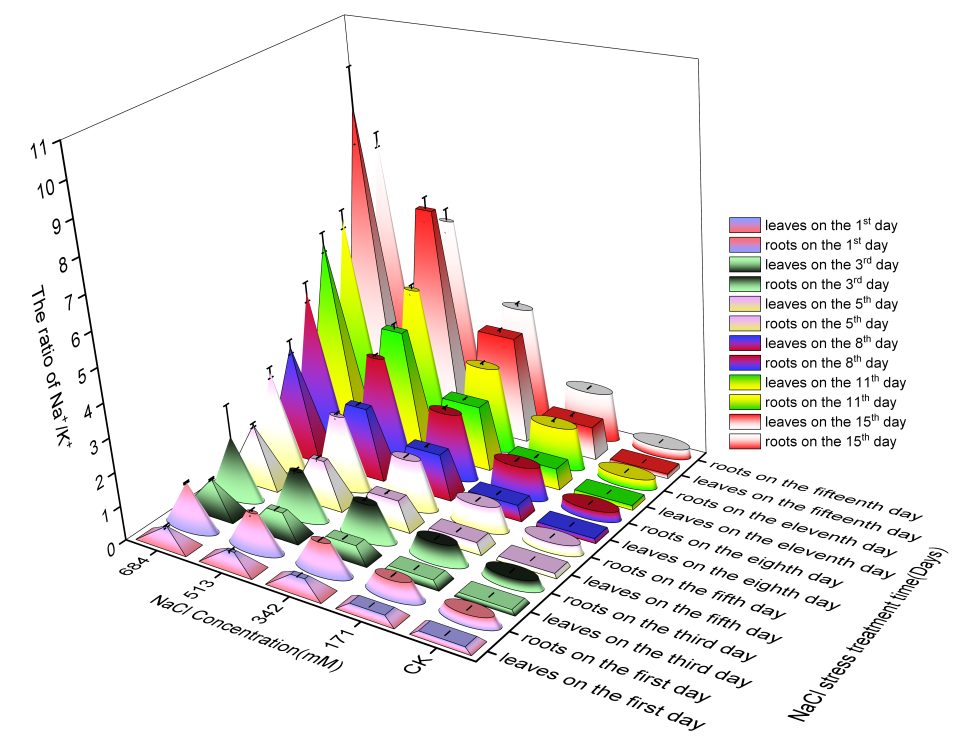


**Figure S26** Changes of Na^+^/ Ca^2+^ in roots and leaves of *Salix* *linearistipularis*.(data in Fig.8A)


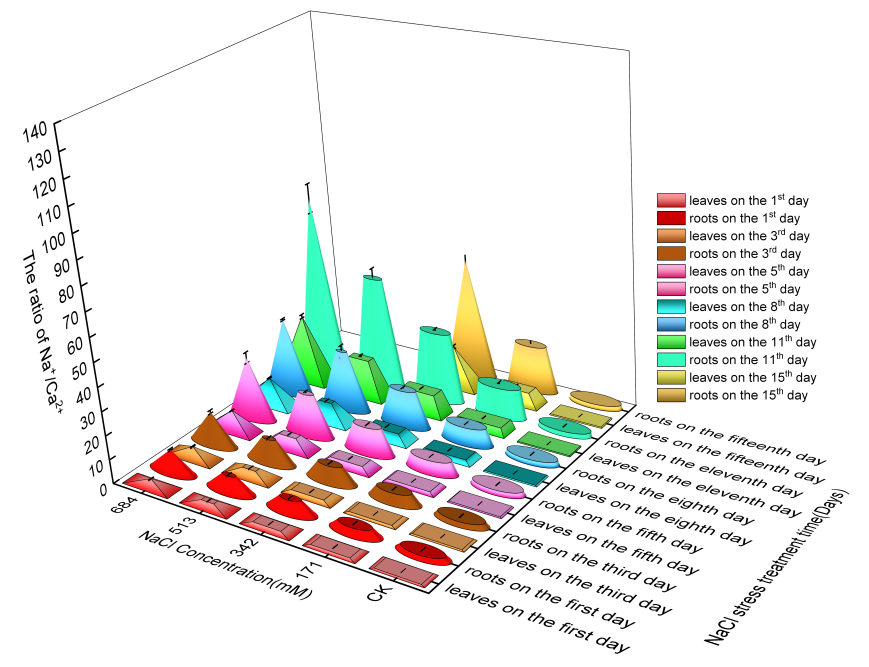


**Figure S27** Changes of Na^+^/ Ca^2+^ in roots and leaves of *Salix* *matsudana*.(data in Fig.8B)


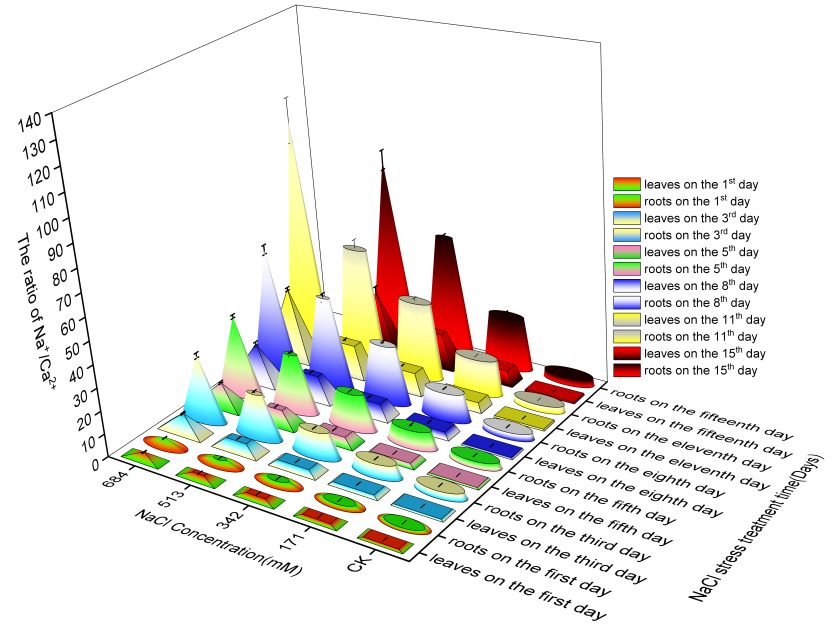


**Figure S28** Changes of Na^+^/ Ca^2+^ in roots and leaves of *Salix* *gordejevii*.(data in Fig.8C)


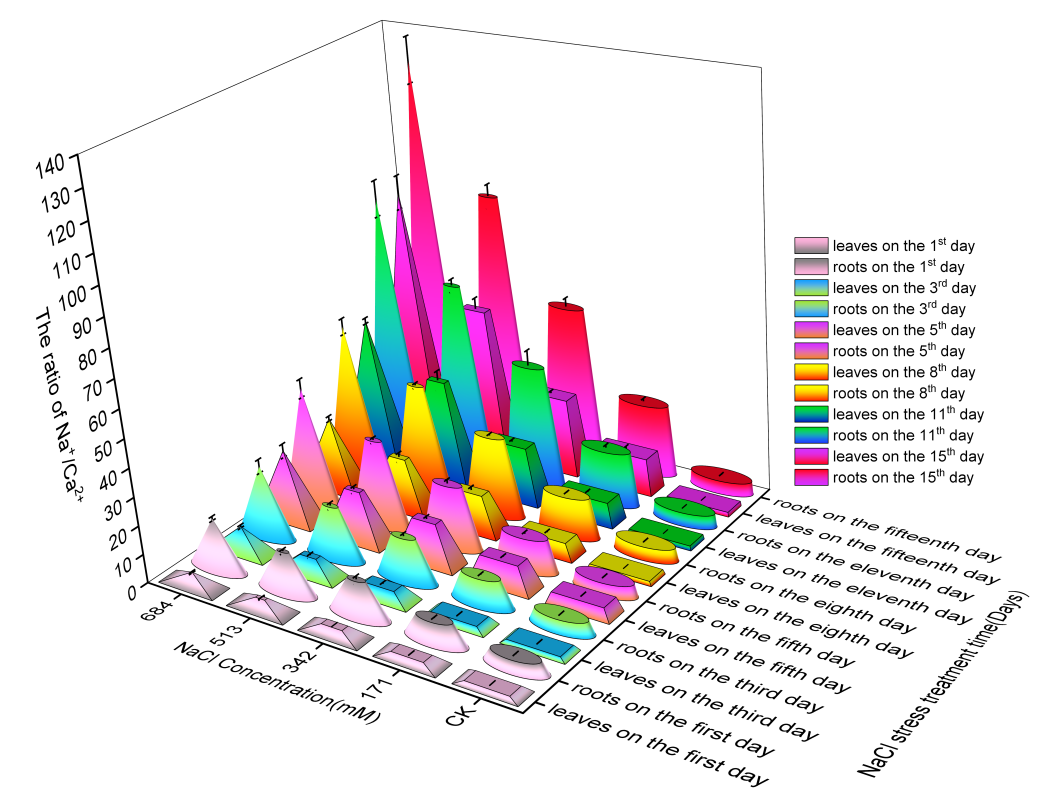


**Figure S29** Correlation of Na^+^in roots and leaves of three willows under salt stress.(data in Fig.9A)


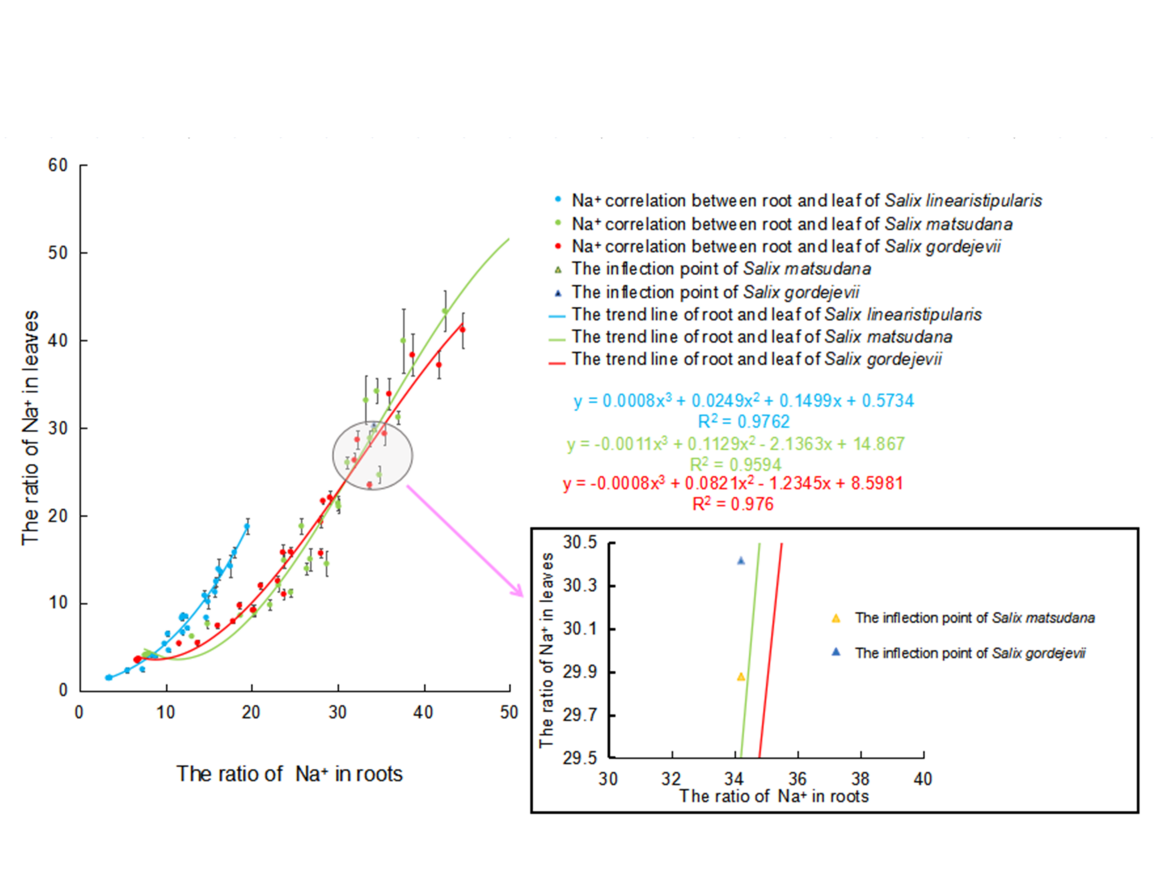


**Figure S30** Correlation of Na^+^/K^+^ in roots and leaves of three willows under salt stress.(data in Fig.9B)


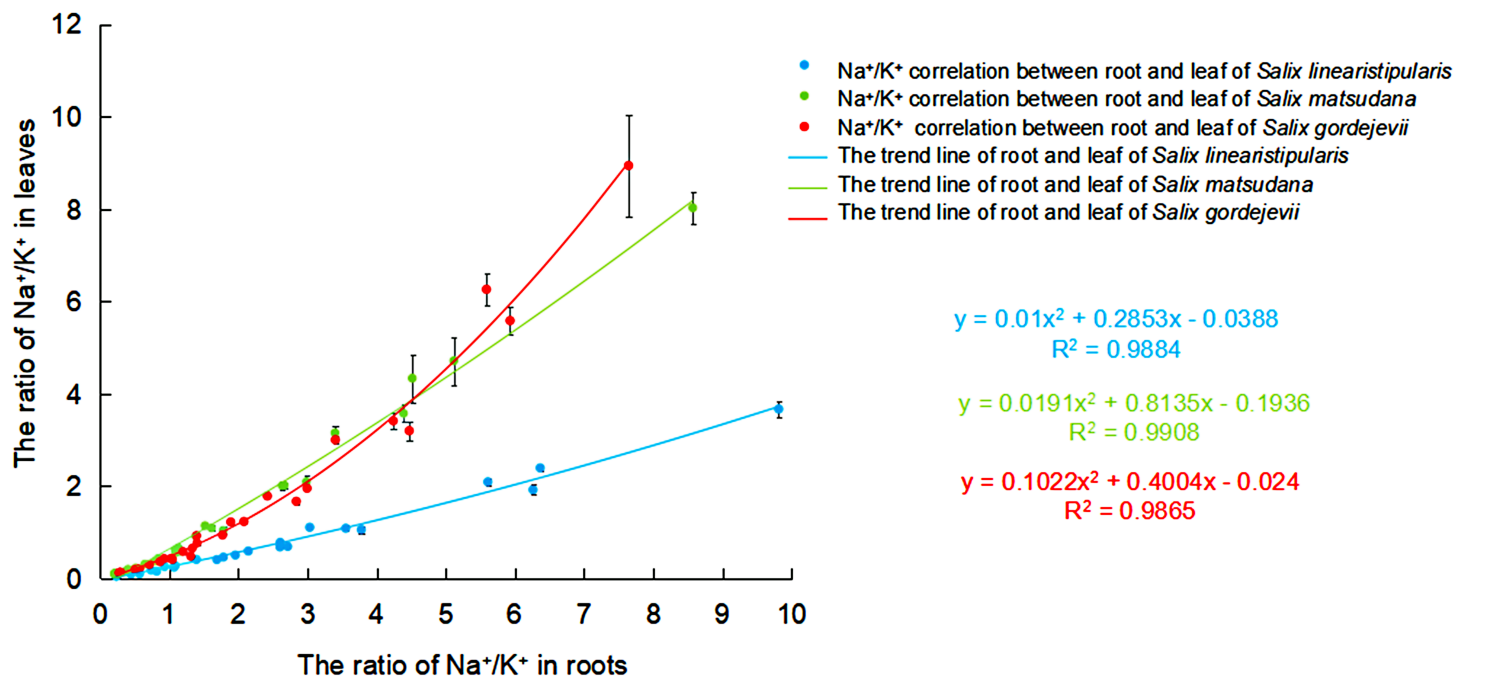


**Figure S31** Correlation of Na^+^/Ca^2+^ in roots and leaves of three willows under salt stress.(data in Fig.9C)


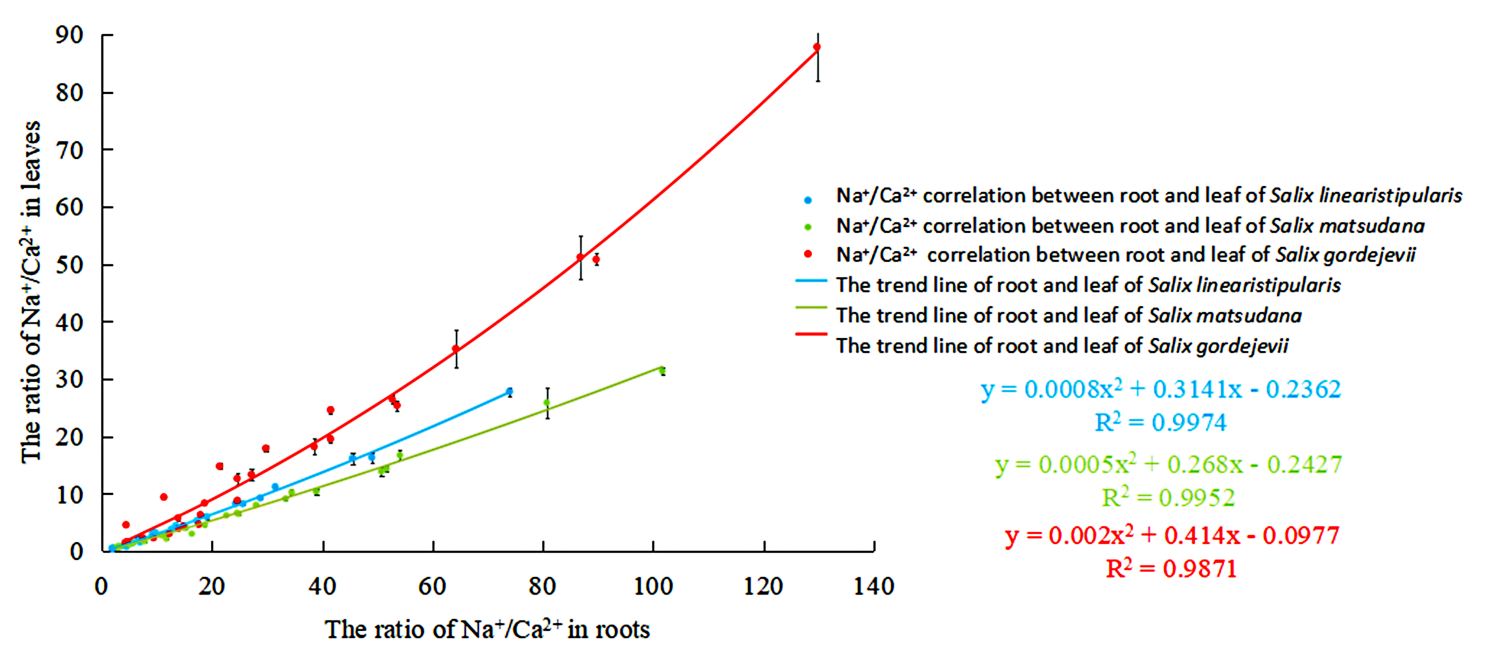

Supplement: Supplementary file 1 [file Data_Sheet_1.docx]
