# Supplementary figures and images for: Ion absorption, distribution and salt tolerance threshold of three willow species under salt stress
Source: Front Plant Sci. 2022 Aug 2;13:969896. doi: 10.3389/fpls.2022.969896 (PMC9379094; doi:10.3389/fpls.2022.969896)

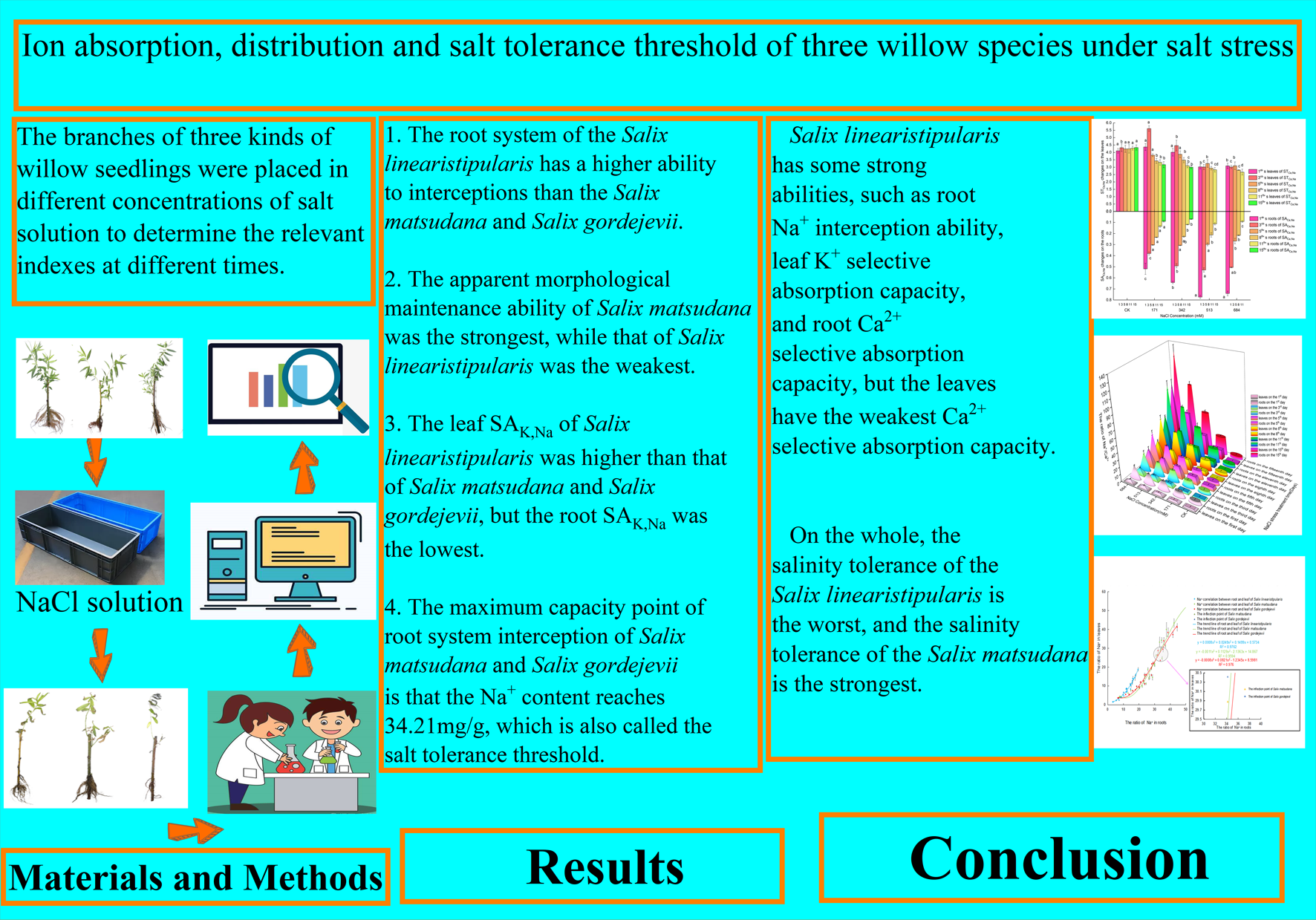

Supplement: Supplementary file 2 [file Image_1.tif]
